# A potent phenylalkylamine disrupts mycobacterial membrane bioenergetics and augments bactericidal activity of bedaquiline

**DOI:** 10.1016/j.isci.2025.112915

**Published:** 2025-06-18

**Authors:** Zheng Yen Phua, Ming Li, Azhar Ali, Cedric Cheng Sheng Cheong, Kai Jie Goh, Marcus Yi Kang Seto, Amos Shi Ying Ng, Jickky Palmae Sarathy, Boon Cher Goh, Mei Lin Go, Wai Keung Chui, Thomas Dick, Yulin Lam

**Affiliations:** 1 Department of Chemistry, National University of Singapore, 3 Science Drive 3, Singapore 117543, Singapore; 2 Department of Microbiology and Immunology, Yong Loo Lin School of Medicine, National University of Singapore, 14 Medical Dr, Singapore 117599, Singapore; 3 Cancer Science Institute of Singapore, Centre for Translational Medicine, 14 Medical Drive, Singapore 117599, Singapore; 4 Center for Discovery and Innovation, Hackensack Meridien Health, 111 Ideation Way, Nutley, NJ 07110, USA; 5 NUS Centre for Cancer Research (N2CR), 14 Medical Drive, Singapore 117599, Singapore; 6 Department of Medicine, Yong Loo Lin School of Medicine, National University of Singapore, 1E Kent Ridge Road, Singapore 119228, Singapore; 7 Department of Pharmacy and Pharmaceutical Sciences, Faculty of Science, National University of Singapore, 18 Science Drive 4, Singapore 117543, Singapore; 8 Department of Biochemistry, Yong Loo Lin School of Medicine, National University of Singapore, 8 Medical Dr, Singapore 117596, Singapore

**Keywords:** Chemistry, Biological sciences, Energy engineering

## Abstract

Clinically efficacious combination therapies capable of impeding resistance are widely sought for the treatment of mycobacterial infections. Here, we described structural modifications of the phenylalkylamine scaffold of verapamil to give an analog with more than 10-fold greater growth inhibitory activity than verapamil against *Mycobacterium tuberculosis*, *M. bovis* BCG, and *M. abscessus abscessus (Mab abscessus).* The analog synergized with the F_1_F_o_-ATP synthase inhibitor bedaquiline in checkerboard assays and augmented the bactericidal properties of bedaquiline against *M. bovis* BCG and *Mab abscessus*. Using live cell bioorthogonal imaging techniques, *in vitro* biochemical and genetic assays, the bactericidal activity of the analog is attributed to the perturbation of membrane bioenergetics and disruption of mycobacterial respiration. Overall, its promising activity profile, mode of action and synergistic interaction with bedaquiline support further exploration of the phenylalkylamine scaffold as a valued source of potential leads for antimycobacterial drug discovery.

## Highlights

• Structural optimization of verapamil against mycobacteria led to a potent analog PhA2
• PhA2 is the most potent verapamil analog against mycobacteria reported to date
• PhA2 augmented the bactericidal activity of bedaquiline at non-killing concentrations
• PhA2 disrupted mycobacterial membrane integrity and perturbed membrane bioenergetics

## Graphical abstract

**Figure:**
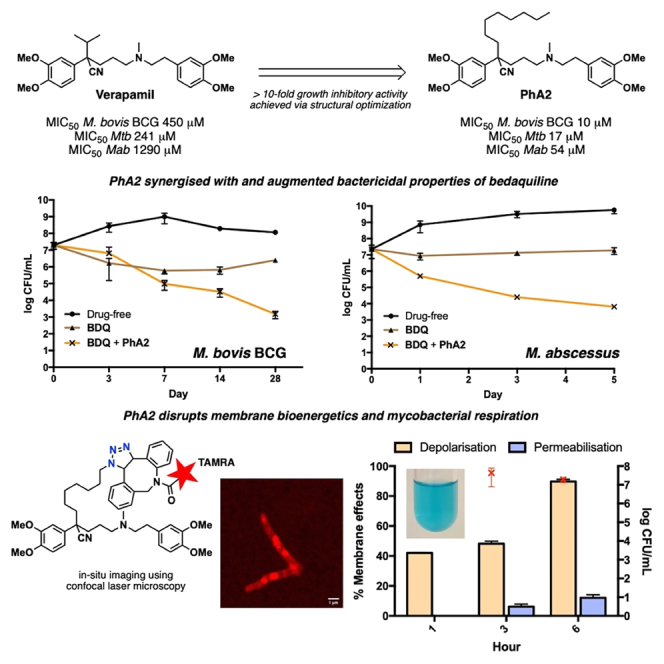

## Introduction

Several species within the genus *Mycobacterium* cause debilitating, even fatal pulmonary infections in humans. The most notable is *Mycobacterium tuberculosis* (*Mtb*), the causative organism of tuberculosis (TB) which until COVID-19 was the leading cause of death by a single infectious agent.^1^ Species collectively called non-tuberculous mycobacteria (NTM) also pose an incipient threat to global health.^2^ Pulmonary infections caused by NTM have increased at an alarming rate in affluent nations, particularly among the aged, immunocompromised individuals and those with structural lung damage.^3^ Encouragingly, more effective treatment regimens now exist for TB, notably the optimized drug combination BPaL comprising bedaquiline (BDQ), pretomanid and linezolid for drug resistant TB^4^^,^^5^ and a 4-drug regimen that reduces treatment of uncomplicated TB from six months to four.^6^ Despite these advancements, more effective therapies are needed, particularly for NTM pulmonary diseases which still lack reliable cures.^3^^,^^7^

**Figure 1:**
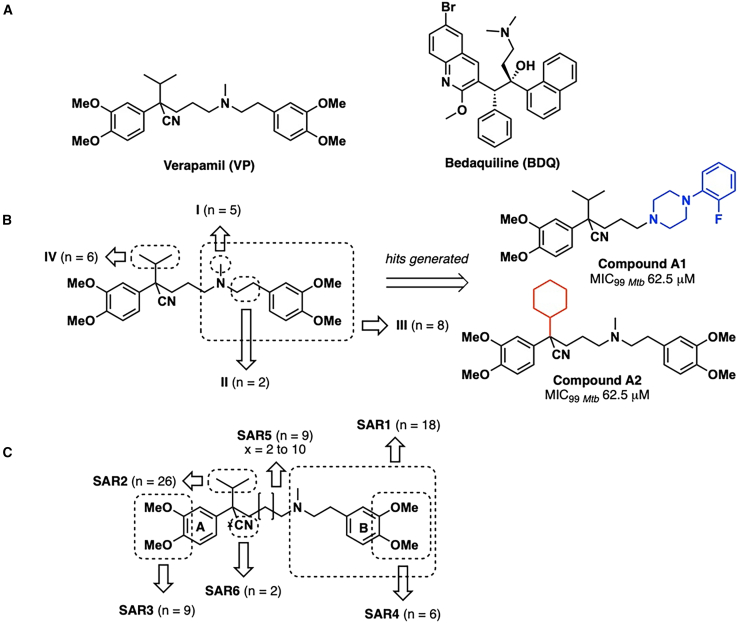
Structural modifications to interrogate the antimycobacterial activity of the phenylalkylamine scaffold in VP (A) Structures of verapamil (VP) and bedaquiline (BDQ). (B) Reported sites on the phenylalkylamine scaffold in VP investigated for structure-activity relationship against *Mtb*.^8^ Structures of hits A1 and A2. n represents number of compounds evaluated at each site. MIC_99__*Mtb*_ is the minimum concentration required to inhibit growth of *Mtb* H37RV by 99%. (C) SAR1–SAR6 are modification sites on the scaffold investigated in this report. Modifications are as follows: SAR1: embedding aliphatic N in ring structure; SAR2: replacement of isopropyl by alkyl, alkenyl, arylalkyl, cycloalkyl groups; SAR3: replacement of 3,4-dimethoxy (ring A) with 3,4-dichloro or 4-chloro; SAR4: As in SAR3 but on ring B and no mono-Cl substitution; SAR5: homologation of alkyl side chain x = 2–10; SAR6: nitrile replacement.

Here, we present our findings on the antimycobacterial activity of phenylalkylamines which are structurally related to verapamil (VP) (Figure 1A), an FDA approved calcium channel blocker. Our interest in this scaffold is 2-fold. First, the phenylalkylamine scaffold with its protonated amino group and lipophilic phenyl rings is a prototypal cationic amphiphile, a motif found in membrane-damaging antibacterial agents.^9^^,^^10^ The affinity of these agents for the bacterial membrane is driven by hydrophobic interactions between their lipophilic residues and the lipid rich matrix of the bacterial cell envelope, and reinforced by electrostatic attractions between the positively charged scaffold and negatively charged phospholipids which predominate in bacterial membranes.^11^^,^^12^^,^^13^ These interactions disrupt membrane integrity, leading to a loss of its energized state and eventual cell death.^9^ VP exemplifies this mode of action where corroboratory evidence of proton motive force (PMF) disruption and induction of membrane stress reiterate a mode of action centered on the disruption of mycobacterial membrane energetics.^14^

Second, the bactericidal activity of VP extends to both replicating and drug-tolerant non-replicating bacteria.^14^ Dual targeting of these bacterial populations by VP underscores its potential to shorten treatment and forestall the emergence of resistant strains. Additionally, VP augments the bactericidal activity of BDQ (Figure 1A) which disrupts mycobacterial energy metabolism by inhibiting F_1_F_o_-ATP synthase.^14^^,^^15^ Synergism with BDQ affirms the bioenergetic targeting effects of VP and is congruent with the established benefits of combining such inhibitors to expedite mycobacterial clearance.^16^ Unfortunately, as VP is structurally optimized for calcium channel inhibition, it is understandably a weak antibacterial, necessitating high micromolar concentrations (∼500 μM, 227 μg/mL) to inhibit mycobacterial growth.^8^^,^^14^^,^^17^ Efforts at optimizing its anti-TB activity have yet to yield potent derivatives,^8^ highlighting the need for a more granular approach to structure-activity optimization. Here, we investigated structural iterations of the cationic amphiphilic motif in the phenylalkylamine scaffold which is posited to play a pivotal role in disrupting membrane energetics and promoting bactericidal activity. Encouragingly, this approach has yielded phenylalkylamines that were significantly more potent (10- to 60-fold) than VP against various mycobacterial species. Moreover, *in situ* imaging with a biorthogonal chemical probe molecule and *in vitro* assays provide corroborative evidence to support targeting of mycobacterial energy generation as causal to bactericidal activity.

## Results

### SAR studies yielded phenylalkylamines with 20-fold improvement in growth inhibitory activity against *M. bovis* BCG

To our best knowledge, only one report described the correlation between structural modifications and antimycobacterial activity of the phenylalkylamine scaffold in VP.^8^ Figure 1B summarizes the four modification sites (I-IV) investigated in that report. Of these, only two sites (III and IV) yielded compounds more potent than VP, namely A1 and A2 (MIC_99 Mtb_ 62.5 μM, Figure 1B). A1 was derived from site III in which the tertiary N was embedded in various nitrogen heterocycles and A2 was based on a site IV modification in which substitution at the chiral carbon was varied. Embedding the tertiary N in a ring reduces flexibility and affects overall shape while replacing isopropyl at the chiral carbon with substituents of varying lipophilicities, bulkiness or flexibility alters physicochemical properties which affect drug-likeness and biological activity.^18^ The modifications undertaken at both sites are standard medicinal chemistry approaches.^19^ Although A1 and A2 were more potent than VP, their moderate MICs and the modestly sized library from which they were derived led us to ask if greater gains in potency could be achieved if modifications were made at other sites on the VP scaffold. Hence, we continued to probe the promising sites III and IV (denoted as SAR1 and SAR2, respectively) while extending our search to four other unexplored parts of the scaffold, namely substitutions on ring A (SAR3) and ring B (SAR4), varying the alkyl chain length between the tertiary N and the chiral center (SAR5) and nitrile substitution (SAR6) (Figure 1C). These modifications are based on established medicinal chemistry principles.^19^

In all, 70 compounds were synthesized and evaluated against *Mycobacterium bovis* Bacillus Calmette-Guérin (BCG) for growth inhibition (Table S1). *M. bovis* BCG is widely used as an avirulent surrogate of the pathogenic *Mtb* for the preliminary screening of hit/lead compounds. Their genomes share 99.95% identity at the nucleotide level and 97% of *Mtb* proteins have conserved orthologs in *M. bovis* BCG.^20^^,^^21^^,^^22^^,^^23^

To recapitulate, our design strategy is directed toward identifying hit compounds by optimizing the anti-TB activity of the phenylalkylamine scaffold of VP. To this end, we probed selected sites on the scaffold with modifications that are standard to the medicinal chemistry canon. These are functional group substitution, cyclization, homologation, and ring substitution.^19^ These modifications will affect important physicochemical properties (lipophilicity, permeability, and solubility) of the molecule with consequential effects on its interactions with biological targets.^18^ Notably, a wide range of lipophilicities was intentionally introduced into the final compounds, prompted in part by the complex architecture of the mycobacterial cell envelope, of which approximately 40% of the dry cell mass is estimated to be lipids.^24^ The unique topography and composition of the cell wall pose a formidable barrier to entry by external agents and hence, constitute a serious challenge to the development of anti-TB drugs.^25^ We posited that lipophilic scaffolds are better placed to transverse the cell envelope and hence gain access to the protein targets embedded within the membranes. To this end, 80% of the synthesized compounds were more lipophilic than VP based on clogP values (Table S1).

To assess the outcomes of our structural modifications, we shortlisted 16 compounds (PhA1-16) that were at least 7 times more potent than VP, corresponding to MIC_50BCG_ < 30 μM (Table 1). First, we noted that no compound was derived from SAR6 (nitrile replacement) which is likely due to the limited compounds (*n* = 2) investigated at this site. Second, the dimethoxy to dichloro substitutions in SAR3 and SAR4 increased activity significantly as shown by PhA7-8 (MIC_50BCG_ 26–27 μM versus VP 210 μM). Even the presence of a single chloro sufficed to enhance activity as seen from A2 (MIC_50BCG_ 36 μM) vis-à-vis the mono-chloro substituted PhA14 (19 μM). These gains in potency are likely due to the lipophilic chloro. Interestingly, replacing the remaining two methoxy groups in PhA7-8 with chloro resulted in tetra-chloro substituted PhA66 (Table S1) which had greatly diminished activity (MIC_50BCG_ 56μM), notwithstanding the gains in lipophilicity. This was again observed in SAR5 (extension of alkyl side chain) where activity peaked at the 10-carbon chain analog (PhA9 MIC_50BCG_ 11 μM) and not the more lipophilic 12-carbon analog PhA10. There is clearly an optimal level of lipophilicity beyond which activity would fall.

**Table 1:** Structures, M. bovis BCG minimum inhibitory concentrations (MIC) and clogP values of VP and compounds with MIC_50 BCG_ < 30 μM

Compound	Structure	MIC_50_^a^	MIC_90_^a^	clogP^b^	Compound	Structure	MIC_50_^a^	MIC_90_^a^	clogP^b^
**VP**^c^		210	550	5.69	**PhA8**		26	45	7.06
**A1**		87	138	5.92	**PhA9**		11	22	8.61
**A2**		36	118	6.44	**PhA10**		18	32	9.45
**PhA1**		12	25	7.28	**PhA11**		14	25	8.65
**PhA2**^d^		10	18	7.86	**PhA12**		16	29	9.23
**PhA3**		11	22	7.58	**PhA13**		12	23	9.35
**PhA4**		23	44	7.28	**PhA14**		19	35	7.26
**PhA5**		17	43	7.38	**PhA15**		21	37	7.67
**PhA6**		17	45	7.11	**PhA16**		29	46	9.02
**PhA7**		27	47	7.06					

a Minimum inhibitory concentrations (μM) required to reduce growth of *M. bovis* BCG by 50% (MIC_50_) and 90% (MIC_90_) compared to untreated mycobacterial cultures. Average of at least 2 separate determinations using the broth dilution method on *M. bovis* BCG cultures.^26^

b clogP values were obtained from ChemDraw version 20.1.

c The MIC_50 BCG_ and MIC_90 BCG_ of VP are 96μg/mL and 250 μg/mL, respectively.

d PhA2 is a reported compound.^27^

Third, most of the shortlisted compounds were from SAR2 (isopropyl replacement), either as a standalone modification (PhA1-6) or combined with SAR3 (PhA11-15). There was a strong preference for side chains that were extended and flexible, as seen from cyclohexylethyl (PhA1, PhA11), *n*-octyl (PhA2, PhA12), and geranyl (PhA3, PhA13). Less favored were bulky (cyclooctyl in PhA4) and shorter, less flexible side chains (halogenated benzyl in PhA5-6). Interestingly, the MICs of PhA11-13 which were modified at SAR2 and SAR3, were broadly comparable to PhA1-3 (SAR2 only) which led us to surmise that isopropyl replacement (SAR2) contributed more to potency than the methoxy to chloro substitution (SAR3).

Lastly, SAR1 (nitrogen in ring) yielded only one active compound PhA16 which had the least favorable MIC_50BCG_ (29 μM) among the shortlisted compounds. There is also an isopropyl to geranyl substitution (SAR2) in PhA16 and although the latter is generally associated with good activity (PhA3, PhA11), this was ostensibly nullified when combined with SAR1 in PhA16.

Taken together, we have identified flexibility and lipophilicity as critical features for potent growth inhibitory activity in the phenylalkylamine scaffold. The activity enhancing effect of flexibility is evident from the advantageous replacement of isopropyl with cyclohexylethyl, *n*-octyl, and geranyl (PhA1-3, 11–13) as well as the extended 10-carbon side chain between the chiral carbon and tertiary N in PhA9. The role of lipophilicity is more nuanced as there is an optimal level for activity. Tellingly, while most modifications (SAR2, SAR3, and SAR4) led to an increase in lipophilicity, not all benefited potency. In this regard, isopropyl replacement (SAR2) at the chiral carbon appears to be the most favored site for incorporating lipophilic elements.

### PhA2 displays enhanced antimycobacterial activity with low resistance among the shortlisted actives

We selected PhA1-3, PhA9, and PhA11-13 from Table 1 as representative potent compounds (“actives”) for further characterization of antimycobacterial activity and whole cell phenotypic screening (Table 2). MIC_50BCG_ values of these compounds range from 10 μM to 16 μM, which is 13–20 times lower than that of VP (210 μM) (Table 1).

**Table 2:** MIC_50_ against M. bovis BCG in different growth media, MICs against Mtb H37Rv and Mab complex, and mammalian cell cytotoxicities (IC_50_) of phenylalkylamine actives

Compound	MIC_50 BCG_ With Glycerol^a^	MIC_50 BCG_ Without Glycerol^a^	MIC_50_/MIC_90_ *Mtb* H37Rv^b^	MIC_50_*Mab abscessus* ATCC 19977^c^	MIC_50_*Mab massiliens*e CCUG 48898-T^c^	MIC_50_*Mab bolletii* CCUG 50184-T^c^	IC_50_ μM Vero E6^d^	IC_50_ μM HepG2^d^
**PhA1**	12	21	19/43	76/25	100/15	97/22	51	34
**PhA2**	10	22	17/25	54/19	61/11	62/16	28	29
**PhA3**	11	18	19/42	47/17	42/10	47/13	27	32
**PhA9**	11	22	–e	74/–e	123/–e	103/–e	15	15
**PhA11**	19	46	19/40	>250/175	>250/21	>250/160	57	44
**PhA12**	21	35	23/46	>250/194	>250/31	>250/72	69	51
**PhA13**	12	30	21/45	>250/250	>250/23	>250/1250	60	42
**VP**^f^	210	450	241/540	1290/1200	1100/1150	1450/1250	543	504
**CLR**^g^	–e	–e	–e	0.2/0.15	0.5/0.1	8.8/1.0	–e	–e

MIC values are cited as average of at least 2 separate determinations.

a MIC_50_ (μM, defined in Table 1) were determined on *M. bovis* BCG cultures (7H9 broth) with or without glycerol. Incubation period after compound addition was 5 days and determinations were made following a reported method.^26^

b MIC_50_, MIC_90_ (μM, defined in Table 1) were determined on *Mtb* H37Rv cultures (incubation period after compound addition was 5 days) following a reported method.^26^

c MIC_50_ values (uM) were separately obtained from 7H9 and CA-MHB media against the *Mab* subspecies. Incubation period after compound addition was 3 days. MIC_90_ values are given in Table S2.

d Concentration required to suppress growth of Vero E6 (African green monkey kidney epithelial cells) or immortalized hepatocellular carcinoma HepG2 cells by 50% compared to untreated controls. Determinations were made following a reported method.^26^

e Not determined.

f Verapamil (VP): MIC_50BCG_ 205 μg/mL (7H9 without glycerol), 96 μg/mL (7H9 with glycerol); MIC_50Mtb_ 110 μg/mL, MIC_90Mtb_ 246 μg/mL; MIC_50_ 500–564 μg/mL *Mab* complex.

g Positive control clarithromycin (CLR) for *Mab* complex. MIC_90_ 0.1–8.8μM (0.08–6.6 μg/mL). CLR breakpoint as defined by the Clinical and Laboratory Standards Institute is R > 8 μg/mL.^28^ By this criterion, all 3 subspecies are susceptible or moderately susceptible to CLR.

As glycerol in standard 7H9 growth media is known to artefactually increase inhibitory activity of antimycobacterial agents and lead to a decoupling of *in vitro* and *in vivo* activities,^29^ MIC_50BCG_ of the actives were redetermined in glycerol-free media. Higher MIC_50BCG_ values were observed in the absence of glycerol but the narrow ∼2-fold difference did not reasonably point to dependency.

Next, the actives were evaluated against pathogenic *Mtb* H37Rv. MIC_50Mtb_ values were broadly comparable (<2-fold difference) to *M. bovis* BCG values and fell within a narrow range of 17 μM–23 μM. MIC_90Mtb_ values showed a wider variation (25μM–46 μM). On both counts, PhA2 had the lowest MIC value and is deemed to have greater growth inhibitory activity than the other actives (PhA1, 3, 9, 11–13).

The actives were then tested on the NTM *Mycobacterium abscessus* (*Mab*) which comprises 3 subspecies as shown in Table 2.^30^ Here, MICs were dependent on the growth media, with lower MICs recorded in cation-adjusted Mueller Hinton broth (CA-MHB) as compared to 7H9 media. CA-MHB is recommended for drug susceptibility testing against NTM.^28^ We noted that PhA1-3 were consistently more potent than PhA11-13 in either media (Table 2). PhA2 and PhA3 also inhibited growth of seven *Mab* clinical isolates at lower concentrations (MIC_50_ 22–39 μM, 12-21μg/mL) than VP (900–1100 μM, 409-500μg/mL) (Table S3).

Confirmation of a validated TB hit compound requires a comparison of the cytotoxicity (IC_50_) of the test compound on mammalian Vero cells with its growth inhibitory MIC_50_ on mycobacteria.^31^ The selectivity index (SI = Vero IC_50_/mycobacterial MIC_50_) of a validated hit should exceed 10.^31^ Disappointingly, the selective indices (IC_50Vero_/MIC_50 BCG_) of our actives were <10. When assessed on another mammalian cell line (human cancer HepG2 cells), low selectivities were again detected. Among the actives, PhA9 had the least favorable selectivity index (SI = 1.4) and hence was precluded from subsequent mode of action experiments.

**Figure 2:**
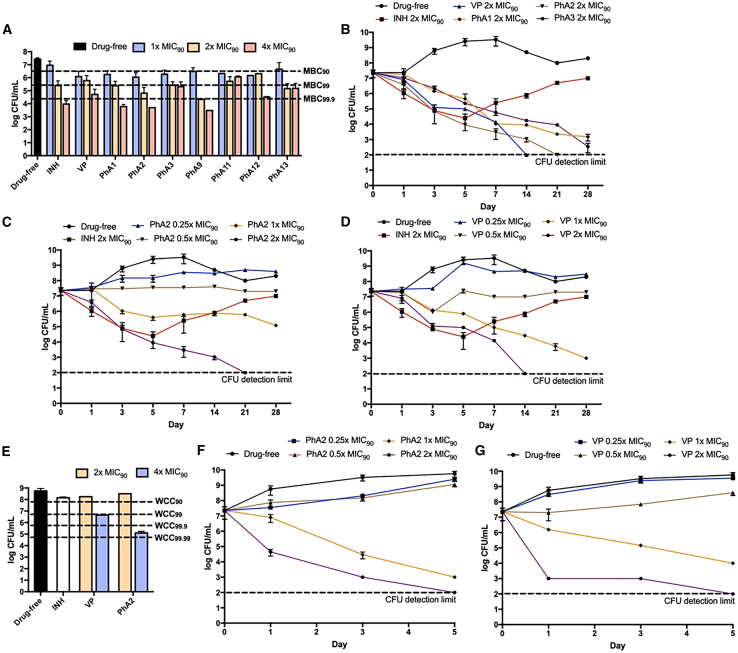
Bactericidal profiles of PhA2 and other phenylalkylamine actives in M. bovis BCG and Mab abscessus (A) Colony forming units (CFUs) of growing exponential phase *M. bovis* BCG cultures treated with actives (PhA1-3, PhA9, PhA11-13), VP and INH (control) at 1x, 2x, 4x MIC_90BCG_. Mid-log phase cultures adjusted to OD_600_ = 0.05 were treated with test compound (5 days, 37^o^C, 110 rpm), then plated on 7H10 agar plates followed by enumeration of CFUs. Minimum bactericidal concentrations to reduce CFUs by 10-, 10^2^-, 10^3^-fold are given by MBC_90_, MBC_99_, MBC_99.9_, respectively, compared to drug-free control at time zero. CFUs were averaged from at least 3 independent determinations. MIC_90BCG_ of INH and VP are 3.4 μM (0.45 μg/mL) and 550 μM (250 μg/mL) respectively. MIC_90BCG_ of test compounds are PhA1 (25 μM, 13 μg/mL), PhA2 (18 μM, 9.4 μg/mL), PhA3 and PhA9 (22 μM, 12 μg/mL), PhA11 (25 μM, 13 μg/mL), PhA12 (29 μM, 16 μg/mL), PhA13 (23 μM, 13 μg/mL). (B) Time-kill curves of growing *M. bovis* BCG cultures treated with PhA1, PhA2, PhA3 at 2xMIC_90 BCG_ (26, 19, 24 μg/mL respectively). Also tested at 2xMIC_90 BCG_ were isoniazid (INH,0.9 μg/mL) and VP (500 μg/mL). At each time point, aliquots were withdrawn, plated on agar and incubated for 21 days to determine CFUs. Time-kill curves of growing *M. bovis* BCG treated with (C) PhA2 and (D) VP at concentrations of 0.25x to 2x MIC_90_, equivalent to 2.4–19 μg/mL PhA2 and 63–500 μg/mL VP. INH (control) was assessed at 2x MIC_90_ (7 μM, 0.9 μg/mL). At each time point, aliquots were withdrawn, plated on agar and incubated for 21 days to determine CFUs. (E) Bactericidal activities of PhA2, VP and INH (control) under O_2_ deprived conditions (Wayne model). PhA2 was tested at 2x MIC_90_ (36 μM, 19 μg/mL), and 4x MIC_90_ (72 μM, 38 μg/mL) VP was tested at 2x MIC_90_ (1.1 mM, 500 μg/mL) and 4x MIC_90_ (2.2 mM, 1 mg/mL). INH was tested at 4xMIC_90_ (14 μM, 1.8 μg/mL). WCC_90_, WCC_99_, WCC_99.9_ are concentrations required to reduce CFUs of nonreplicating *M. bovis* BCG by 10-, 10^2^-, 10^3^-fold respectively after 5 days treatment compared to drug-free cultures at time zero. CFUs were averaged from at least 2 independent determinations. Time-kill curves of *Mab abscessus* treated with (F) PhA2 and (G) VP at concentrations of 0.25x to 2x MIC_90_ (PhA2: 11–90 μg/mL, VP: 275 μg/mL −2.2 mg/mL). At each time point, aliquots were withdrawn and incubated for 3 days to determine CFUs. MIC_90 abscessus_ of PhA2 and VP are 85 μM (45 μg/mL) and 2.4 mM (1.1 mg/mL). Except for (E), CFUs for other panels were determined from *n* = 3 separate runs and presented as mean ± SD (GraphPad Prism).

Next we assessed the minimum bactericidal concentrations (MBC) of the actives on exponential phase *M. bovis* BCG, following a reported method.^32^ As shown in Figure 2A, all the compounds reduced colony formation by 10-fold (MBC_90_) at 1x MIC_90_ but only PhA2 and PhA9 achieved 10^3^-fold kill (MBC_99.9_) at 2x MIC_90_. The other compounds either required higher concentrations (4x MIC_90_ for PhA1, PhA12) or failed to reach this level of kill altogether. While PhA2 and PhA9 were equally bactericidal, PhA9 was discounted because of its selectivity index.

We also determined the time kill profiles of PhA1-3 (at 2xMIC_90BCG_) against *M. bovis* BCG.^33^ At the 21-day time point, PhA2 reduced colony forming units (CFUs) by a striking 10^5^-fold, in effect reaching the limit of detection. On the other hand, cultures treated with PhA1 and PhA3 remained viable, with CFUs that were 10–100 times above the detection limit. Furthermore, PhA2 suppressed colony formation throughout the 21 days with no rebound in growth, unlike cultures treated with the control isoniazid (INH) where an uptick in CFUs was observed after 5 days (Figure 2B). The absence of a rebound suggests that PhA2 may suppress the emergence of resistant organisms. Interestingly, VP at 2XMIC_90BCG_ (1.1 mM, 500 μg/mL) displayed a similar time-kill profile as PhA2 but took a shorter time (14 days) to reduce the CFU count to the detection limit. Notwithstanding, this required a higher concentration of VP (1.1 mM, 500 μg/mL) as compared to PhA2 (36 μM, 19 μg/mL) although both were used at 2xMIC_90BCG._ We also evaluated time kill curves of PhA2 and VP over a range of concentrations, from 0.25x to 2xMIC_90 BCG_ (PhA: 2.4–19 μg/mL, VP: 63–500 μg/mL) and observed dose-dependent killing for both compounds (Figures 2C and 2D).

The bactericidal activity of PhA2 was assessed against *M. bovis* BCG cultures grown under low oxygen conditions (Wayne model) (Figure 2C).^34^^,^^35^ The hypoxic organisms resisted killing by PhA2 at 2x MIC_90BCG_ but succumbed to a 10^3^-fold kill at 4x MIC_90BCG_. In comparison, VP achieved only a 10^2^-fold kill at the same concentration. This finding is significant as low oxygen conditions are known to promulgate nonreplicating dormant subpopulations of drug-tolerant bacteria. Encouragingly, PhA2 exhibited an exceedingly low spontaneous mutation frequency of <10^−9^/CFU when incubated with *M. bovis* BCG for up to 8 weeks.

Finally, we assessed the dose-dependent time kill profiles of PhA2 against the rapid growing *Mab abscessus* (Figure 2F). There was a clear dose dependent trend and at the highest test concentration of 2xMIC_90_ (170 μM, 89 μg/mL), PhA2 reduced CFUs by 10^5^-fold which is the detection limit. As shown in Figure 2G, a similar time kill profile was observed for VP but at a significantly higher concentration of 4800 μM (2xMIC_90_, 2.2 mg/mL).

Taken together, PhA2 has the most favorable growth inhibitory profile among the actives based on its growth inhibitory MICs against *Mtb* H37Rv, ability to achieve 10^3^-fold kill of *M. bovis* BCG at 2xMIC_90BCG_, bactericidal activity in the Wayne model and time-kill profile. Hence it was selected for mode of action studies. Notwithstanding, we also assessed PhA1 and PhA3 alongside PhA2 in some experiments as described later.

### PhA2 disrupts mycobacterial membrane integrity

**Figure 3:**
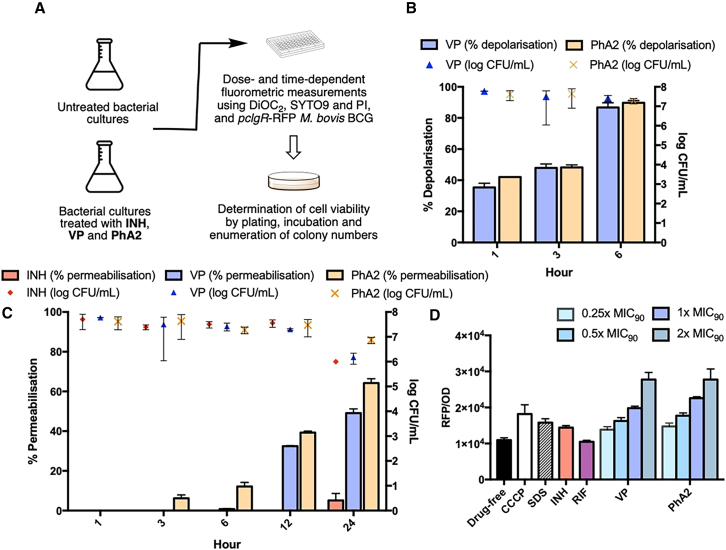
PhA2 disrupts membrane integrity in M. bovis BCG cultures (A) Schematic outline of *in vitro* assays employed to determine effects of PhA2 on mycobacterial membrane. (B) % Depolarization and viability (log CFU mL^−1^) of *M. bovis* BCG cultures treated with PhA2 and VP at 2x MIC_90_ (19 μg/mL PhA2, 500 μg/mL VP) over 6 h % Depolarization was determined from DiOC_2_ red/green ratios normalized against untreated cultures and cultures treated with CCCP.^36^ (C) Percentage permeabilization and viability of *M. bovis* BCG cultures treated with PhA2 and VP at 2x MIC_90_ (19 μg/mL PhA2, 500 μg/mL VP) over 24 h. Percentage permeabilized cultures were assessed from SYTO9/PI fluorescence, normalized against the same ratio from untreated cultures and cultures treated with SDS.^36^ (D) Dose dependent induction of *pclgR* promoter activity by PhA2 and VP in a recombinant strain of *M. bovis* BCG-*pclgR*-RFP. Concentrations used were 0.25x – 2x MIC_90_ (2.4–19 μg/mL PhA2, 63–500 μg/mL VP). Cultures were treated with controls and test compounds for 24 h before RFP fluorescence readings were taken and normalized against OD_600_ to account for loss in viability.^37^ Controls were CCCP (50 μM, 10 μg/mL), SDS (0.025%), INH (10 μM, 1.4 μg/mL), RF (10 μM, 8 μg/mL). (A–D): MIC_90BCG_ of PhA2, VP, INH, and RF are 18 μM (9 μg/mL), 550 μM (250 μg/mL), 3.4 μM (0.4 μg/mL), and 40 nM (33 ng/mL) respectively. Data are presented as mean ± SD (GraphPad Prism) from 3 separate determinations.

Cationic amphiphiles are reputed to disrupt membrane function.^9^^,^^10^ Indeed, the bactericidal activity of VP on *Mtb* cultures was attributed to perturbation of mycobacterial membrane energetics by the cationic amphiphilic phenylalkylamine scaffold.^14^ Given that PhA2 is a phenylalkylamine, we reasoned that it would also possess the membrane disruptive properties of VP. To investigate this hypothesis, we assessed PhA2 for its propensity to dissipate membrane electrical potential, alter membrane permeability and induce expression of cell envelope stress reporter genes, in *M. bovis* BCG cultures (Figure 3).

The membrane electrical potential is responsible for the polarized state of the bacterial membrane. It is one of two gradients contributing to the PMF which is essential for adenosine triphosphate (ATP) generation in mycobacteria. Here, we monitored the membrane potential of *M. bovis* BCG cultures using the fluorescent dye 3,3′diethyloxacarbocyanine iodide (DiOC_2_).^36^ Briefly, DiOC_2_ emits red fluorescence in mycobacteria with polarized membranes and green fluorescence in depolarized membranes. Hence, the red to green fluorescence ratio serves as an indirect measure of membrane polarization. Ratios were monitored in cultures treated separately with PhA2 and VP at 2x MIC_90BCG_ (19 μg/mL, 500 μg/mL, respectively) over 6 h and normalized against drug-free control and the positive control (carbonyl cyanide *m*-chlorophenyl hydrazine, CCCP) to give % polarization (Figure 3B). By the 6 h time point, depolarization was evident in PhA2-and VP-treated cultures. Importantly, these cultures were still viable, indicating that depolarization was not an epiphenomenon of cell death.

Next we examined the membrane permeabilizing effects of PhA2 and VP on *M. bovis* BCG cultures in the presence of SYTO9 and propidium iodide (PI).^36^ The cell permeant dye SYTO9 accumulates in both viable and dead cells and emits green fluorescence whereas the non-permeant PI only enters cells with compromised membranes and emits red fluorescence. Consequently, cultures with permeabilized membranes have lower green-red ratios as compared to cultures with intact membranes. Figure 3C depicts % permeabilization in cultures treated with PhA2 and VP at 2x MIC_90_ over 24 h. Both compounds induced membrane permeabilization with PhA2 eliciting a greater effect than VP. The negative control (INH) displayed negligible permeabilization. The permeabilization induced by PhA2 and VP was yet again observed in cultures that were viable.

clgR is a mycobacterial stress-sensing gene that is upregulated under conditions of cell envelope stress such as those encountered when the membrane is permeabilized or depolarized.^37^ Here, we monitored the responses of the *clgR* operon promoter to PhA2 and VP using a strain of *M. bovis* BCG where expression of red fluorescent protein (RFP) was controlled by the *pclgR* promoter.^26^ As predicated by their depolarizing and permeabilizing effects on mycobacteria, PhA2 and VP induced promoter activity in a dose dependent manner (Figure 3D). At the highest dose (2x MIC_90_), induction of clgR by both compounds exceeded that of the positive controls CCCP (depolarizer), INH (inhibitor of cell wall biosynthesis) and the surfactant sodium dodecyl sulfate (SDS, permeabilizer). No induction was observed in the presence of the rifampin (RIF, negative control).

Two other actives PhA1 and PhA3 were also evaluated alongside PhA2 in these assays and found to behave in a similar way (Figure S1). In all, we have shown that PhA2 induced membrane stress by perturbing the structural (permeabilization) and functional (depolarization) integrity of the mycobacterial membrane. Although these effects were elicited at a bactericidal concentration of PhA2 (2x MIC_90_, Figure 2A), they were observed early (1–6 h) in cultures that were viable.

### *In situ* imaging reveals localization of PhA2 in the mycobacterial cell membrane

**Figure 4:**
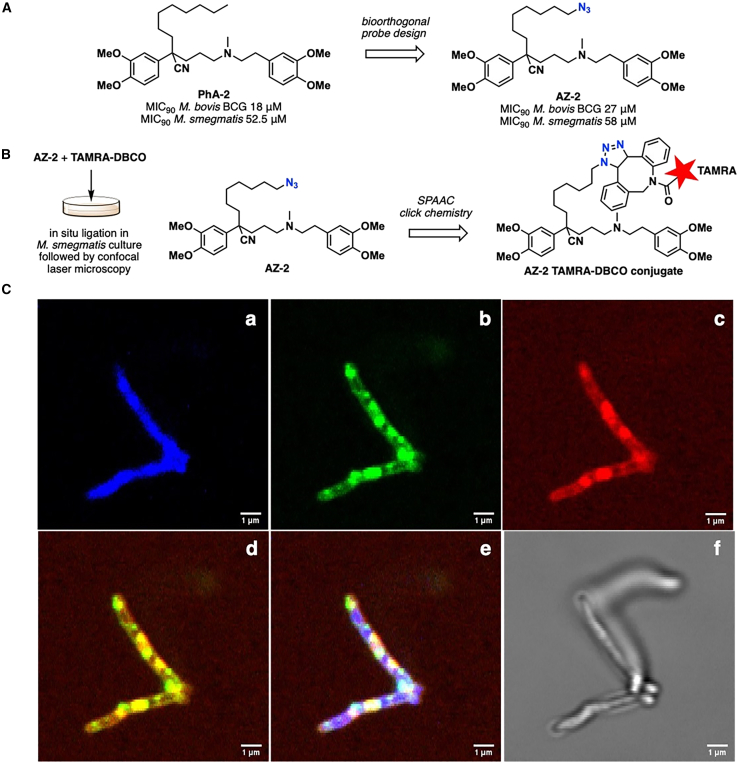
Live cell imaging of PhA2 using biorthogonal chemical probe AZ-2 in M. smegmatis (A) Structure of bioorthogonal chemical probe AZ-2 derived from PhA2 and corresponding MIC_90_ values in *M. bovis* BCG and *M. smegmatis*. MIC_90_ values are defined in Table 1 and were the average of 2 or more separate determinations. Synthesis and characterization of AZ-2 are given in Supplemental Information. (B) Copper-free ligation of AZ-2 with TAMRA-DBCO through SPACC for live-cell imaging in *M. smegmatis*. (C) Confocal microscopy images of *M. smegmatis* treated with (a) nuclear dye DAPI; (b) Control membrane dye FM1-43; (c) AZ-2 followed by TAMRA-DBCO; (d) Merged signals from (b) and (c); (e) Merged signals from (a), (b) and (c); (f) Image under bright field. Visualization of (b), (c) and (f) were carried out with TAMRA, FITC and brightfield channels, respectively. Scale bar: 1 μm. Figures are from one of 2 separate repeats.

A bioorthogonal chemical probe AZ-2, an azide analog of PhA2, was synthesized and used for live cell imaging in *M. smegmatis* following a reported method.^38^ MIC_90_ values of AZ-2 in *M. bovis* BCG and *M. smegmatis* were comparable to those of PhA2 (Figure 4A; Table S4), validating AZ-2 as a viable probe. The terminal azide in AZ-2 undergoes copper-free ligation with fluorophores functionalized with dibenzocyclooctyne (DBCO) through strain-promoted azide-alkyne cycloaddition (SPAAC, Figure 4A). In mycobacteria, AZ-2 reacts spontaneously with 5-carboxytetramethylrhodamine-dibenzocyclooctane (TAMRA-DBCO) at sites where it is localized which is posited to be the cell membrane (Figure 4B). Confocal laser microscopy was employed to detect the fluorescence emitted by the AZ-2 TAMRA-DBCO conjugate in *M. smegmatis.* and to compare its signal overlap with that of the positive control membrane dye FM1–43.^39^ Fluorescence emitted by FM1–43 (green) and the AZ-2 conjugate (red) are depicted in panels b and c of Figure 4C, respectively. Merging the images from these panels revealed overlapping green and red emissions (panel d) at the boundary and interior of the mycobacterium. This finding supports the notion that AZ-2 is localized within the membraneous structures of the cell wall (boundary) and vesicles (interior). Taken together, corroboratory evidence from biochemical, genetic, and imaging experiments support a mode of action based on membrane disruption by PhA2.

### PhA2 impairs electron transport, oxygen consumption, and ATP generation

The viability of mycobacteria is predicated on a fully energized membrane to sustain ATP production. The energy generated by electron flow through the electron transport chain (ETC) is harnessed by the proton gradient of the PMF to drive ATP synthesis via F_1_F_o_-ATP synthase. We reasoned that the membrane disruptive effects of PhA2 would impede electron transfer that requires an intact and functional membrane. To test our hypothesis, we interrogated the effects of PhA2 on the ETC of *M. bovis* BCG cultures in three different experiments, namely reduction of the tetrazolium dye 3-(4,5-dimethylthiazol-2-yl)-5-(3-carboxymethoxyphenyl)-2-(4-sulfophenyl)-2H-tetra-zolium (MTS), measurement of intracellular NADH/NAD+ ratio and oxygen uptake.

**Figure 5:**
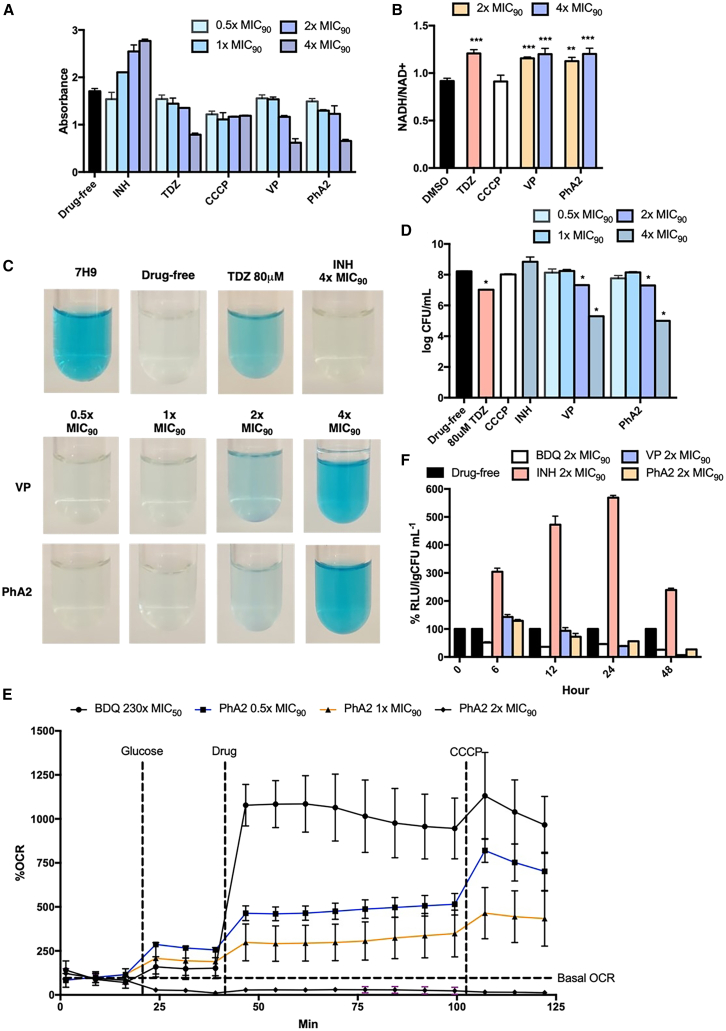
PhA2 disrupts electron transport, oxygen consumption, and ATP generation in M. bovis BCG cultures (A) Dose dependent reduction of MTS to formazan by *M. bovis* BCG cultures treated with PhA2, VP and controls INH, TDZ, and CCCP (30 min, 37^o^C).^40^ Compounds were tested at concentrations of 0.5x to 4x MIC_90BCG_. PhA2: 4.7–38 μg/mL, VP: 0.125–1 mg/mL, INH: 0.2–1.6μg/mL, TDZ: 5.5–44 μg/mL, CCCP: 5.1–40 μg/mL. Absorbance of formazan at 595 nm was read after 6 h. Mean and SD (GraphPad Prism) from *n* = 3 determinations. (B) NADH/NAD+ ratios of *M. bovis* BCG cultures treated with 2x–4x MIC_90BCG_ of PhA2 (19–38 μg/mL) and VP (0.5–1 mg/mL).^41^ Controls TDZ and CCCP were tested at 80 μM (30 μg/mL) and 50 μM (10 μg/mL) respectively. Experiments were carried out over 24 h at 37^o^C. Mean and SD (GraphPad Prism) from *n* = 3 determinations. ∗∗*p* ≤ 0.01 and ∗∗∗*p* ≤ 0.001 compared to DMSO control, Student’s t test, one-tail (Microsoft Excel). NADH, NAD+ levels were quantified using a commercial kit. (C) Oxygen consumption by *M. bovis* BCG cultures containing 0.001% methylene blue under O_2_ free conditions.^36^ Decolorization (active O_2_ uptake) was observed in drug-free 7H9 medium, cultures treated with VP at 0.5x (125 μg/mL), 1x MIC_90BCG_ (250 μg/mL), PhA2 at 0.5x (4.7 μg/mL), 1x MIC_90BCG_ (9.4 μg/mL) and INH at 4xMIC_90BCG_ (1.6 μg/mL) for 24 h at 37^o^C. No decolorization was observed in control 7H9 medium, cultures treated with PhA2 at 2x (19 μg/mL), 4x MIC_90BCG_ (38 μg/mL), VP at 2x (500 μg/mL), 4x MIC_90BCG_ (1 mg/mL), and TDZ (80 μM, 30 μg/mL) for 24 h at 37^o^C. Figures are from one of 2 separate repeats. (D) Viability of cultures (log CFU/mL) evaluated in (C) at the 24 h time point. Mean and SD (GraphPad Prism) from *n* = 3 determinations. ∗*p* ≤ 0.05 (Student’s t test, two-tail) compared to DMSO control (Microsoft Excel). (E) Oxygen consumption rate (OCR, expressed as % of basal level) of *M. bovis* BCG cultures treated with PhA2 at 0.5x (4.7 μg/mL) to 2x MIC_90BCG_ (19 μg/mL) and BDQ (230xMIC_50BCG_, 15 μM, 8.3 μg/mL).^42^ Glucose was added to nutrient deprived organisms followed by test compound and CCCP (to stimulate maximum O_2_ uptake). Figures are from one of 2 separate repeats. (F) ATP levels of *M. bovis* BCG cultures treated with the following at 2x MIC_90BCG_ over 48h: PhA2 (19 μg/mL), VP (500 μg/mL), BDQ (0.28 μg/mL), INH (0.8 μg/mL). ATP levels were measured with a luminescence based Promega BacTiter-Glo assay kit.^43^ Relative luminescence units (RLU) were normalized against log CFU/mL to account for changes in viability at each time point. Mean and SD (GraphPad Prism) from *n* = 3 determinations. (A–F): MIC_90BCG_ of PhA2, VP, BDQ, INH, TDZ, and CCCP are 18 μM (9 μg/mL), 550 μM (250 μg/mL), 0.25 μM (0.14 μg/mL), 3.4 μM (0.4 μg/mL), 30 μM (11 μg/mL) and 50 μM (10 μg/mL) respectively. MIC_50BCG_ of BDQ is 0.065 μM (36 ng/mL).

The reduction of MTS by NADH dehydrogenases to purple formazan is related to the efficiency of electron transport in the respiratory chain.^40^ Less formazan is formed when electron flow is impeded. Here, we monitored formazan formation in cultures treated with PhA2 and appropriate controls thioridazine (TDZ) and INH (Figure 5A). PhA2 caused a dose dependent reduction in formazan, similar to TDZ, an inhibitor of mycobacterial type II NADH dehydrogenase (NDH-2) (Figure 5A).^44^ PhA1 and PhA3 were also evaluated for reduction of MTS and found to behave in a similar manner (Figure S1). INH which was reported to activate the mycobacterial ETC, increased formazan formation.^45^

NADH is a major provider of electrons to the ETC via NADH dehydrogenases. As the ETC is the primary consumer of NADH, agents that intercept electron flow in the ETC would cause a buildup in NADH and increase the NADH/NAD+ ratio while those that promote electron flow would have the opposite effect.^46^ Here, we monitored levels of NADH and NAD+ in *M. bovis* BCG cultures treated with PhA2 using a NADH/NAD+ quantification kit.^41^ In keeping with our findings on MTS reduction, higher ratios were observed in cultures treated with PhA2 and TDZ (Figure 5B).

Diminished electron flow in the ETC would slow down the reduction of oxygen which is the final acceptor of electrons and hence impair respiration in mycobacteria. Here, we monitored oxygen consumption in *M. bovis* BCG cultures over 24 h using methylene blue as an oxygen indicator.^36^ Decolorization of methylene blue is indicative of oxygen uptake and this was observed in cultures treated with PhA2 at 0.5x (4.7 μg/mL) and 1x MIC_90_ (9.4 μg/mL, Figure 5C). At higher concentrations (2x, 4x MIC_90_ equivalent to 19 μg/mL, 38 μg/mL) of PhA2, oxygen uptake slowed ostensibly or ceased altogether as seen from the absence of decolorization. Notwithstanding, the diminished oxygen uptake was not due to cell death as colony counts of treated cultures, notably PhA2 at 2x MIC_90_ (19 μg/mL), remained high (Figure 5D). As for controls TDZ and INH, the results reiterated their effects on electron flow, namely continued oxygen uptake by INH which facilitates electron flow and diminished uptake by TDZ which has the opposite effect.

To confirm the findings from the methylene blue experiments, real time monitoring of oxygen consumption rate (OCR) in *M. bovis* BCG cultures treated with PhA2 were carried out on the Seahorse extracellular flux (XF) analyzer.^41^ Pleasingly, the results from the two experiments were in accord. Real time monitoring showed a dose dependent decline in oxygen uptake by PhA2 (Figure 5E). At 2x MIC_90_ (19 μg/mL), uptake was reduced to the basal level and did not increase in response to CCCP, implying marked suppression of the ETC. A sharp increase in OCR was elicited by a high dose of BDQ (15 μM or 8 μg/mL, equivalent to 230x MIC_50_) as reported by others.^42^

The synthesis of ATP is obligatorily coupled to the ETC^44^ and having shown that PhA2 impeded electron flow in the ETC, ATP levels were expected to fall in tandem. This was verified when we monitored ATP generation in *M. bovis* BCG cultures treated with PhA2 (2x MIC_90,_ 38 μg/mL) over 48 h using the Promega BacTiter-Glo assay, with the luminescence readout normalized against CFU at each time point (Figure 5F).^43^ PhA2 caused ATP levels to decline from 73% (12 h) to 27% (48 h). Similar responses were elicited in BDQ, PhA1 and PhA3-treated cultures whereas INH elicited a marked surge in ATP levels as reported by others.^43^^,^^46^ VP was investigated alongside PhA2 in the experiments depicted in Figures 5A–5F). Essentially, its effects on the ETC mirrored those of PhA2, reiterating its reported effects on mycobacterial membrane energetics.^14^

In all, our findings alluded to perturbation of membrane bioenergetics as causal to the bactericidal activity of PhA2. This is evident from the ability of PhA2 to induce membrane permeabilization and depolarization, activate the stress response *clgR* operon, impede electron flow in the ETC, diminish oxygen consumption and reduce intracellular ATP levels. That PhA1 and PhA3 demonstrated qualitatively similar effects as PhA2 on some of the assays hint at a possible scaffold effect, since these compounds are structurally cationic amphiphilic phenylalkylamines differing only in the side chain at the chiral carbon.

### PhA2 potentiates bactericidal properties of BDQ against *M. bovis* BCG and *Mab abscessus*

Dose response drug combination (checkerboard) experiments have shown that VP synergized with BDQ to cause a 20-fold decrease in BDQ MIC against drug sensitive and resistant strains of *Mtb* H37Rv.^14^ Combinations of bioenergetic inhibitors are known to accelerate clearance of mycobacteria^16^ and the synergistic interaction between VP and BDQ, both of which target membrane energetics, is a case in point. Viewed in this context, we posited that the interaction of PhA2 and BDQ on *M. bovis* BCG cultures would also be synergistic. Accordingly, the checkerboard assay was carried out to investigate the interactions of PhA2 with BDQ and representative TB drugs (INH, RIF, moxifloxacin, and kanamycin) which do not target mycobacterial bioenergetics. We found that only the interaction of PhA2 with BDQ was synergistic with a fractional inhibitory concentration index (FICI) of 0.5 which is indicative of synergism.^47^ In comparison, the other combinations had FICIs that point to indifferent interactions (FICI >0.5, Table S5). The assays were repeated on *Mab abscessus* and here again the PhA2-BDQ combination was synergistic (FICI 0.43) while the interactions of PhA2 with other anti-Mab drugs (clarithromycin, imipenem, and amikacin) were not (FICI >0.5, Table S7). As anticipated, the VP-BDQ combination was synergistic against *M. bovis* BCG and *Mab abscessus* (Tables S6 and S8).

**Figure 6:**
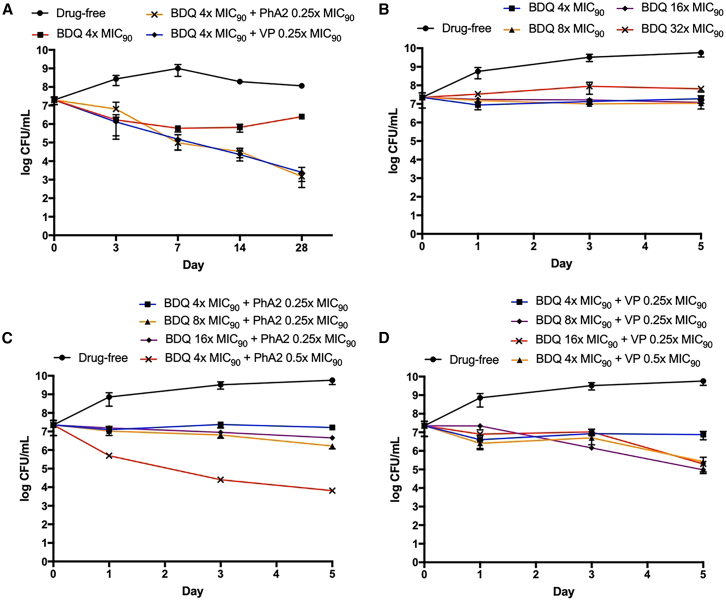
Time-kill profiles of BDQ/PhA2 and BDQ/VP combinations on M. bovis BCG and Mab abscessus cultures (A–D) Time- and dose-dependent killing of (A) *M. bovis* BCG by BDQ-PhA2 and BDQ-VP combinations.^32^ MIC_90BCG_ of BDQ, PhA2 and VP are 0.25 μM (0.14 μg/mL), 18 μM (9 μg/mL) and 550 μM (250 μg/mL) respectively; time- and dose-dependent killing of *Mab abscessus* by (B) BDQ at 4x to 32x MIC_90_ (0.84–6.7μg/mL), (C) BDQ-PhA2 combinations, and (D) BDQ-VP combinations. MIC_90abscessus_ of BDQ, PhA2, and VP are 0.37 μM (0.21 μg/mL), 85 μM (45 μg/mL), and 2.4 mM (1.1 mg/mL) respectively. Each experiment had 2 technical replicates and was performed 3 times independently. Mean ± SD values (GraphPad Prism) are shown in each panel.

The foregoing observations prompted us to ask if the bactericidal activity of BDQ would be augmented by supplementation with PhA2. To this end, we sought to identify the concentrations of BDQ and PhA2 which when combined would result in potentiation. Several sources have cited BDQ to be weakly bactericidal at 3x MIC_90_ against *Mtb*^48^^,^^49^ and this led us to assess the time kill profile of BDQ at 4x MIC_90BCG_ (0.56 μg/mL) on *M. bovis* BCG (Figure 6A).^32^ BDQ was indeed weakly bactericidal at this concentration, eliciting a modest 10-fold reduction in CFU at the 28-day time point. As for PhA2, we selected the lowest non-bactericidal concentration (0.25x MIC_90BCG_, 2.4 μg/mL) from its previously determined time-kill curve (Figure 2C). This combination of non- or weakly bactericidal concentrations of 4x MIC_90_ BDQ and 0.25x MIC_90_ PhA2 was then monitored for bactericidal activity over 28 days (Figure 6A). Gratifyingly, the combination displayed outstanding bactericidal activity as seen from the 10^4^-fold reduction in colony formation at the end of 28 days. The experiments were then repeated with the same composition of 4x MIC_90_ BDQ (0.56 μg/mL) and 0.25x MIC_90_ VP (63 μg/mL, lowest non bactericidal concentration from Figure 2D). The BDQ-VP combination was also bactericidal (Figure 6A), corroborating yet again the scaffold effect mentioned earlier for PhA1 and PhA3.

We then employed a similar approach to determine if PhA2 would enhance the bactericidal activity of BDQ against *Mab abscessus*. We first tested BDQ at concentrations of 4x to 32x MIC_90_ (0.84–6.7 μg/mL) but found none to be bactericidal (Figure 6B), as reported by others.^50^ Reviewing the time-kill curve of PhA2 (Figure 2F), we identified 0.25x (11 μg/mL) and 0.5x MIC_90_ (22 μg/mL) as non-bactericidal concentrations. Hence, we prepared four BDQ-PhA2 combinations for testing, of which three comprised BDQ at 4x, 8x, 16x MIC_90_ (0.84, 1.7, 3.4 μg/mL) and PhA2 at 0.25x MIC_90_ (11 μg/mL) respectively, and a 4^th^ combination of 4x MIC_90_ BDQ (0.84 μg/mL) and 0.5x MIC_90_ PhA2 (22 μg/mL). Interestingly, the first 3 combinations were weakly bactericidal and nearly equipotent despite the varying BDQ content (Figure 6C). In contrast, the 4^th^ combination with the lowest BDQ concentration (4x MIC_90_, 0.84 μg/mL) and a higher concentration of PhA2 (0.5x MIC_90_, 22 μg/mL) displayed strong bactericidal activity, achieving a significant 10^4^-fold reduction in CFU. Notionally these observations alluded to a pivotal role for PhA2 in influencing the bactericidal status of the combination.

A strikingly different profile emerged when we examined the bactericidal activities of the same four BDQ-VP combinations on *Mab abscessus* (Figure 6D). As with BDQ-PhA2, increasing the VP concentration from 0.25x MIC_90_ (275 μg/mL) to 0.5x MIC_90_ (550 μg/mL) in the combination with BDQ (4x MIC_90_, 0.84 μg/mL) resulted in bactericidal activity. However, unlike the BDQ-PhA2 combinations, the same level of kill was achieved when higher BDQ concentrations (8x, 16x MIC_90_ equivalent to 1.7 and 3.4 μg/mL) were used together with the lower VP concentration of 0.25x MIC_90_ (275 μg/mL). What this means is that starting from a non-bactericidal concentration of BDQ (4xMIC_90_) and VP (0.25 xMIC_90_), increasing either BDQ or VP led to bactericidal activity. Hence, we surmised that in BDQ-VP combinations, bactericidal activity was determined by BDQ or VP whereas in BDQ-PhA2 combinations, PhA2 played a decisive role.

## Discussion

We have gained two important insights from this investigation. The first pertains to the optimization of the phenylalkylamine template in VP for potent antimycobacterial activity through structure-activity guided modifications. Potent activity as displayed by the actives (PhA1-3, PhA9, and PhA11-13) required two structural features in the scaffold, namely (1) a flexible side chain of at least 8–10 carbon atoms, either at the chiral carbon or linking the basic nitrogen to the chiral carbon and (2) disubstitution of ring A with methoxy or chloro groups. Although these modifications led to significant lipophilicity gains (∼10^2^-fold based on clogP values), the weak statistical correlation (r^2^ = 0.36) between clogP and growth inhibitory MIC_90BCG_ suggest that while lipophilicity is undoubtedly necessary, it is not sufficient for potent activity. The long carbon side chain in the actives imparted rotatability and flexibility, features not normally deemed as drug-like as it would hamper passage across biological membranes.^51^ However, the implicit corollary of slower transition would be a longer exposure to membrane embedded targets and conceivably, greater perturbation. Furthermore, we noted that while all 70 synthesized compounds bore the cationic amphiphilic motif, only 7 were identified as actives. This modest outcome may reflect the wide array of structural features that fall within the remit of cationic amphiphilicity but are still in need of structural fine-tuning for optimal activity.

The second relates to the antimycobacterial profile of PhA2 and its synergistic interaction with BDQ. To our best knowledge, PhA2 is the most potent derivative of VP to be reported against mycobacteria. Its growth inhibitory activity exceeds that of VP by 30-fold on *M. bovis* BCG (based on MIC_90_), 22-fold on *Mtb* H37Rv (MIC_90_) and 63-fold on *Mab* abscessus (MIC_50_, CA-MHB). PhA2 displayed sustained bactericidal activity against *M. bovis* BCG cultures including those that were oxygen deprived, signaling its potential against drug tolerant non-growing organisms. We attributed these effects to the membrane-targeting potential of the cationic amphiphilic phenylalkylamine scaffold and the consequential lethal pleiotropic outcomes of membrane perturbation. This notion is corroborated by the qualitatively similar membrane responses elicited by PhA1, PhA3, and VP which have a shared phenylalkylamine scaffold. Unfortunately, PhA2 is disadvantaged in that its growth inhibitory activity is not adequately differentiated from its cytotoxicity on mammalian cells. However, this is circumvented when PhA2 is used in combination with BDQ with which it synergizes. PhA2 augmented the bactericidal activity of BDQ on *M. bovis* BCG at a concentration (0.25x MIC_90_ = 4.5 μM) which is well below its mammalian (Vero) cytotoxic IC_50_ values (28–29 μM). It also potentiates the bactericidal activity of BDQ on *Mab abscessus* at 0.5x MIC_90_ (43 μM, 0.11 μg/mL) but without the selectivity advantage. Notwithstanding, the BDQ-PhA2 combinations were strikingly bactericidal against both *M. bovis* BCG and *Mab abscessus*, affirming the merit of combining bioenergetic inhibitors to effect bacterial clearance.^16^ PhA2 permeabilize the mycobacterial cell membrane and disrupt several processes (PMF, ETC, and ATP synthesis) pivotal to mycobacterial bioenergetics. Tellingly, the permeabilizing and depolarizing effects of PhA2 were observed in cultures that were viable, reinforcing the notion that these events were not triggered by cell death but causal to it. Taken together, the activity profile of PhA2, its mode of action and synergistic interaction with BDQ justifies continued interest in the phenylalkylamine scaffold as a source of potential hits/leads for antimycobacterial drug discovery.

### Limitations of the study

First, the mechanistic investigations on PhA2 were largely confined to extracellular mycobacteria whereas these organisms typically reside within human macrophages. Our investigations showed that PhA2 disrupted membrane bioenergetics and respiratory function in mycobacteria but its effects on intracellular mycobacteria may be more nuanced. Hence additional experimentation is required to interrogate the intramacrophageal activity of PhA2 in order to establish *in vitro-in vivo* relevance.

Second, we have employed *M. bovis* BCG as an avirulent fast growing substitute of the pathogenic *Mtb* to facilitate screening and mechanistic investigations. Although the genomes of *M.bovis* BCG and *Mtb* share 99.95% identity at the nucleotide level, we are aware that they exhibit distinct phenotypes, virulence, and host tropism.^22^ Hence, the mechanistic responses elicited by PhA2 on *M.bovis* BCG should be confirmed on *Mtb*.

Lastly, PhA2 is investigated as a validated hit in this report and not as a preclinical candidate. To provide mechanistic insight, we have tested PhA2 and comparators (VP, BDQ) at high concentrations, which in the case of comparators exceeded their therapeutic plasma levels. For the same reason, we did not investigate PhA2 for any VP-related effects, in particular calcium channel blocking activity which forms the basis for the clinical use of VP in hypertension. Fortuitously, we found that PhA2 (a known compound^27^) decreased the contractility of cat papillary muscle, a proxy experimental model to assess blockade of calcium channels.^27^ Interestingly, PhA2 was significantly less potent than VP in this regard, with an ED_50_ (concentration required to reduce maximal contractility of the papillary muscle by 50%) of 130 μM versus 3.5 μM for VP. The authors attributed the weak contractile effects to the lipophilic *n*-octyl side chain at the chiral carbon of PhA2.^27^ We have shown here that replacing isopropyl with flexible lipophilic alkyl sidechains like *n*-octyl in PhA2 enhanced antimycobacterial activity, possibly by facilitating passage across the lipid rich matrix of the mycobacterial cell envelope. It would seem that fine-tuning lipophilicity of the scaffold at the chiral carbon is an effective means of optimizing antimycobacterial activity while advantageously minimizing calcium channel blockade. Further experimentation would be required to verify this claim.

Another VP-related effect that was not investigated is the inhibition of efflux pumps by VP and its analogs. Verapamil inhibits many transporter families, including mycobacterial efflux pumps.^52^^,^^53^^,^^54^ In addition to the insights from our mechanistic studies, which focus on disruption of mycobacterial bioenergetics, an alternative but not mutually exclusive narrative is that VP potentiates the activities of several anti-TB drugs, including bedaquiline, by directly inhibiting efflux pumps.^54^^,^^55^ This would result in higher intrabacterial drug concentrations and hence, a more pronounced bactericidal effect. However, it is noteworthy that the few studies that have monitored intracellular drug concentrations in *Mtb* could not detect significant-differences in drug accumulation by cultures treated with or without efflux pump inhibitors.^14^^,^^56^ The relative contribution of direct inhibition of transporter(s) versus interference with membrane integrity/bioenergetics to VP’s antibacterial mechanisms of action remains to be determined.

## Resource availability

### Lead contact

Further information and requests for resources and reagents should be directed to and will be fulfilled by the lead contact, Yulin Lam (chmlamyl@nus.edu.sg) upon reasonable request.

### Materials availability

All unique/stable and non-commercially available reagents generated in this study can be requested from the lead contact in reasonable amounts with a completed materials transfer agreement.

### Data and code availability

• Data availability: Confocal laser microscopy data reported in this paper will be shared by the lead contact upon request.
• Code availability: This paper does not report original code.
• Other items: Any additional information required to reanalyze the data reported in this paper is available from the lead contact upon request.

## Acknowledgments

This research is supported by the Republic of Singapore Ministry of Education (MoE) Academic Research Fund (grant no A-0008367-00-00) to Y.L. and the National Institute for Allergy and Infectious Diseases of NIH (United States of America, Grant no R01-Al132374) to T.D. The authors gratefully acknowledge Ministry of Education, Republic of Singapore for scholarship to Z.Y.P.

## Author contributions

Conceptualization, M.L.G., W.K.C., T.D., and Y.L.; methodology, Z.Y.P., M.L., and A.A.; investigation, Z.Y.P., M.L., A.A., C.C.S.C., K.J.G., M.Y.K.S., A.S.Y.N., and J.P.S.; writing—original draft, Z.Y.P., M.L., M.L.G., T.D., and Y.L.; writing—review and editing, Z.Y.P., M.L., M.L.G., T.D., and Y.L.; funding acquisition, M.L.G., T.D., and Y.L.; resources, A.A., M.L.G., W.K.C., B.C.G., T.D., and Y.L.; supervision, M.L.G., W.K.C., T.D., and Y.L.

## Declaration of interests

There is no conflict of interest to declare.

## STAR★Methods

### Key resources table

**Table:** 

REAGENT or RESOURCE	SOURCE	IDENTIFIER
**Bacterial and virus strains**		

*Mycobacterium tuberculosis* H37Rv	American Type Culture Collection (ATCC)	ATCC 27294
*Mycobacterium abscessus* subspecies *abscessus* clinical strain M9	Department of Laboratory Medicine, National University Hospital, Singapore	N/A
*Mycobacterium abscessus* subspecies *abscessus* clinical strain M111	Department of Laboratory Medicine, National University Hospital, Singapore	N/A
*Mycobacterium abscessus* subspecies *abscessus* clinical strain M232	Department of Laboratory Medicine, National University Hospital, Singapore	N/A
*Mycobacterium abscessus* subspecies *abscessus* clinical strain M337	Department of Laboratory Medicine, National University Hospital, Singapore	N/A
*Mycobacterium abscessus* subspecies *abscessus* clinical strain M404	Department of Laboratory Medicine, National University Hospital, Singapore	N/A
*Mycobacterium abscessus* subspecies *abscessus* clinical strain M422	Department of Laboratory Medicine, National University Hospital, Singapore	N/A
*Mycobacterium abscessus* subspecies *abscessus* clinical strain M506	Department of Laboratory Medicine, National University Hospital, Singapore	N/A

**Chemicals, peptides, and recombinant proteins**		

Verapamil	Sigma-Aldrich	Cat. No. 1711202
Bedaquiline	Sigma-Aldrich	Cat. No. SML4007
Isoniazid	Sigma-Aldrich	Cat. No. 1349706
Clarithromycin	Sigma-Aldrich	Cat. No. 1134379
Thioridazine	Sigma-Aldrich	Cat. No. 1662504
Rifampicin	Sigma-Aldrich	Cat. No. 557303
Moxifloxacin	Sigma-Aldrich	Cat. No. 1448606
Kanamycin	Sigma-Aldrich	Cat. No. K1377
FM1–43 (pyridinium 4-[2-[4-(dibutylamino)phenyl]ethenyl]-1-[3-(triethylammonio)propyl]-dibromide)	Thermofisher Scientific	Cat. No. T35356
DAPI (4ʹ,6-diamidino-2-phenylindole)	Thermofisher Scientific	Cat. No. D1306
Methylene blue	Thermofisher Scientific	Cat. No. 414240250
Glucose	Thermofisher Scientific	Cat. No. A16828.36
5-carboxytetramethylrhodamine-dibenzocyclooctane (TAMRA-DBCO)	Vector Laboratories	CCT-A131

**Critical commercial assays**		

CellTiter 96®AQueous One Solution Cell Proliferation	Promega	Cat. No. G3582
BacTiter-Glo™ Microbial Cell Viability Assay	Promega	Cat. No. G8230, 8231, 8232, 8233
BacLight™ Bacterial Membrane Potential Kit	ThermoFisher Scientific	Cat. No. B34950
LIVE/DEAD™ *Bac*Light™ Bacterial Viability Kit	ThermoFisher Scientific	Cat. No. L7012
NADH/NAD+ quantification kit	Sigma-Aldrich	Cat. No. MAK037

**Experimental models: Cell lines**		

Human hepatocellular carcinoma cells HepG2	ATCC	ATCC HB-8065
African green monkey kidney epithelial cells Vero E6	ATCC	ATCC CRL-1586

**Experimental models: Organisms/strains**		

*Mycobacterium bovis* Bacillus Calmette-Guérin (BCG) Pasteur	ATCC	ATCC 35734
*Mycobacterium smegmatis* mc^2^ 155	ATCC	ATCC 700084
*Mycobacterium abscessus* subspecies *abscessus*	ATCC	ATCC 19977
*Mycobacterium abscessus* subspecies *bolletii*	Culture Collection University of Goteborg (CCUG)	CCUG 50184-T
*Mycobacterium abscessus* subspecies *massiliense*	CCUG	CCUG 48898-T

**Software and algorithms**		

ChemDraw 20.1	PerkinElmer	N/A
Microsoft Excel	Microsoft	N/A
MestReNova 14.2.0	Mestrelab	N/A
GraphPad Prism Version 7.0	GraphPad	N/A
XeF96 Extracellular Flux Analyser software	Agilent Technologies	N/A

### Experimental model and study participant details

#### Bacterial strains and culture conditions

*M. tuberculosis* H37Rv (ATCC 27294), *M. bovis* Bacillus Calmette-Guérin (BCG) Pasteur (ATCC 35734), *M. smegmatis* mc^2^ 155 (ATCC 700084) and *M. abscessus* subsp. *abscessus* (ATCC 19977) were procured from the American Type Culture Collection (ATCC). *M. abscessus* subsp. *bolletii* (CCUG 50184-T) and *M. abscessus* subsp*. massiliense* (CCUG 48898-T) were procured from the Culture Collection University of Goteborg (CCUG). The other *M. abscessus* clinical isolates used for *in vitro* characterization (M9, M111, M199, M232, M337, M421, M422 and M506) were provided by Jeanette W.P. Teo (Department of Laboratory Medicine, National University Hospital, Singapore). *M. tuberculosis* H37Rv, *M. bovis* BCG and *M. smegmatis* strains were maintained in Middlebrook 7H9 broth (BD Difco) supplemented with 10% Middlebrook albumin-dextrose-catalase (ADC) (BD Difco), 0.5% (vol/vol) glycerol (Sigma Aldrich), and 0.05% (vol/vol) Tween 80 (Sigma Aldrich). All *M. abscessus* strains were cultured in either Middlebrook 7H9 broth medium supplemented with 0.2% glycerol, 10% ADC and 0.05% Tween 80 or Cation-adjusted Mueller-Hinton (camH) broth (BD Difco).

Wherever the growth of bacterial colonies on solid agar medium was required, all *M. bovis* BCG and *M. abscessus* strains were grown on Middlebrook 7H10 agar (BD Difco) supplemented with 0.5% glycerol and 10% (vol/vol) oleic acid-albumin-dextrose-catalase (OADC). Prior to running experiments, all strains were first grown in the respective broth medium at 37°C, with shaking on an orbital shaker set at 110 rpm. The strains were grown to exponential growth phase (mid-log phase) where the Optical Density at 600 nm (OD_600_) of the culture will be around 0.4–0.6. Middlebrook 7H9 broth base, 7H10 agar and its supplements were purchased from Beckton Dickinson, Difco, USA. Microtiter 96-well plates (3596, 3693) were purchased from Corning Inc., USA.

#### Human cell lines and culture conditions

Vero E6 (ATCC CRL-1586) cells, an African green monkey kidney cell line, and HepG2 (ATCC HB-8065) cells, a human hepatocellular carcinoma cell line, were maintained in Dulbecco’s Modified Eagle medium (DMEM, Gibco) at 37°C with 5% CO_2_ atmosphere. The media were supplemented with 10% heat-inactivated fetal calf serum (FCS, Gibco), 1% (vol/vol) penicillin and 1% (vol/vol) streptomycin. All reagents were obtained from Hyclone Laboratories, GE Healthcare, Buckinghamshire, UK.

### Method details

#### Microbiological

##### Minimum inhibitory concentration (MIC) determination^26^

The broth microdilution method was utilized to determine the MIC of the tested drugs or compounds. Each well of a transparent, flat-bottom 96-well Costar plate (Corning) was filled with 100 μL of complete 7H9 medium or cation-adjusted Mueller Hinton (camH) broth. Each drug or compound, dissolved in sterile distilled water or sterile 100% DMSO, was added to the wells to create the highest tested concentration. A 10-point 2-fold serial dilution of the compounds or drugs was then carried out. Mid-log phase cultures of the mycobacterial strain diluted to OD_600_ 0.1 using the same broth medium were then dispensed into the wells of the 96-well plate. 100 μL of diluted culture was used to inoculate each well of the 96-well plate (except the broth medium-only wells) to create a final OD_600_ of 0.05 in each well. The plates were then incubated at 37°C, under shaking on an orbital shaker set at 110 rpm. The incubation period was seven days for *M. tuberculosis* H37Rv, five days for *M. bovis* BCG, one day for *M. smegmatis* and three days for *M. abscessus*. To measure bacterial growth in each well at the end of the incubation period, the OD_600_ of the culture in each well was measured using the TECAN Infinite 200 PRO microplate reader (absorbance measurement at wavelength 600 nm). Percentage inhibition of growth in each well was calculated relative to growth in the drug-free wells. The MIC_90_ values were derived from these calculations.

##### Minimum bactericidal concentration (MBC) determination^32^

Following the procedure set out in MIC determination against exponentially growing *M. bovis* BCG, aliquots of serially diluted samples at 1x, 2x, and 4x MIC_90_ were plated on Middlebrook complete 7H10 agar plates, supplemented with 10% Middlebrook OADC enrichment and 0.5% glycerol, and incubated for at least three weeks at 37°C. Colonies were enumerated. MBC_90_ was defined as a concentration that reduced at least 1-log unit of CFU; MBC_99_ was defined as a concentration that reduced at least 2-log units of CFU; and MBC_99.9_ was defined as a concentration that reduced at least 3-log units of CFU in reference to the initial inoculum.

##### Time-kill kinetics assay^32^

A mid-log phase culture of *M. bovis* BCG or *M. abscessus* was diluted in complete 7H9 medium to achieve a cell density of 5 x 10^6^ CFU/mL. The diluted culture was split into individual T25 cell culture flasks (SPL Life Sciences) or 14 mL round-bottom tubes. Drugs or compounds were added to the culture. INH was utilized as the positive control for time-kill assays in *M. bovis* BCG. The culture bottles were then incubated at 37°C, under shaking on an orbital shaker set at 110 rpm. At each time-point of interest, 10 μL aliquots of each sample were serially diluted. 10 μL of neat and diluted samples were plated onto complete 7H10 agar plates. The plates were incubated at 37°C for at least three weeks for *M. bovis* BCG and three days for *M. abcessus*. At the end of the incubation period, the colonies that grew on the plates were counted. The CFU/mL of each sample was calculated from its respective colony number using the following formula: [(colony number) x (dilution factor)]/[volume plated (mL)]. Comparison of CFU/mL of the treated samples with that of the drug-free sample was done to evaluate the bactericidal activity of the compound or drug against *M. bovis* BCG or *Mab abscessus*.

##### Checkerboard titration assay^32^

The checkerboard assay was utilized to determine the type of interaction between phenylalkylamine compounds and anti-TB or anti-*Mab* drugs (all drugs purchased from Sigma Aldrich). Wells of a transparent, flat-bottom 96-well Costar plate (Corning) were filled with 100 μL of complete 7H9 medium. The phenylalkylamine compound was added to the vertical wells to achieve the highest concentration). The anti-TB or anti-*Mab* drug was added to the wells of horizontal wells to achieve their respective highest concentration. 2-fold serial dilutions were carried out for both tested compounds. The first well was drug-free and this was considered as the negative control. A mid-log phase culture of *M. bovis* BCG or *M. abscessus* was diluted to OD_600_ 0.1 using complete 7H9 medium. 100 μL of diluted culture was used to inoculate each well of the 96-well plate (except the 7H9 medium-only wells) to create a final OD_600_ of 0.05 in each. The plates were then incubated at 37°C, under shaking on an orbital shaker set at 110 rpm. To measure bacterial growth in each well at the end of the incubation period, the OD_600_ of the culture in each well was measured using the TECAN Infinite 200 PRO microplate reader. Percentage inhibition of growth in each well was calculated relative to growth in the drug-free well. To determine the type of interaction between the phenylalkyamine compounds with the tested drugs, the Fractional Inhibitory Concentration Index (FICI) was determined. FICI was calculated using the following formula: [(MIC of drug in combination/MIC of drug alone) + (MIC of VP or PhA2 in combination/MIC of VP or PhA2 alone)]. FICI ≤0.5 indicates synergy, FICI >0.5–4 indicates additivity (no interaction) and FICI >4 indicates antagonism between any 2 compounds.

##### Wayne bactericidal concentration (WCC) determination^32^

A mid-log phase culture of *M. bovis* BCG grown in Dubos broth media was diluted to OD_600_ of 0.005 and aliquoted into separate autoclaved glass tubes in 17mL each. One of the tubes was aliquoted pure Dubos media and another tube containing *M. bovis* BCG in Dubos media were added 51μL of 0.5mg/mL of methylene blue. The tubes were sealed tight using a septa and at least 3 layers of parafilm, then left to incubate at 37°C for more than 20 days. Starting on Day 7, one tube of culture was taken on alternate days to check for cell growth and had CFU enumerated. After the bacteria have been established to be at non-growing state, drugs or compounds were added to the culture using a syringe and a needle followed by incubation at 37°C for 5 more days. 10 μL of aliquots from the cultures were then plated and compared to that of the untreated for comparison.

##### Cytotoxicity assays in mammalian cells^26^

To each well in a 96-well plate, 50,000 cells in 100 μL media were seeded. Vero E6 and HepG2 cells were incubated overnight in DMEM for cell adherence at 37°C and 5% CO_2_. The media was removed from the wells and 100 μL solution made up of fresh DMEM media (99 μL) and the test compound (1 μL from a DMSO stock solution that was 22-fold more concentrated than the final concentration in the well) was then added to each well. DMSO content of each well was consistently kept at 0.5 %v/v. Treated plates were incubated for 48 h (5% CO_2_, 37°C), after which 10 μL of Celltitre 96 Aqueous One Solution was added to each well, without removal of media, and incubated for a further 3 h. Subsequently, absorbance readings were taken at 490 nm on a Tecan GENios Microplate Reader.

Cell viability was determined from the expression:

Cellviability(%)={[Absorbance(cells+cpd)−Absorbance(cpd)]/[Absorbance(cells+vc)−Absorbance(vc)]}×100%

where Absorbance (cells + cpd) = absorbance of wells containing cells and test compound in vehicle (media +0.5% DMSO); Absorbance (cpd) = absorbance of wells containing test compound in vehicle without cells (compound control); Absorbance (cells + vc) = absorbance of wells containing cells in vehicle (vc) only (untreated cells); Absorbance (vc) = absorbance of wells containing vehicle without cells (vehicle control). The percentage viability readings were plotted against log(concentration) on GraphPad Prism (Version 7.0, San Diego, CA) to give a sigmoidal curve, from which IC_50_, defined as the concentration of the compound that reduced cell viability by 50%, was obtained. At least 3 separate determinations were carried out.

##### Membrane stress induction^26^

Each well of a transparent, flat-bottom 96-well Costar plate (Corning) was filled with 100 μL of complete 7H9 medium. Each drug or compound, dissolved in sterile distilled water or sterile 100% DMSO, was added to the wells to create the highest tested concentration. A 10-point 2-fold serial dilution of the compounds or drugs was then carried out. Mid-log phase cultures of the fluorescent mycobacterial strain (*pclgR-mCherry*-BCG) grown in complete 7H9 media supplemented with 25 μg/mL of kanamycin was resuspended in fresh media to OD_600_ 0.4 and then dispensed into the wells of the 96-well plate. 100 μL of diluted culture was used to inoculate each well of the 96-well plate to create a final OD_600_ of 0.2 in each well. The plates were then incubated for 24 h at 37°C, under shaking on an orbital shaker set at 110 rpm. To measure fluorescence in each well at the end of the incubation period, the fluorescence signals at 587 nm and 630 nm of the culture in each well were measured using the TECAN Infinite 200 PRO microplate reader and normalized against OD_600_ and plotted against the tested concentrations.

##### *In situ* imaging in M. Smegmatis^38^

Exponentially-growing *M. smegmatis* was diluted to OD_600_ 0.1 in PBS with 0.05% Tween-80 and incubated with imaging probe AZ-2 for 3 h followed by addition of TAMRA-DBCO at 5 μg/mL. The bacterial cultures were kept at room temperature for 2 h in the dark and stained with 2.5μg/mL FM1–43 (pyridinium 4-[2-[4-(dibutylamino)phenyl]ethenyl]-1-[3-(triethylammonio)propyl]-dibromide) and 10μg/mL DAPI (4ʹ,6-diamidino-2-phenylindole. 10 μL of aliquot was used for imaging carried out using an Olympus IX71 inverted confocal microscope.

##### Membrane depolarization and permeabilization^36^

A mid-log phase *M. bovis* BCG culture was diluted to OD_600_ 0.1 using complete 7H9 medium and treated with DMSO, INH or the selected test compounds. The cultures were incubated at 37°C with orbital shaking of 110 rpm. At each time interval, 2 aliquots of 1.5 mL culture was taken and centrifuged to remove the supernatant. Resuspension of the cultures in PBS (for membrane potential measurements) or in 0.9% NaCl (for membrane permeabilization) was then carried out, followed by addition of DiOC_2_ or PI and SYTO9 respectively. The cultures were then incubated at 37°C for 25 min in the dark and spun down to remove the supernatant followed by resuspension in PBS or in 0.9% NaCl. Two aliquots of 200 μL were then taken for measurements of fluorescence using the TECAN Infinite 200 PRO microplate reader. CCCP was used as the positive control for membrane potential measurements while percentage of membrane permeabilization was determined with respect to that of SDS. INH was included for comparison.

##### Intracellular ATP detection^43^

The intracellular ATP content of mycobacterial cells was measured using a luminescence-based assay involving the BacTiter-Glo reagent (Promega). 25 μL of aliquot for treated *M. bovis* BCG cultures in each well of a white flat-bottom 96-well Costar plate was added 25 μL of the BacTiter-Glo reagent. BDQ was used as a positive control. Luminescent signals in each well were measured using the TECAN Infinite 200 PRO microplate reader and translated to the corresponding intracellular ATP levels using a standard calibration curve obtained.

##### Measurement of reductive potential of electron transport chain^40^

A mid-log phase *M. bovis* BCG culture was diluted to OD_600_ 0.4 using complete 7H9 medium and treated with test compounds, thioridazine (TDZ) as the positive control, INH as the negative control and CCCP for comparison in a 96-well Costar transparent plate. The plate was incubated at 37°C for 30min followed by addition of 25 μL of of Celltitre 96 Aqueous One Solution and kept at 37°C for 6 h. The absorbance readings were read using the TECAN Infinite 200 PRO microplate reader.

##### NADH/NAD+ ratio determination^41^

Actively-growing *M. bovis* BCG was diluted to an OD_600_ of 0.5 and 1 mL cultures were treated with the test compounds, CCCP (the negative control) or TDZ (the positive control). After treatment for 24 h at 37°C, the cultures were split into two 500μL aliquots for separate extraction of NADH and NAD+. Bacteria were pelleted by centrifugation and the supernatant was removed. 300 μL of 0.2 M HCl (for NAD+ extraction) or 0.2 M NaOH (for NADH extraction) was added and placed in a 55°C water bath for 10 min, followed by immediate cooling in ice. After cooling, sample pH was neutralized with 300 μL 0.1 M NaOH (for NAD+ extraction) or 0.1 M HCl (for NADH extraction). Precipitates were removed via centrifugation and 50 μL of supernatant was transferred to a clear-bottom, 96-well plate (Corning, USA). NADH or NAD+ was quantified using the NADH/NAD+ quantification kit from Sigma-Aldrich per the manufacturer’s instructions. Briefly, 100 μL of NAD+ cycling enzyme mix was added to the samples and incubated for 5 min at RT, under constant shaking to convert NAD+ to NADH. Thereafter, 10 μL of NADH developer was added to each well and incubated at RT until color developed. Endpoint absorbance at 450 nm was measured using the TECAN Infinite 200 PRO microplate reader and the NADH/NAD+ ratio was calculated.

##### Oxygen consumption inhibition assay using methylene blue^36^

A mid-log phase *M. bovis* BCG culture was diluted to OD_600_ 0.4 using complete 7H9 medium. 2 mL of the diluted culture was transferred to each 14 mL round bottom tube. DMSO, TDZ (Sigma Aldrich) and CCCP (Sigma Aldrich) were pipetted into their designated round bottom tubes and used as controls. Separate cultures were treated with VP and PhA2 at 0.5x, 1x, 2x and 4x MIC_90_ each. A drug-free culture tube was used as a positive control, while a 7H9 medium-only tube was used as a negative control. All the tubes were incubated at 37°C, under shaking at 160 rpm for one hour. After the incubation period, 0.001% methylene blue (Sigma Aldrich) was added to all tubes. The tubes were not fully capped and subsequently placed in the GasPak EZ Standard Incubation Container (BD), where 7 GasPak EZ anaerobe sachets (BD) were present to quench all oxygen in the container. Not fully capping the tubes ensured that oxygen within the tubes could also be quenched. The anaerobic container was incubated at 37°C, without agitation, for 24 h. At the end of the incubation period, the tubes were removed from the anaerobic container and pictures were taken of the tubes to record down the color of culture in each tube. To quantitatively assess the effect of the antibiotics on methylene blue decolourization, 200 μL of each sample was transferred to each well of a transparent, flat-bottom 96-well Costar plate. The absorbance at 665 nm of the methylene blue in the samples was measured using the TECAN Infinite Pro 200 plate reader. The absorbance levels were indicative of the level of intact methylene blue in the samples and hence, the level of inhibition of methylene blue decolourization. The levels of methylene blue decolourization of the untreated and antibiotic-treated samples were represented as a percentage relative to the 7H9 medium-only tube.

##### Measurement of oxygen consumption rate (OCR)^42^

The OCR of *M. bovis* BCG adhered to the bottom of a poly-D-lysine (PDL) coated XF cell culture microplate (Seahorse Biosciences), at 5 x 10^6^ bacilli per well, were measured using a XeF96 Extracellular Flux Analyser (Seahorse Biosciences). Assays were carried out in unbuffered 7H9 media (pH 7.4) without carbon source. *M. bovis* BCG bacilli were starved in liquid media, using 7H9 supplemented only with 0.05% Tween-80, for 24 h before being seeded into the XF cell culture microplate and the start of the experiment. In general, basal OCR was measured for 21 min before the automatic addition of 2 mg/mL glucose followed by either BDQ or the test compound (VP or PhA2) and then CCCP through the drug ports of the sensor cartridge.

##### Determination of spontaneous mutation frequency^32^

To carry out the assay, a mid-log phase culture of *M. bovis* BCG was concentrated, via centrifugation to a final cell concentration of 10^10^ CFU/mL or 10^9^ CFU/mL. 100 μL of both cell concentrates were plated on each complete 7H10 agar plate containing 2x, 4x or 8x MIC of VP or PhA2. This ensured that 10^9^ CFU or 10^8^ CFU were plated on each agar plate. The agar plates were incubated at 37°C for at least 8 weeks followed by enumeration of CFU.

#### Chemical synthesis

##### General information about the chemical synthesis and characterization of phenylalkylamines

Reagents for organic synthesis were purchased from Sigma Aldrich Chemical Co., Alfa Aesar or Tokyo Chemical Industry Co. Ltd unless otherwise stated and used without further purification. Reaction progress was monitored with thin-layer chromatography (TLC) on Merck silica gel 60 F254 pre-coated aluminum plates with visualization by UV light. Flash column chromatography was carried out with silica gel (Merck, 230–400 mesh). ^1^H and ^13^C NMR spectra were determined on Bruker Avance 500 (AV500) and Bruker Avance 400 (AV400) instruments at 298K and analyzed using MestReNova 14.2.0 software. Chemical shifts were reported in parts per million (ppm) on the δ scale using residual protio-solvent signals (^1^H NMR, deuterated chloroform (CDCl_3_) δ 7.26; ^13^C NMR, CDCl_3_ δ 77.16) as internal reference. Determination of nominal mass was performed on an AB SCIEX Qtrap 2000 mass spectrometer with electrospray ionization (ESI) run in positive mode. Reversed-phase high-performance liquid chromatography (HPLC) was employed to validate compound purity, carried out on a Shimadzu Nexera SR HPLC system, Zorbax Eclipse SB-C18-5 μM column (4.6 × 250 mm) at UV detection wavelengths of 285 nm. Mobile phases employed were 90% acetonitrile-water (25 mM ammonium formate) or 95% methanol-water (25 mM ammonium formate) with a run time of 15 min and flow rate of 1.2mL/min respectively (Figure S2).

The method described by Singh et al.^8^ was followed with some modifications.

General procedure for the synthesis of Int 1: At 0°C under nitrogen atmosphere, 2.0M n-BuLi in hexane (1.55mL, 3.1mmol) was added to a stirred solution of corresponding phenylacetonitrile (2.8mmol) dissolved in anhydrous THF (8mL). The reaction mixture was stirred at 0°C for 0.5h, thereafter the respective alkyl bromide (1.5 eq.) was added. Upon completion of reaction (TLC, 1 h), the reaction mixture was quenched with saturated NH_4_Cl (15mL) and extracted with EtOAc (3 × 30mL). The organic layer was dried with anhydrous MgSO_4_ and concentrated. The crude product was purified by column chromatography (90:10 Hexane:EtOAc).

General procedure for the synthesis of Int 2: At −78°C, under nitrogen atmosphere, 2.0M n-BuLi in hexane (1.7 eq.) was added to diisopropylamine (1 eq.) in anhydrous THF. After 15 min, Int 1 in anhydrous THF was added dropwise to the reaction mixture. The mixture was stirred at −78°C for 0.5h before the respective 1-bromo-*n*-chloroalkane (1.5 eq.) was added dropwise and stirred for another 20 min before it was warmed to room temperature. Upon completion of reaction (TLC, 1h), the reaction was quenched with saturated NH_4_Cl (15mL) and extracted with EtOAc (3 × 30mL). The organic layer was dried with anhydrous MgSO_4_ and concentrated. The crude product was purified by column chromatography (85:15 Hexane:EtOAc).

General procedure for the synthesis of phenylalkylamines A1, A2, PhA1–PhA8 and PhA11–PhA68: To a solution of Int 2 in anhydrous DMF, K_2_CO_3_ (5 eq.), KI (catalytic amt.) and the corresponding amine (2 eq.) were added and the reaction mixture was stirred at 80°C for 12h. Upon completion of reaction (monitored by TLC), deionized water was added to the reaction mixture before extraction with EtOAc (3 × 30 mL). The organic layer was washed with brine, dried with anhydrous MgSO_4_ and concentrated. The crude product was purified by column chromatography and preparative TLC (98:2 CH_2_Cl_2_:MeOH with a small amount of Et_3_N).

General procedure for the synthesis of phenylalkylamines PhA69 and PhA70: To a solution of VP in anhydrous DMF, lithium aluminum hydride (2 eq.) was added followed by heating under reflux. The crude product was purified by column chromatography and preparative TLC (98:2 CH_2_Cl_2_:MeOH with a small amount of Et_3_N) to afford PhA69. PhA70 was synthesized by reacting a solution of PhA69 with 2-thiophene carbonyl chloride in the presence of Et_3_N in anhydrous DCM (Figure S3).

The method described by Singh et al.^8^ was followed with some modifications.

General procedure for the synthesis of Int 2 for compounds PhA9 and PhA10: Diisopropylamine (1.5 equiv) was added to anhydrous THF (5mL) and stirred at −78°C in a 25mL round bottom flask. n-BuLi in hexanes (1.5 equiv) was then added dropwise using a syringe and the reaction was allowed to stir for 10 min. Int 1 (250mg) was then dissolved in anhydrous THF (5mL) and added dropwise into the reaction mixture. After stirring for another 10 min at −78°C, 1-bromo-*n*-hydroxyl alkane (*n* = 10 or 12, 1.5 equiv) was added dropwise into the reaction mixture and stirred. After completion of reaction (TLC, 1h) the reaction was quenched with NH_4_Cl (10mL), extracted with ethyl acetate and washed with water (3 x 50mL). The organic layer was then filter dried with Na_2_SO_4_ and evaporated at reduced pressure. Subsequent purification was carried out by column chromatography.

General procedure for the synthesis of Int 3 for compounds PhA9 and PhA10: 50 mg of Int 2 was added to imidazole (3 equiv) in a 25mL round bottom flask. PPh_3_ (1.2 equiv) and I_2_ (1.5 equiv) was then added and the flask was allowed to stir for 12 h at room temperature. After the completion of the reaction (TLC), the reaction mixture was extracted with EtOAc and washed with water (3 x 15mL). The organic layer was then filter dried with Na_2_SO_4_ and evaporated at reduced pressure. Subsequent purification was carried out by column chromatography.

General procedure for the synthesis of phenylalkylamines PhA9 and PhA10: To a solution of Int 3 in anhydrous DMF, K_2_CO_3_ (5 eq.), KI (catalytic amt.) and the corresponding amine (2 eq.) were added and the reaction mixture was stirred at 80°C for 12h. Upon completion of reaction (monitored by TLC), deionized water was added to the reaction mixture before extraction with EtOAc (3 × 30 mL). The organic layer was washed with brine, dried with anhydrous MgSO_4_ and concentrated. The crude product was purified by column chromatography and preparative TLC (98:2 CH_2_Cl_2_:MeOH with a small amount of Et_3_N) (Figure S4).

Briefly, n-BuLi was used to deprotonate 3,4-dimethoxyphenylacetonitrile which was then reacted with 1-bromo-7-chloroheptane in anhydrous THF in an ice bath to form Int 4 in 43% yield. Thereafter Int 4 was stirred with sodium azide in anhydrous DMF at 80°C to afford Int 5 in 68% yield. LDA was made *in situ* and used to deprotonate Int 5, followed by a reaction with 1-bromo-3-chloropropane to afford Int 6 in 87% yield. Int 6 was subsequently treated with 2-(3,4-dimethoxyphenyl)-N-methylethylamine and K_2_CO_3_ in the presence of KI in anhydrous DMF at 80°C to give AZ-2 in 24% yield.

### Quantification and statistical analysis

Details of statistical analyses are given in Figure captions.

Figures 2, 3, 5, and 6: Mean ± SD were generated by GraphPad Prism from *n* = 3 determinations.

Figures 5B and 5D: Student ‘s *t-*tests were carried out on Microsoft Excel, *n* = 3 determinations.
