# Supplementary material for: A potent phenylalkylamine disrupts mycobacterial membrane bioenergetics and augments bactericidal activity of bedaquiline
Source: iScience. 2025 Jun 18;28(7):112915. doi: 10.1016/j.isci.2025.112915 (PMC12281142; doi:10.1016/j.isci.2025.112915)
Supplement: Document S1. Figures S1–S4 and Tables S1–S8, and Data S1 [file mmc1.pdf]

iScience, Volume *28*

Supplemental information

A potent phenylalkylamine disrupts mycobacterial membrane bioenergetics and augments bactericidal activity of bedaquiline

Zheng Yen Phua, Ming Li, Azhar Ali, Cedric Cheng Sheng Cheong, Kai Jie Goh, Marcus Yi Kang Seto, Amos Shi Ying Ng, Jickky Palmae Sarathy, Boon Cher Goh, Mei Lin Go, Wai Keung Chui, Thomas Dick, and Yulin Lam

Document S1. Figures S1 – S4

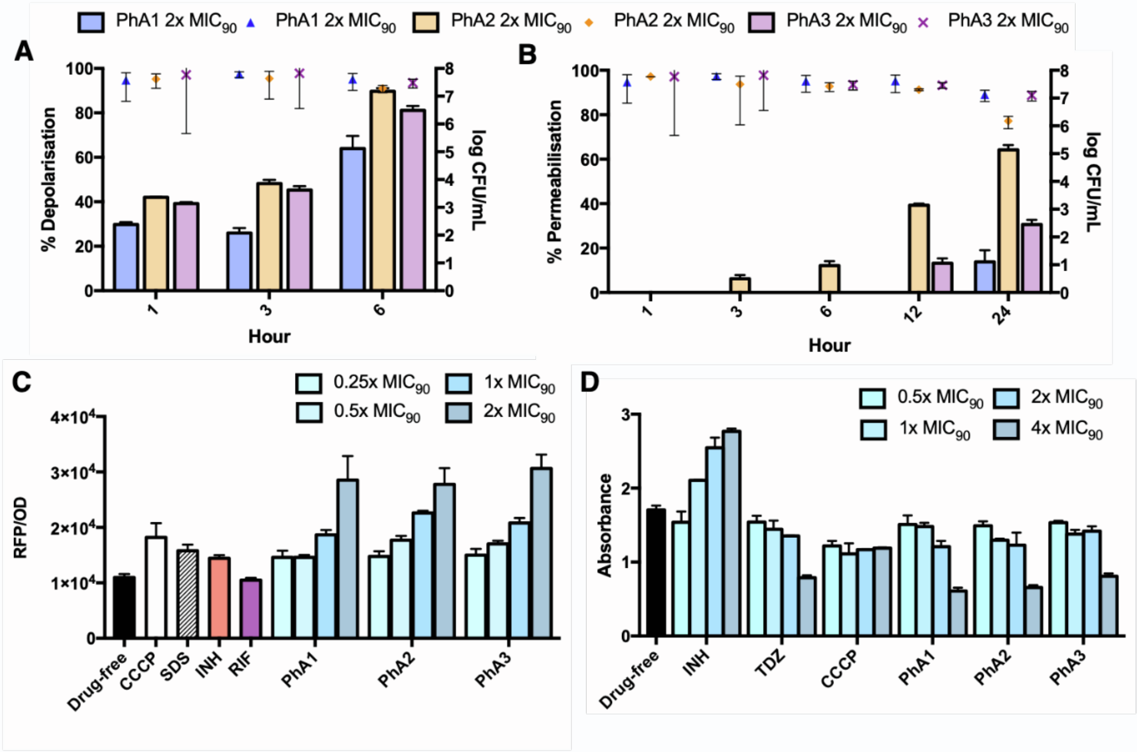

Figure S1. Membrane and MTS reduction assays carried out on PhA1–3 in *M. bovis* BCG, related to Figure 3

(A) % Depolarization and viability (log CFU mL^{-1}) of *M. bovis* BCG cultures treated with PhA1–3 at 2x $MIC_{90\ BCG}$ (PhA1 = 26 μg/mL, PhA2 = 19 μg/mL, PhA3 = 24 μg/mL) over 6 hours. % Depolarization was determined from $DiOC_2$ red/green ratios normalized against untreated cultures and cultures treated with CCCP.[36]

(B) % Permeabilization and viability of *M. bovis* BCG cultures treated with PhA1–3 at 2x $MIC_{90\ BCG}$ (PhA1 = 26 μg/mL, PhA2 = 19 μg/mL, PhA3 = 24 μg/mL) over 24 h. % Permeabilized cultures were assessed from SYTO9/PI fluorescence, normalized against the same ratio from untreated cultures and cultures treated with SDS.[36]

(C) Dose-dependent induction of *pclgR* promoter activity by PhA1–3 in a recombinant strain of *M. bovis* BCG-*pclgR*-RFP. Concentrations used were 0.25x to 2x $MIC_{90\ BCG}$ (PhA1 = 3.3–26 μg/mL, PhA2 = 2.4–19 μg/mL, PhA3 = 3–24 μg/mL). Cultures were treated with controls and test compounds for 24 h before RFP fluorescence readings were taken and normalized against OD_{600} to account for loss in viability.[37] Controls were CCCP (50 μM, 10 μg/mL), SDS (0.025%), INH (10 μM, 1.4 μg/mL), RIF (10 μM, 8 μg/mL).

(D) Dose dependent reduction of MTS to formazan by *M. bovis* BCG cultures treated with PhA1–3 and controls INH, TDZ and CCCP (30 min, 37 °C).[40] Compounds were tested at concentrations of 0.5x to 4x $MIC_{90\ BCG}$. PhA1 = 6.5–52 μg/mL, PhA2 = 4.7–38 μg/mL, PhA3 = 6–48 μg/mL, INH = 0.2–1.6 μg/mL, TDZ = 5.5–44 μg/mL, CCCP = 5.1–40 μg/mL. Absorbance of formazan at 595 nm were read after 6 h. Average of 3 independent determinations.

(A) to (D): $MIC_{90\ BCG}$ of INH, PhA1, PhA2, PhA3 and RIF are 3.4 μM (0.4 μg/mL), 25 μM (13 μg/mL), 18 μM (9.4 μg/mL), 22 μM (12 μg/mL) and 40 nM (33 ng/mL) respectively.

Figure S2: Chemical syntheses of phenylalkylamines A1, A2, PhA1–PhA8 and PhA11–PhA70

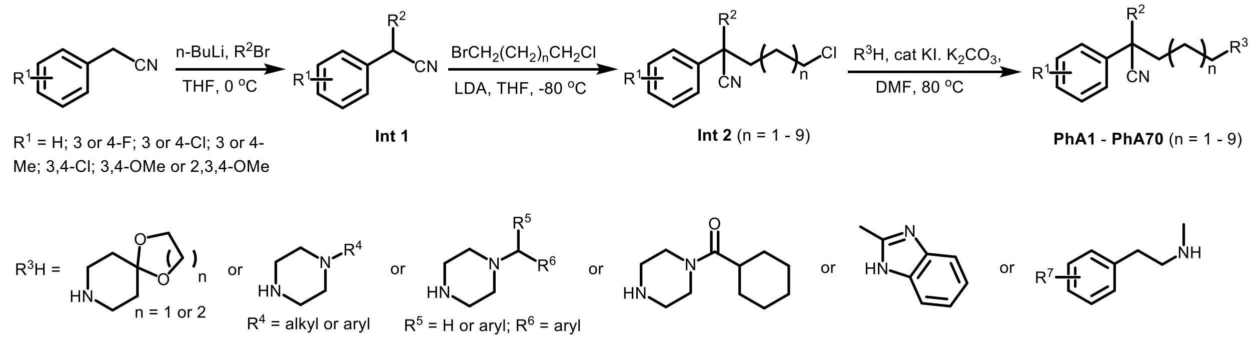

Details as described by Singh et al.[16] with some modifications.

Figure S3: Chemical syntheses of phenylalkylamines PhA9 and PhA10

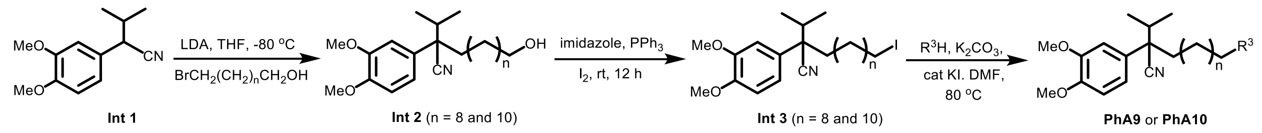

Details as described by Singh et al.[16] with some modifications.

Figure S4: Chemical synthesis of AZ-2

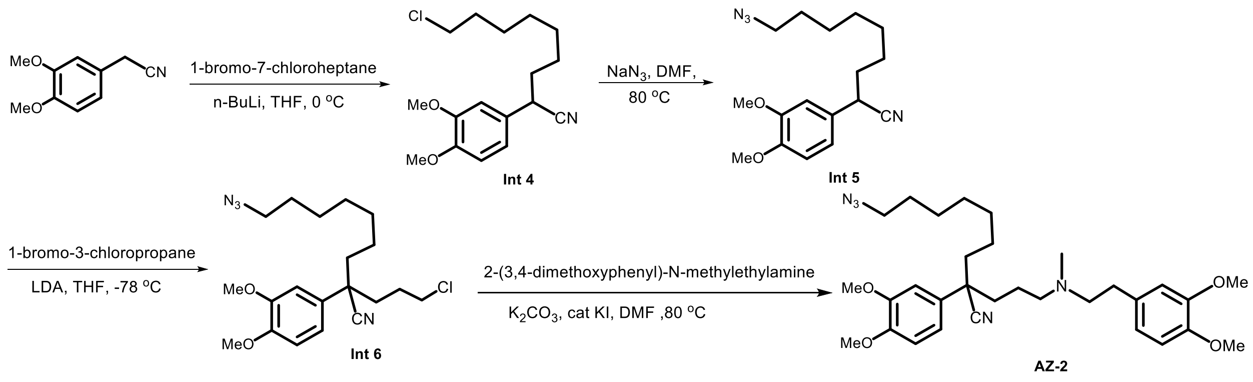

Document S2. Tables S1–S8

Table S1. Structures, MIC_{90} and clogP values of VP and all synthesized phenylalkylamines[a], related to Table 1

Compound	Structure	MIC_{90}[b] (μM)	clogP[c]	Compound	Structure	MIC_{90}[b] (μM)	clogP[c]
**VP**	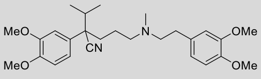	550	5.69	**A1**	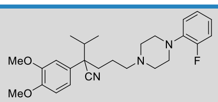	138	5.92
**A2**	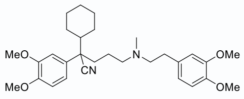	118	6.44	**PhA1**	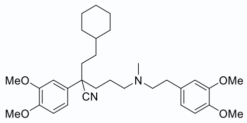	25	7.28
**PhA2**	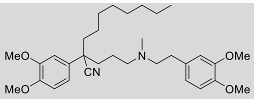	18	7.86	**PhA3**	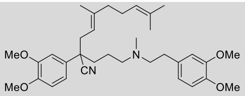	22	7.58
**PhA4**	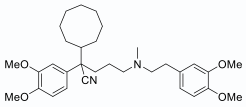	44	7.28	**PhA5**	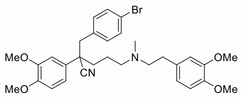	43	7.38
**PhA6**	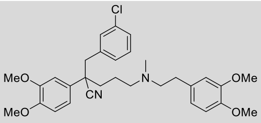	45	7.11	**PhA7**	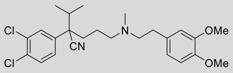	47	7.06
**PhA8**	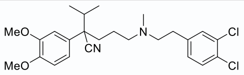	45	7.06	**PhA9**	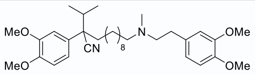	22	8.61
**PhA10**	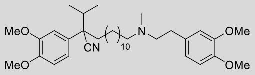	32	9.45	**PhA11**	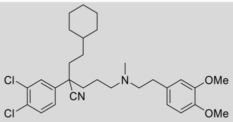	25	8.65
**PhA12**	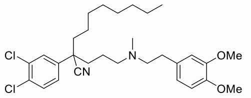	29	9.23	**PhA13**	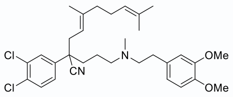	23	9.35
**PhA14**	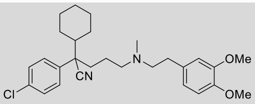	35	7.26	**PhA15**	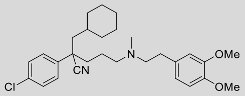	37	7.67
**PhA16**	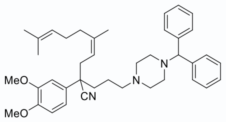	46	9.02	**PhA17**	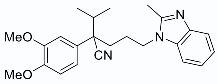	>250	5.81
**PhA18**	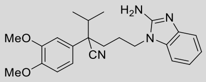	155	5.49	**PhA19**	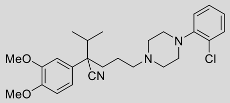	73	6.32
**PhA20**	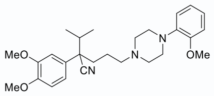	163	5.64	**PhA21**	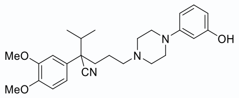	>250	5.37

**PhA22**	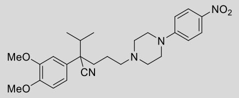	>250	5.37	**PhA23**	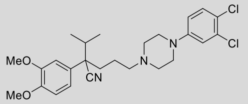	>250	6.88
**PhA24**	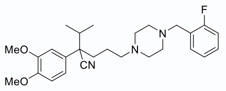	134	5.58	**PhA25**	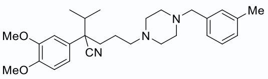	109	5.91
**PhA26**	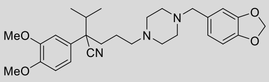	147	5.2	**PhA27**	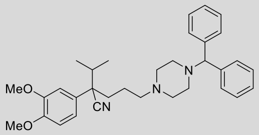	70	7.13
**PhA28**	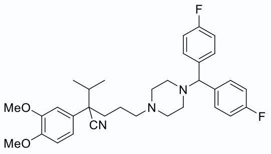	186	7.45	**PhA29**	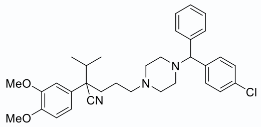	143	7.69
**PhA30**	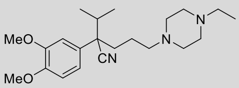	>250	4.03	**PhA31**	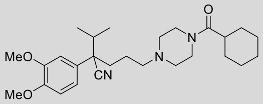	146	4.93
**PhA32**	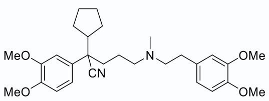	190	6.03	**PhA33**	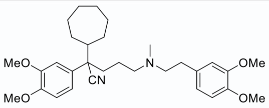	91	6.86
**PhA34**	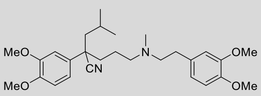	220	6.11	**PhA35**	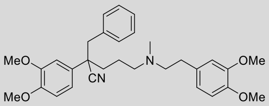	234	6.55
**PhA36**	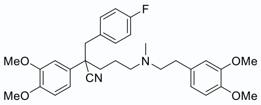	193	6.71	**PhA37**	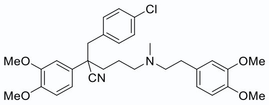	52	7.11
**PhA38**	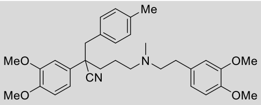	70	7.04	**PhA39**	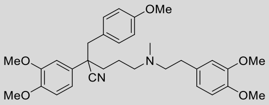	206	6.42
**PhA40**	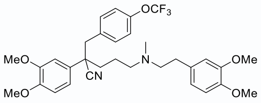	58	8.08	**PhA41**	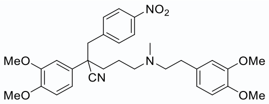	>250	5.11
**PhA42**	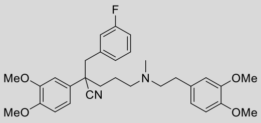	129	6.71	**PhA43**	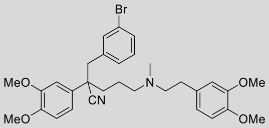	55	7.38
**PhA44**	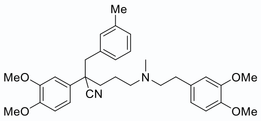	70	7.04	**PhA45**	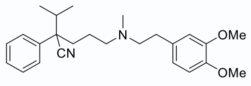	161	5.94
**PhA46**	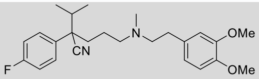	146	6.1	**PhA47**	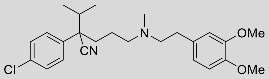	60	6.5
**PhA48**	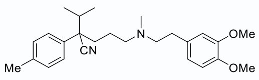	109	6.43	**PhA49**	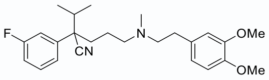	225	6.1
**PhA50**	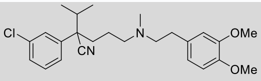	66	6.5	**PhA51**	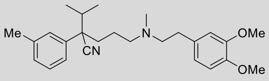	67	6.43

**PhA52**	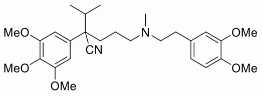	>250	5.56	**PhA53**	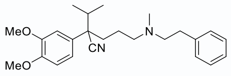	227	7.36
**PhA54**	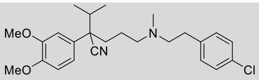	105	6.5	**PhA55**	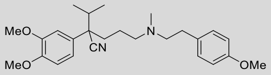	229	5.82
**PhA56**	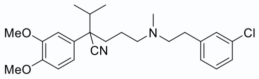	112	6.5	**PhA57**	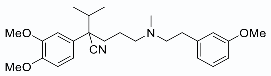	180	5.82
**PhA58**	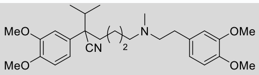	>250	6.11	**PhA59**	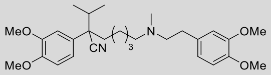	240	6.53
**PhA60**	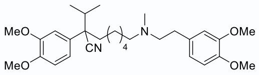	130	6.94	**PhA61**	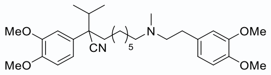	65	7.36
**PhA62**	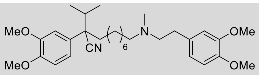	50	7.78	**PhA63**	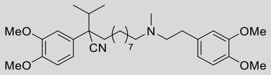	51	8.19
**PhA64**	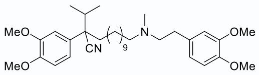	53	9.03	**PhA65**	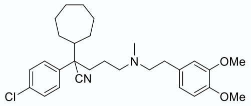	51	7.67
**PhA66**	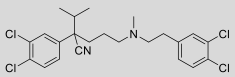	120	8.43	**PhA67**	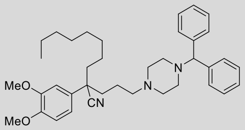	208	9.31
**PhA68**	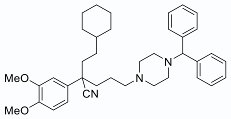	125	8.72	**PhA69**	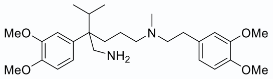	>250	4.7
**PhA70**	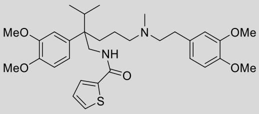	>250	5.22	**INH**	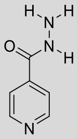	3.5	3.85

[a] PhA2, PhA52 and PhA57 are known compounds. PhA2 was reported by Mannhold et al.[27] PhA52 and PhA57 are FDA-approved VP derivatives, gallopamil and devapamil respectively.
[b] Minimum inhibitory concentrations required to reduce growth by 90% compared to untreated *M. bovis* BCG cultures cultures. Average of at least 2 separate determinations using the broth dilution method.
[c] clogP values were obtained from ChemDraw version 20.1.

Table S2. MIC_{90} of VP and 7 phenylalkylamines against all three subspecies of *Mab* complex in 7H9 and camH broth, related to Table 2

Compound	*M. abscessus* (*Mab*) complex MIC_{90} in 7H9/camH broth (μM)[a,b]		
	Mab abscessus ATCC 19977	*Mab massiliense* CCUG 48898-T	*Mab bolletii* CCUG 50184-T
**PhA1**	115/54	106/125	109/61
**PhA2**	85/26	91/28	95/27
**PhA3**	104/31	96/56	98/26
**PhA9**	190/-[c]	>200/-[c]	200/-[c]
**PhA11**	>250/>250	>250/>250	>250/>250
**PhA12**	>250/>250	>250/>250	>250/>250
**PhA13**	>250/>250	>250/>250	>250/>250
**VP**	2400/>2500	2250/>2500	2200/>2500
**CLR**[d]	0.5/0.4	2/0.23	19/11

[a] Minimum inhibitory concentrations required to reduce growth by 90% (MIC_{90}) compared to untreated cultures. Average of at least 2 separate determinations using the broth dilution method on *Mab* cultures.
[b] Separately determined in 7H9 and camH broth.
[c] Not determined.
[d] Positive control clarithromycin (CLR) for *Mab* complex.

Table S3. MIC_{50} of VP and PhA2 against seven clinical isolates of *Mab* in 7H9 broth

Compound	M9 (μM)	M111 (μM)	M232 (μM)	M337 (μM)	M404 (μM)	M422 (μM)	M506 (μM)
**VP**	1000	950	1080	900	990	1000	1010
**PhA2**	26.5	24	22	25	26.5	23	23.5
**CLR**[a]	0.1	0.08	0.27	0.15	0.1	0.22	0.09

[a] Positive control clarithromycin (CLR) for *Mab* complex.

Table S4. Structures and MIC_{90} of PhA2 and biorthogonal chemical probe AZ-2 against *M. bovis* BCG and *M. smegmatis* mc^{2}155, related to Figure 4

Compound	Structure	*M. bovis* BCG (μM)[a]		*M. smegmatis* mc^{2}155 (μM)[a,b]	
		MIC_{50}	MIC_{90}	MIC_{50}	MIC_{90}
**PhA2**	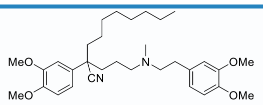	10	18	41	58
**AZ-2**	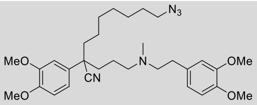	12	27	38	58

[a] Minimum inhibitory concentrations required to reduce growth by 50% (MIC_{50}) and 90% (MIC_{90}) compared to untreated mycobacterial cultures. Average of at least 2 separate determinations using the broth dilution method. Incubation period after compound addition was 1 day (*M. smegmatis* mc^{2}155).
[b] *M. smegmatis* mc^{2}155 is a non-pathogenic mycobacterial model organism.

Table S5. Fractional inhibitory concentration indices (FICI) of drug combination with PhA2 as determined in *M. bovis* BCG cultures

Combination (TB drug + PhA2)	Primary antimycobacterial target of TB drug	FICI[a]	Effect
Isoniazid + PhA2	NADH-dependent enoyl acyl carrier protein reductase (InhA)	1.17	No interaction
Rifampicin + PhA2	DNA-dependent RNA polymerase	1.06	No interaction
Moxifloxacin + PhA2	DNA gyrase	1.78	No interaction
Kanamycin + PhA2	30s ribosomal subunit	1.55	No interaction
Bedaquiline + PhA2	ATP synthase	0.50	Synergistic

[a] FICI = $\frac{MIC\ of\ drug\ A\ in\ combination}{MIC\ of\ drug\ A\ alone} + \frac{MIC\ of\ drug\ B\ in\ combination}{MIC\ of\ drug\ B\ alone}$. FICI $\leq$ 0.5 indicates synergy; >4 indicates antagonism; and in-between values are considered to be no interaction between the 2 drugs.

Table S6. Fractional inhibitory concentration indices (FICI) of drug combination with VP as determined in *M. bovis* BCG cultures

Combination (TB drug + VP)	Primary antimycobacterial target of TB drug	FICI[a]	Effect
Isoniazid + VP	NADH-dependent enoyl acyl carrier protein reductase (InhA)	0.64	No interaction
Rifampicin + VP	DNA-dependent RNA polymerase	1.21	No interaction
Moxifloxacin + VP	DNA gyrase	1.22	No interaction
Kanamycin + VP	30s ribosomal subunit	1.30	No interaction
Bedaquiline + VP	ATP synthase	0.30	Synergistic

[a] FICI = $\frac{MIC\ of\ drug\ A\ in\ combination}{MIC\ of\ drug\ A\ alone} + \frac{MIC\ of\ drug\ B\ in\ combination}{MIC\ of\ drug\ B\ alone}$. FICI $\leq$ 0.5 indicates synergy; >4 indicates antagonism; and in-between values are considered to be no interaction between the 2 drugs.

Table S7. Fractional inhibitory concentration indices (FICI) of drug combination with PhA2 as determined in *Mab abscessus* cultures

Combination (*Mab* drug + PhA2)	Primary antimycobacterial target of *Mab* drug	FICI[a]	Effect
Clarithromycin + PhA2	23s RNA	0.95	No interaction
Imipenem + PhA2	L,D-transpeptidase	0.93	No interaction
Amikacin + PhA2	16s RNA	0.69	No interaction
Bedaquiline + PhA2	ATP synthase	0.43	Synergistic

[a] FICI = $\frac{MIC\ of\ drug\ A\ in\ combination}{MIC\ of\ drug\ A\ alone} + \frac{MIC\ of\ drug\ B\ in\ combination}{MIC\ of\ drug\ B\ alone}$. FICI $\leq$ 0.5 indicates synergy; >4 indicates antagonism; and in-between values are considered to be no interaction between the 2 drugs.

Table S8. Fractional inhibitory concentration indices (FICI) of drug combination with VP as determined in *Mab abscessus* cultures

Combination (*Mab* drug + VP)	Primary antimycobacterial target of *Mab* drug	FICI[a]	Effect
Clarithromycin + VP	23s RNA	0.82	No interaction
Imipenem + VP	L,D-transpeptidase	0.86	No interaction
Amikacin + VP	16s RNA	0.71	No interaction
Bedaquiline + VP	ATP synthase	0.50	Synergistic

[a] FICI = $\frac{MIC\ of\ drug\ A\ in\ combination}{MIC\ of\ drug\ A\ alone} + \frac{MIC\ of\ drug\ B\ in\ combination}{MIC\ of\ drug\ B\ alone}$. FICI $\leq$ 0.5 indicates synergy; >4 indicates antagonism; and in-between values are considered to be no interaction between the 2 drugs.

Document S3

Data S1: Characterisation of Final Compounds and Intermediates

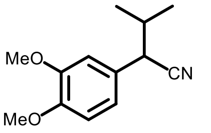

Int 1 for PhA8-PhA10, PhA17-PhA31 and PhA53-PhA64. Yellow oil, 64% yield, ^{1}H NMR (400 MHz, $CDCl_3$) δ 6.80 (d, J = 4 Hz, 2H) 6.75 (s, 1H), 3.83 (s, 3H), 3.82 (s, 3H), 3.56 (d, J = 4 Hz, 1H), 2.08-2.03 (m, 1H), 0.99 (s, 3H), 0.98 (s, 3H). ^{13}C NMR (101 MHz, $CDCl_3$) δ 149.10, 148.69, 127.37, 120.21, 120.05, 111.21, 110.82, 55.92, 55.87, 44.59, 33.73, 20.65, 18.87.

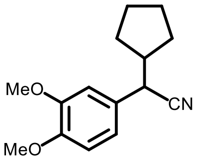

Int 1 for PhA32. White solid, 66% yield. ^{1}H NMR (500 MHz, $CDCl_3$) δ 6.81 – 6.74 (m, 3H), 3.81 (dd, *J* = 11.9, 1.0 Hz, 6H), 3.58 (d, *J* = 7.8 Hz, 1H), 2.23 (h, *J* = 8.1 Hz, 1H), 1.87 – 1.74 (m, 1H), 1.70 – 1.53 (m, 3H), 1.55 – 1.36 (m, 3H), 1.31 – 1.17 (m, 1H). ^{13}C NMR (126 MHz, $CDCl_3$) δ 149.15, 148.65, 128.31, 120.77, 119.90, 111.24, 110.54, 55.92, 55.87, 45.22, 41.98, 30.87, 30.28, 24.87, 24.84.

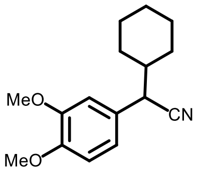

Int 1 for A2. White solid, 73% yield. ^{1}H NMR (500 MHz, $CDCl_3$) δ 6.89 – 6.81 (m, 2H), 6.80 (d, *J* = 1.9 Hz, 1H), 3.91 (d, *J* = 9.7 Hz, 6H), 3.58 (d, *J* = 6.8 Hz, 1H), 1.93 – 1.85 (m, 1H), 1.84 – 1.65 (m, 5H), 1.32 – 1.08 (m, 5H). ^{13}C NMR (126 MHz, $CDCl_3$) δ 149.15, 148.71, 127.09 (2C), 120.37, 111.15, 110.91, 56.02, 55.95, 43.96, 42.83, 31.18, 29.74, 25.94, 25.87, 25.81.

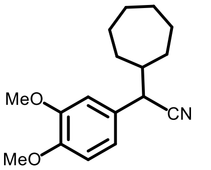

Int 1 for PhA33. White solid, 87% yield. ^{1}H NMR (500 MHz, $CDCl_3$) δ 6.86 (d, *J* = 1.6 Hz, 2H), 6.81 (d, *J* = 1.5 Hz, 1H), 3.91 (d, *J* = 10.1 Hz, 6H), 3.68 (d, *J* = 6.4 Hz, 1H), 1.94 (dq, *J* = 6.7, 3.2 Hz, 1H), 1.89 – 1.79 (m, 1H), 1.72 (tdd, *J* = 9.7, 7.3, 3.0 Hz, 3H), 1.65 – 1.47 (m, 4H), 1.47 – 1.33 (m, 4H). ^{13}C NMR (126 MHz, $CDCl_3$) δ 149.14, 148.67, 127.58, 120.59, 120.36, 111.18, 110.89, 56.02, 55.94, 44.57, 44.37, 33.01, 30.81, 27.84, 27.78, 26.18, 26.05.

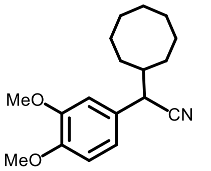

Int 1 for PhA4. White solid. 72% yield. ^{1}H NMR (500 MHz, $CDCl_3$) δ 6.79 – 6.75 (m, 2H), 6.73 (d, J = 1.9 Hz, 1H), 3.80 (s, 3H), 3.78 (s, 3H), 3.54 (d, J = 6.9 Hz, 1H), 1.94 (s, 1H), 1.64 – 1.33 (m, 14H). ^{13}C NMR (126 MHz, $CDCl_3$) δ 148.78, 148.29, 127.31, 120.33, 120.00, 110.85, 110.57, 110.55, 55.61, 55.50, 44.14, 42.15, 30.79, 28.79, 26.36, 26.17, 25.86, 25.03, 24.87.

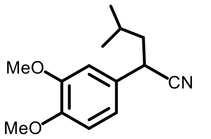

Int 1 for PhA34. White solid, 54% yield. ^{1}H NMR (500 MHz, $CDCl_3$) δ 6.82 – 6.74 (m, 3H), 3.80 (dd, *J* = 15.8, 1.3 Hz, 6H), 3.69 (ddd, *J* = 9.8, 6.6, 1.1 Hz, 1H), 1.88 – 1.68 (m, 2H), 1.54 (dddd, *J* = 13.0, 7.8, 6.4, 1.2 Hz, 1H), 0.90 (ddd, *J* = 6.5, 4.5, 1.2 Hz, 6H). ^{13}C NMR (126 MHz, $CDCl_3$) δ 149.34, 148.71, 128.73, 121.21, 119.49, 111.41, 110.21, 55.91, 55.87, 44.86, 35.00, 26.00, 22.49, 21.59.

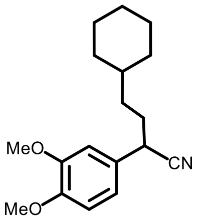

Int 1 for PhA1 and PhA68. White solid, 82% yield. ^{1}H NMR (400 MHz, $CDCl_3$) δ 6.78 (d, J = 1.6 Hz, 2H), 6.74 (d, J = 1.2 Hz, 1H), 3.83 (s, 3H), 3.80 (s, 3H), 3.60 (dd, J = 8.5, 6.4 Hz, 1H), 1.92 – 1.71 (m, 2H), 1.66 – 1.18 (m, 13H). ^{13}C NMR (101 MHz, $CDCl_3$) δ 149.35, 148.75, 128.53, 121.18, 119.56, 111.41, 110.25, 56.01, 55.96, 37.26, 37.22, 34.64, 33.47, 33.23, 33.03, 29.69, 26.50, 26.22.

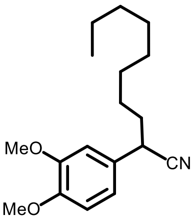

Int 1 for PhA2 and PhA67. Colourless oil, 63% yield. ^{1}H NMR (400 MHz, $CDCl_3$) δ 6.76 – 6.73 (m, 2H), 6.72 (d, J = 1.5 Hz, 1H), 3.77 (s, 3H), 3.75 (s, 3H), 3.61 (dd, J = 8.5, 6.3 Hz, 1H), 1.86 – 1.66 (m, 2H), 1.15 (d, J = 3.1 Hz, 12H), 0.80 – 0.74 (m, 3H). ^{13}C NMR (101 MHz, $CDCl_3$) δ 149.29, 148.70, 128.50, 121.10, 119.48, 111.38, 110.26, 55.88, 55.84, 36.86, 35.86, 31.73, 29.23, 29.10, 28.91, 26.99, 22.56, 14.02

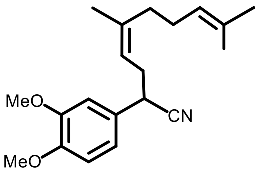

Int 1 for PhA3 and PhA16. Colourless oil, 72% yield. ^{1}H NMR (400 MHz, $CDCl_3$) δ 6.78 – 6.69 (m, 3H), 5.12 – 5.04 (m, 1H), 5.00 – 4.93 (m, 1H), 3.77 (s, 3H), 3.75 (s, 3H), 3.63 (dd, J = 7.8, 6.5 Hz, 1H), 2.58 – 2.38 (m, 2H), 2.01 – 1.84 (m, 4H), 1.57 (d, J = 1.4 Hz, 3H), 1.48 (d, J = 1.4 Hz, 3H), 1.45 (d, J = 1.4 Hz, 3H). ^{13}C NMR (101 MHz, $CDCl_3$) □ 149.20, 148.73, 139.95, 131.50, 128.11, 123.87, 120.94, 119.69, 118.50, 111.32, 110.43, 55.91, 55.87, 39.64, 37.26, 34.32, 26.47, 25.63, 17.64, 16.21.

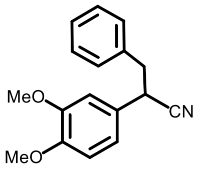

Int 1 for PhA35. White solid, 66% yield. ^{1}H NMR (500 MHz, $CDCl_3$) δ 7.33 – 7.26 (m, 3H), 7.16 – 7.10 (m, 2H), 6.82 (s, 2H), 6.66 (d, *J* = 1.5 Hz, 1H), 3.97 (dd, *J* = 7.4, 6.0 Hz, 1H), 3.87 (s, 3H), 3.80 (s, 3H), 3.18 (dd, *J* = 13.5, 8.0 Hz, 1H), 3.11 (dd, *J* = 13.5, 6.6 Hz, 1H). ^{13}C NMR (126 MHz, $CDCl_3$) δ 149.14, 148.87, 136.36, 130.45, 129.35, 128.59, 128.10, 127.52, 127.33, 120.67, 119.80, 111.35, 110.67, 55.94, 55.91, 42.17, 39.20.

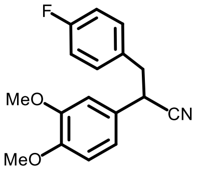

Int 1 for PhA36. White solid, 65%. ^{1}H NMR (500 MHz, $CDCl_3$) δ 7.10 – 7.03 (m, 1H), 7.02 – 6.92 (m, 2H), 6.92 – 6.74 (m, 3H), 6.68 (dd, *J* = 18.1, 2.2 Hz, 1H), 3.94 (dd, *J* = 7.6, 6.5 Hz, 1H), 3.86 (s, 3H), 3.76 (s, 3H), 3.30 – 3.20 (m, 1H), 3.18 – 3.06 (m, 1H). ^{13}C NMR (126 MHz, $CDCl_3$) δ 148.84, 148.67, 131.92, 130.97, 128.97, 127.18, 121.19, 119.85, 119.12, 115.08, 114.91, 111.00, 110.34, 55.91, 55.84, 45.62, 41.26.

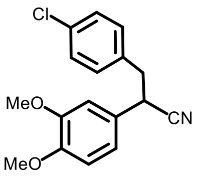

Int 1 for PhA37. White solid, 65% yield. ^{1}H NMR (500 MHz, $CDCl_3$) δ 7.31 – 7.22 (m, 1H), 7.21 – 7.13 (m, 1H), 7.06 – 7.01 (m, 1H), 7.01 – 6.92 (m, 1H), 6.87 – 6.74 (m, 2H), 6.67 (dd, *J* = 15.2, 2.2 Hz, 1H), 3.94 (dd, *J* = 7.8, 6.7 Hz, 1H), 3.87 (s, 3H), 3.77 (s, 3H), 3.29 – 3.20 (m, 1H), 3.18 – 3.05 (m, 1H). ^{13}C NMR (126 MHz, $CDCl_3$) δ 148.88, 148.74, 134.72, 133.41, 131.66, 130.73, 128.70, 128.31, 121.02, 119.86, 119.11, 111.02, 110.26, 55.93, 55.86, 50.62, 45.80.

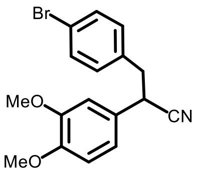

Int 1 for PhA5. White solid, 51% yield. ^{1}H NMR (500 MHz, $CDCl_3$) δ 7.01 – 6.95 (m, 1H), 6.95 – 6.72 (m, 5H), 6.65 (dd, *J* = 9.7, 2.1 Hz, 1H), 3.99 – 3.90 (m, 1H), 3.88 (s, 3H), 3.77 (s, 3H), 3.29 – 3.04 (m, 2H). ^{13}C NMR (126 MHz, $CDCl_3$) δ 148.88, 148.76, 133.83, 131.99, 131.71, 131.31, 131.07, 121.61, 119.87, 119.05, 111.04, 110.51, 110.31, 55.97, 45.93, 41.53, 38.99.

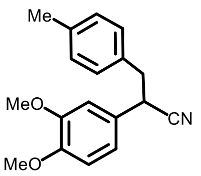

Int 1 for PhA38. White solid, 63% yield. ^{1}H NMR (500 MHz, $CDCl_3$) δ 7.12 (d, *J* = 7.8 Hz, 2H), 7.04 (dd, *J* = 8.2, 6.7 Hz, 2H), 6.90 – 6.79 (m, 2H), 6.68 (d, *J* = 1.7 Hz, 1H), 4.01 – 3.90 (m, 1H), 3.90 (s, 3H), 3.84 (s, 3H), 3.32 – 3.06 (m, 2H), 2.35 (s, 3H). ^{13}C NMR (126 MHz, $CDCl_3$) δ 149.15, 148.86, 136.96, 133.30, 130.30, 129.28, 129.18, 128.80, 127.65, 120.70, 119.80, 111.33, 110.65, 55.95, 55.91, 41.86, 39.41, 21.09.

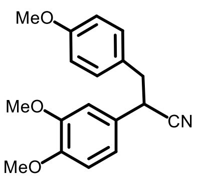

Int 1 for PhA39. White solid, 65% yield. ^{1}H NMR (500 MHz, $CDCl_3$) δ 7.07 – 7.01 (m, 1H), 6.98 – 6.93 (m, 1H), 6.86 – 6.78 (m, 3H), 6.75 – 6.72 (m, 1H), 6.66 (d, *J* = 2.0 Hz, 1H), 3.95 – 3.86 (m, 1H), 3.86 (s, 3H), 3.77 (s, 3H), 3.73 (s, 3H), 3.27 – 3.03 (m, 2H). ^{13}C NMR (126 MHz, $CDCl_3$) δ 149.40, 149.12, 148.82, 148.42, 131.43, 130.39, 120.25, 113.94, 113.48, 111.55, 111.34, 111.03, 110.67, 55.96, 45.61, 41.35, 39.45, 23.11.

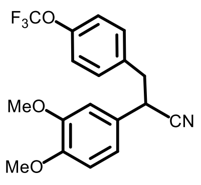

Int 1 for PhA40. White solid, 70% yield. ^{1}H NMR (500 MHz, $CDCl_3$) δ 7.21 – 7.15 (m, 2H), 7.14 (s, 2H), 6.94 – 6.77 (m, 2H), 6.64 (dd, *J* = 15.2, 2.2 Hz, 1H), 4.00 – 3.94 (m, 1H), 3.89 (s, 3H), 3.80 (s, 3H), 3.35 – 3.01 (m, 2H). ^{13}C NMR (126 MHz, $CDCl_3$) δ 149.21, 149.02, 148.80, 148.56, 133.56, 131.72, 130.81, 126.95, 121.04, 120.53, 119.80, 111.37, 110.55, 55.92, 45.71, 41.37, 39.03, 29.69.

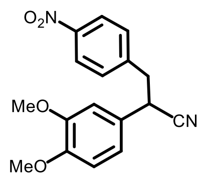

Int 1 for PhA41. White solid, 10% yield. ^{1}H NMR (500 MHz, $CDCl_3$) δ 8.25 – 8.12 (m, 2H), 7.30 (dd, *J* = 9.0, 2.2 Hz, 2H), 6.91 – 6.69 (m, 3H), 4.05 (t, *J* = 7.1 Hz, 1H), 3.89 (s, 3H), 3.85 (s, 3H), 3.34 – 3.23 (m, 2H). ^{13}C NMR (126 MHz, $CDCl_3$) δ 149.46, 149.25, 147.37, 143.54, 130.34, 126.39, 123.76, 120.26, 119.91, 119.83, 111.48, 111.03, 110.36, 55.98, 41.65, 38.58, 23.20.

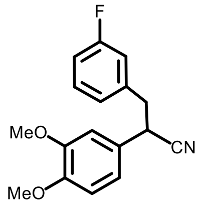

Int 1 for PhA42. White solid, 49% yield. ^{1}H NMR (500 MHz, $CDCl_3$) δ 7.16 (td, *J* = 8.0, 6.0 Hz, 1H), 6.99 – 6.67 (m, 6H), 3.98 – 3.95 (m, 1H), 3.86 (s, 3H), 3.77 (s, 3H), 3.28 (d, *J* = 3.1 Hz, 1H), 3.14 (qd, *J* = 13.6, 7.3 Hz, 1H). ^{13}C NMR (126 MHz, $CDCl_3$) δ 149.27, 149.03, 148.95, 148.83, 137.38, 129.62, 126.13, 119.01, 117.15, 114.40, 114.23, 111.12, 110.37, 55.93, 50.25, 46.11, 38.91.

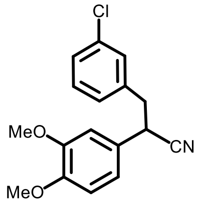

Int 1 for PhA6. White solid, 40% yield. ^{1}H NMR (500 MHz, $CDCl_3$) δ 7.24 – 7.17 (m, 1H), 7.12 (t, *J* = 7.8 Hz, 1H), 7.04 – 6.98 (m, 1H), 6.93 (dt, *J* = 7.6, 1.5 Hz, 1H), 6.88 – 6.75 (m, 2H), 6.70 (dd, *J* = 17.3, 2.1 Hz, 1H), 3.98 – 3.89 (m, 1H), 3.86 (s, 3H), 3.77 (s, 3H), 3.28 – 3.19 (m, 1H), 3.11 (qd, *J* = 13.6, 7.3 Hz, 1H). ^{13}C NMR (126 MHz, $CDCl_3$) δ 149.00, 148.92, 136.91, 133.83, 130.46, 129.43, 128.56, 127.59, 120.93, 119.84, 119.12, 111.24, 110.47, 55.97, 50.18, 45.93, 38.91.

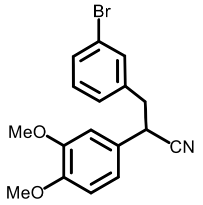

Int 1 for PhA43. White solid, 20% yield. ^{1}H NMR (500 MHz, $CDCl_3$) δ 7.20 (dt, *J* = 8.0, 1.5 Hz, 1H), 7.14 (t, *J* = 7.8 Hz, 1H), 7.03 (t, *J* = 1.9 Hz, 1H), 6.95 (dt, *J* = 7.6, 1.4 Hz, 1H), 6.91 – 6.81 (m, 2H), 6.74 (dd, *J* = 18.1, 2.1 Hz, 1H), 3.94 – 3.90 (m, 1H), 3.89 (s, 3H), 3.79 (s, 3H), 3.30 – 3.06 (m, 2H). ^{13}C NMR (126 MHz, $CDCl_3$) δ 149.01, 148.94, 136.86, 133.89, 130.47, 129.43, 128.74, 128.54, 127.62, 120.91, 119.07, 111.24, 110.49, 56.12, 55.99, 50.16, 45.99.

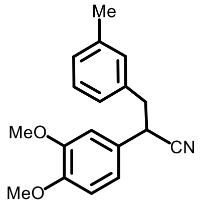

Int 1 for PhA44. White solid, 57% yield. ^{1}H NMR (500 MHz, $CDCl_3$) δ 7.21 (t, *J* = 7.5 Hz, 1H), 7.10 (d, *J* = 7.6 Hz, 1H), 6.98 – 6.91 (m, 1H), 6.92 – 6.80 (m, 3H), 6.76 – 6.68 (m, 1H), 4.01 – 3.90 (m, 1H), 3.90 (s, 3H), 3.84 (s, 3H), 3.31 – 3.07 (m, 2H), 2.34 (s, 3H). ^{13}C NMR (126 MHz, $CDCl_3$) δ 149.16, 148.87, 138.24, 136.33, 130.04, 128.51, 128.08, 127.70, 126.31, 120.68, 119.79, 111.32, 110.61, 55.97, 42.24, 39.34, 29.72, 21.37.\

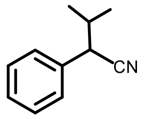

Int 1 for PhA45. Colourless liquid, 87% yield. ^{1}H NMR (500 MHz, $CDCl_3$) δ 7.48 – 7.19 (m, 5H), 3.68 (d, *J* = 6.2 Hz, 1H), 2.11 (dq, *J* = 13.3, 6.6 Hz, 1H), 1.04 (dd, *J* = 11.8, 6.8 Hz, 6H). ^{13}C NMR (126 MHz, $CDCl_3$) δ 133.57, 133.41, 129.30, 129.02, 119.53, 77.81, 76.13, 76.21, 44.54, 33.27, 28.76, 21.79, 17.90.

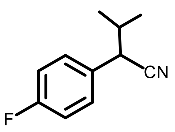

Int 1 for PhA46. White solid, 68% yield. ^{1}H NMR (400 MHz, $CDCl_3$) δ 7.32 – 7.24 (m, 2H), 7.06 (t, J = 8.6 Hz, 2H), 3.66 (d, J = 6.3 Hz, 1H), 2.09 (dt, J = 13.3, 6.7 Hz, 1H), 1.03 (t, J = 6.8 Hz, 6H). ^{13}C NMR (101 MHz, $CDCl_3$) δ 163.53, 130.81, 130.77, 129.57, 129.49, 119.71, 115.85, 115.63, 44.28, 33.79, 20.58, 18.71.

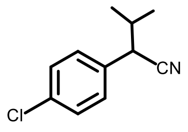

Int 1 for PhA47. Yellow oil, 64% yield. ^{1}H NMR (500 MHz, $CDCl_3$) δ 7.43 – 7.22 (m, 4H), 3.67 (d, *J* = 6.2 Hz, 1H), 2.21 – 2.00 (m, 1H), 1.05 (dd, *J* = 16.5, 6.5 Hz, 6H).^{13}C NMR (126 MHz, $CDCl_3$) δ 133.97, 133.46, 129.23, 129.03, 119.45, 77.38, 77.13, 76.87, 44.50, 33.77, 29.72, 20.70, 18.72.

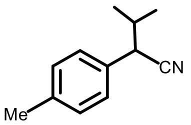

Int 1 for PhA48. White solid, 66% yield. ^{1}H NMR (400 MHz, $CDCl_3$) δ 7.22 (d, J = 8.2 Hz, 2H), 7.18 (d, J = 8.4 Hz, 2H), 3.65 (d, J = 6.3 Hz, 1H), 2.36 (s, 3H), 2.18 – 2.04 (m, 1H), 1.05 (t, J = 7.0 Hz, 6H). ^{13}C NMR (101 MHz, $CDCl^3$) δ 137.64, 132.00, 129.48, 127.76, 120.04, 44.65, 33.75, 21.03, 20.72, 18.80.

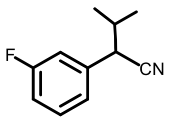

Int 1 for PhA49. 63% yield. ^{1}H NMR (400 MHz, $CDCl_3$) δ 7.41 – 7.33 (m, 1H), 7.12 (d, J = 7.8 Hz, 1H), 7.06 (d, J = 9.5 Hz, 2H), 3.69 (d, J = 6.2 Hz, 1H), 2.21 – 2.08 (m, 1H), 1.09 (d, J = 6.7 Hz, 3H), 1.06 (d, J = 6.7 Hz, 3H). ^{13}C NMR (101 MHz, $CDCl_3$) δ 164.08, 161.62, 137.34, 137.27, 130.47, 130.38, 123.58, 123.55, 119.27, 115.16, 115.12, 114.95, 114.89, 44.83, 44.81, 33.76, 29.71, 20.78, 18.73.

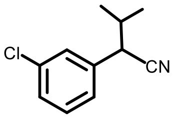

Int 1 for PhA50. Yellow oil, 61% yield. ^{1}H NMR (400 MHz, $CDCl_3$) δ 7.31 (s, 1H), 7.31 (s, 1H), 7.30 (s, 1H), 7.23 – 7.18 (m, 1H), 3.66 (d, J = 6.2 Hz, 1H), 2.19 – 2.05 (m, 1H), 1.06 (d, J = 6.8 Hz, 3H), 1.02 (d, J = 6.8 Hz, 3H). ^{13}C NMR (101 MHz, $CDCl_3$) δ 136.95, 134.71, 130.13, 128.24, 127.96, 126.09, 119.21, 44.66, 33.73, 20.75, 18.68.

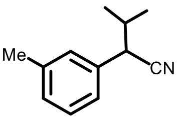

Int 1 for PhA51. White solid, 60% yield. ^{1}H NMR (400 MHz, $CDCl_3$) δ 7.27 (t, J = 7.9 Hz, 1H), 7.19 – 7.09 (m, 3H), 3.65 (d, J = 6.3 Hz, 1H), 2.38 (s, 3H), 2.19 – 2.08 (m, 1H), 1.07 (dd, J = 8.3, 6.7 Hz, 6H). ^{13}C NMR (101 MHz, $CDCl_3$) δ 138.56, 134.93, 128.69, 128.50, 124.97, 119.98, 44.99, 33.74, 21.39, 20.83, 18.82.

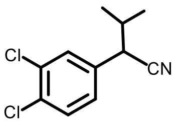

Int 1 for PhA7 and PhA66. Yellow oil, 70% yield. ^{1}H NMR (400 MHz, $CDCl_3$) δ 7.33 (d, J = 8.3 Hz, 1H), 7.30 (d, J = 2.2 Hz, 1H), 7.06 (dd, J = 8.3, 2.2 Hz, 1H), 3.57 (d, J = 6.1 Hz, 1H), 1.99 (dq, J = 13.3, 6.7 Hz, 1H), 0.94 (d, J = 6.7 Hz, 3H), 0.89 (d, J = 6.7 Hz, 3H). ^{13}C NMR (101 MHz, $CDCl_3$) δ 135.24, 132.91, 132.19, 130.76, 129.75, 127.27, 118.88, 44.07, 33.66, 20.65, 18.56.

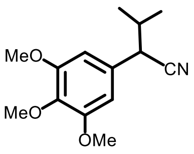

Int 1 for PhA52. White solid, ^{1}H NMR (500 MHz, $CDCl_3$) δ 6.39 (s, 2H), 3.69 (d, *J* = 11.3 Hz, 9H), 3.49 (d, *J* = 6.5 Hz, 1H), 1.97 (h, *J* = 6.7 Hz, 1H), 1.17 – 0.84 (m, 7H). ^{13}C NMR (126 MHz, $CDCl_3$) δ 170.75, 153.25, 137.41, 130.64, 119.74, 106.32, 104.85, 104.84, 77.72, 77.46, 77.21, 60.52, 60.10, 55.96, 55.84, 44.93, 36.47, 33.56, 31.74, 29.51, 29.47, 29.17, 25.64, 24.53, 23.30, 22.51, 22.04, 20.73, 20.59, 18.71, 14.00, 13.94, 13.66.

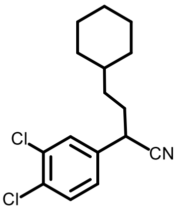

Int 1 for PhA11. Yellow oil, 63% yield. ^{1}H NMR (400 MHz, $CDCl_3$) δ 7.49 – 7.32 (m, 2H), 7.14 (dd, *J* = 8.4, 2.2 Hz, 1H), 3.70 (dd, *J* = 8.3, 6.4 Hz, 1H), 2.02 – 1.72 (m, 2H), 1.72 – 1.47 (m, 5H), 1.43 – 0.99 (m, 7H), 0.99 – 0.62 (m, 3H). ^{13}C NMR (101 MHz, $CDCl_3$) δ 136.57, 133.28, 132.40, 131.14, 129.44, 126.87, 120.14, 37.30, 36.94, 34.66, 33.34, 33.11, 26.65, 26.38, 26.36.

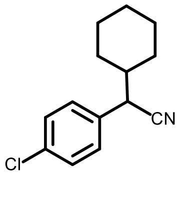

Int 1 for PhA14. Yellow oil, 88% yield. ^{1}H NMR (400 MHz, $CDCl_3$) δ 7.28 – 7.00 (m, 4H), 3.51 (d, *J* = 6.6 Hz, 1H), 1.87 – 1.41 (m, 7H), 1.41 – 0.67 (m, 10H). ^{13}C NMR (101 MHz, $CDCl_3$) δ 133.85, 133.31, 129.34, 128.94, 119.66, 43.62, 42.69, 31.94, 31.09, 29.72, 29.68, 29.46, 29.38, 26.92, 25.88, 25.79, 25.75, 22.71, 14.14.

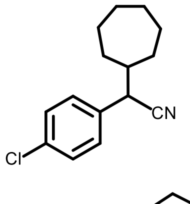

Int 1 for PhA65. Yellow oil, 65% yield. ^{1}H NMR (400 MHz, $CDCl_3$) δ 7.40 – 7.12 (m, 4H), 3.73 (d, *J* = 6.2 Hz, 1H), 2.09 – 0.79 (m, 16H). ^{13}C NMR (101 MHz, $CDCl_3$) δ 133.81, 133.78, 129.34, 128.98, 119.91, 44.45, 44.10, 33.05, 30.58, 29.73, 27.77, 27.71, 26.14, 26.00.

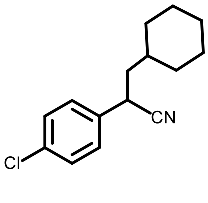

Int 1 for PhA15. Yellow oil, 78% yield. ^{1}H NMR (400 MHz, $CDCl_3$) δ 7.43 – 6.93 (m, 4H), 3.71 (dd, *J* = 9.9, 6.2 Hz, 1H), 1.94 – 0.54 (m, 17H). ^{13}C NMR (101 MHz, $CDCl_3$) δ 135.14, 133.83, 129.57, 129.22, 128.79, 128.62, 120.62, 43.49, 35.28, 34.18, 33.39, 33.20, 32.29, 29.74, 26.30, 26.16, 25.94, 25.86.

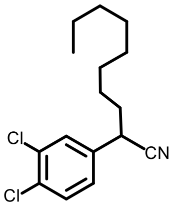

Int 1 for PhA12. Yellow oil, 59% yield. ^{1}H NMR (400 MHz, $CDCl_3$) δ 7.42 – 7.32 (m, 2H), 7.14 (dd, *J* = 8.3, 2.2 Hz, 1H), 3.84 – 3.69 (m, 2H), 3.60 – 3.32 (m, 1H), 1.90 – 1.71 (m, 3H), 1.53 – 1.07 (m, 19H), 1.01 – 0.69 (m, 4H). ^{13}C NMR (101 MHz, $CDCl_3$) δ 136.35, 133.03, 132.15, 130.91, 129.22, 126.65, 119.88, 62.59, 62.57, 62.55, 55.85, 55.80, 47.83, 44.56, 40.78, 36.43, 35.55, 33.88, 32.82, 32.75, 31.74, 29.49, 29.40, 29.36, 29.32, 29.19, 29.15, 29.10, 28.85, 28.75, 28.73, 28.14, 26.89, 25.78, 22.59, 14.05.

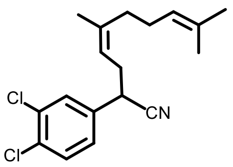

Int 1 for PhA13. Yellow oil, 73% yield. ^{1}H NMR (400 MHz, $CDCl_3$) δ 7.53 – 7.00 (m, 3H), 5.14 – 4.79 (m, 2H), 3.69 (t, *J* = 7.0 Hz, 1H), 2.50 (hept, *J* = 7.4 Hz, 2H), 2.08 – 1.65 (m, 5H), 1.73 – 0.67 (m, 13H). ^{13}C NMR (101 MHz, $CDCl_3$) δ 140.97, 135.83, 135.81, 132.99, 132.27, 131.65, 130.89, 130.85, 130.83, 129.46, 126.82, 123.81, 119.78, 117.74, 117.62, 110.07, 39.64, 36.83, 33.95, 29.73, 26.45, 25.69, 25.66, 17.68, 17.65, 16.23

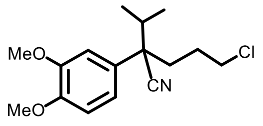

Int 2 for PhA8, PhA17-PhA31, PhA53-PhA57. Yellow oil, 44% yield.^{1}H NMR (400 MHz, $CDCl_3$) δ 6.80 (dd, J = 8 Hz, 4Hz, 2H), 6.71 (d, J = 8 Hz, 1H) 3.72 (s, 3H), 3.69 (s, 3H), 3.33-3.29 (m, 2H), 2.08-1.86 (m, 3H), 1.73-1.63 (m, 1H), 1.32-1.29 (m. 1H), 1.03 (d, J = 8Hz, 3H) 0.65 (d, J = 8Hz 3H). ^{13}C NMR (101 MHz, $CDCl_3$) δ 149.10, 148.40, 129.92, 120.91, 118.82, 111.10, 109.28, 55.83, 55.69, 52.76, 44.49, 37.74, 34.87, 28.58, 18.76, 18.38.

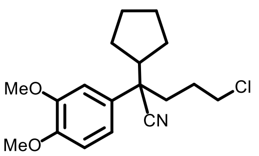

Int 2 for PhA32. Yellow oil, 33% yield. ^{1}H NMR (500 MHz, $CDCl_3$) δ 6.94 (dd, *J* = 8.4, 2.3 Hz, 1H), 6.88 – 6.79 (m, 2H), 3.86 (d, *J* = 8.7 Hz, 6H), 3.51 – 3.38 (m, 2H), 2.34 – 2.22 (m, 1H), 2.15 (ddd, *J* = 13.7, 11.7, 4.8 Hz, 1H), 2.06 (ddd, *J* = 13.6, 11.6, 4.5 Hz, 1H), 2.01 – 1.93 (m, 1H), 1.86 (ddt, *J* = 13.8, 11.3, 5.5 Hz, 1H), 1.74 (ddd, *J* = 14.9, 8.9, 5.5 Hz, 1H), 1.62 – 1.54 (m, 3H), 1.52 – 1.38 (m, 2H), 1.37 – 1.20 (m, 2H). ^{13}C NMR (126 MHz, $CDCl_3$) δ 149.12, 148.40, 130.72, 121.50, 118.51, 111.13, 109.12, 56.01, 55.89, 52.14, 50.20, 44.66, 36.73, 29.52, 29.19, 28.35, 25.46, 24.81.

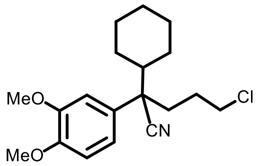

Int 2 for A2. Yellow oil, 75% yield. ^{1}H NMR (500 MHz, $CDCl_3$) δ 6.91 (dd, *J* = 8.4, 2.2 Hz, 1H), 6.87 – 6.81 (m, 2H), 3.89 (d, *J* = 6.8 Hz, 6H), 3.56 – 3.41 (m, 2H), 2.24 (ddd, *J* = 13.6, 11.7, 4.5 Hz, 1H), 2.10 – 1.99 (m, 2H), 1.91 – 1.79 (m, 2H), 1.72 – 1.57 (m, 3H), 1.51 – 1.36 (m, 1H), 1.36 – 1.19 (m, 3H), 1.19 – 0.99 (m, 3H). ^{13}C NMR (126 MHz, $CDCl_3$) δ 149.12, 148.41, 129.86, 121.65, 118.89, 111.11, 109.53, 56.05, 55.90, 52.30, 47.46, 44.82, 34.59, 28.72, 28.67, 28.45, 26.30, 26.26, 25.91.

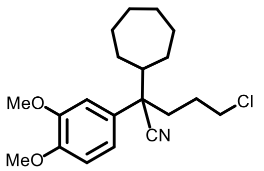

Int 2 for PhA33. Yellow oil, 71% yield. ^{1}H NMR (500 MHz, $CDCl_3$) δ 6.92 (dd, *J* = 8.4, 2.2 Hz, 1H), 6.83 (dd, *J* = 5.4, 3.1 Hz, 2H), 3.88 (d, *J* = 6.8 Hz, 6H), 3.54 – 3.39 (m, 2H), 2.22 (ddd, *J* = 13.4, 11.8, 4.4 Hz, 1H), 2.08 – 1.96 (m, 2H), 1.92 – 1.70 (m, 3H), 1.65 – 1.57 (m, 1H), 1.56 – 1.46 (m, 4H), 1.47 – 1.34 (m, 3H), 1.33 – 1.14 (m, 3H). ^{13}C NMR (126 MHz, $CDCl_3$) δ 149.12, 148.37, 130.44, 121.91, 118.98, 111.11, 109.57, 56.06, 55.88, 52.82, 48.79, 44.77, 35.15, 30.20, 29.95, 28.55, 28.08, 27.44, 27.14, 26.76.

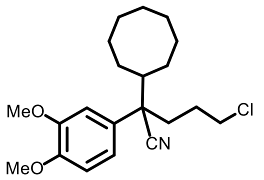

Int 2 for PhA4. Yellow oil, 52% yield. ^{1}H NMR (400 MHz, $CDCl_3$) δ 6.94 (dd, J = 8.4, 2.2 Hz, 1H), 6.89 – 6.81 (m, 2H), 3.89 (s, 3H), 3.88 (s, 3H), 3.52 – 3.40 (m, 2H), 2.10 – 1.96 (m, 2H), 1.90 (dd, J = 8.5, 2.8 Hz, 1H), 1.85 – 1.76 (m, 2H), 1.72 – 1.29 (m, 14H). ^{13}C NMR (101 MHz, $CDCl_3$) δ 149.06, 148.34, 130.45, 121.78, 118.99, 111.10, 109.59, 56.03, 55.85, 53.74, 46.07, 44.71, 35.24, 29.76, 29.67, 29.55, 28.53, 26.95, 26.61, 26.60, 26.06, 25.77.

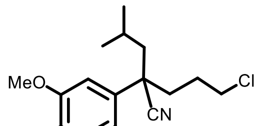

Int 2 for PhA34. Yellow oil, 36% yield. ^{1}H NMR (500 MHz, $CDCl_3$) δ 7.01 – 6.94 (m, 1H), 6.92 – 6.83 (m, 2H), 3.90 (d, *J* = 8.3 Hz, 6H), 3.48 (t, *J* = 6.1 Hz, 2H), 2.17 – 1.81 (m, 4H), 1.72 – 1.49 (m, 2H), 1.31 – 1.22 (m, 1H), 1.01 (d, *J* = 6.6 Hz, 3H), 0.71 (d, *J* = 6.6 Hz, 3H). ^{13}C NMR (126 MHz, $CDCl_3$) δ 149.20, 148.51, 130.26, 122.76, 118.51, 111.20, 109.07, 56.05, 55.89, 49.75, 46.31, 44.53, 39.69, 28.08, 25.65, 23.90, 23.35.

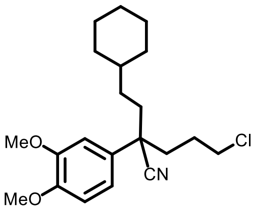

Int 2 for PhA1 and PhA68. Yellow oil, 60% yield. ^{1}H NMR (400 MHz, $CDCl_3$) δ 6.93 (dd, J = 8.4, 2.2 Hz, 1H), 6.87 – 6.83 (m, 2H), 3.89 (s, 3H), 3.87 (s, 3H), 3.47 (dd, J = 6.6, 5.8 Hz, 2H), 2.07 (ddd, J = 10.3, 7.4, 5.1 Hz, 2H), 2.03 – 1.92 (m, 2H), 1.92 – 1.70 (m, 2H), 1.70 – 1.10 (m, 12H). ^{13}C NMR (101 MHz, $CDCl_3$) □ 149.21, 148.48, 130.36, 122.39, 118.33, 111.25, 108.93, 56.01, 55.88, 47.41, 44.52, 38.85, 38.30, 37.52, 33.16, 32.98, 32.56, 28.32, 26.48, 26.20, 26.17.

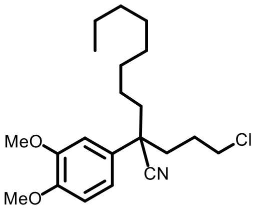

Int 2 for PhA2 and PhA67. Yellow oil, 58% yield. ^{1}H NMR (400 MHz, $CDCl_3$) δ 6.87 (d, J = 2.3 Hz, 1H), 6.78 (d, J = 8.5 Hz, 2H), 3.81 (s, 3H), 3.77 (s, 3H), 3.38 (t, J = 6.2 Hz, 2H), 2.01 (ddd, J = 9.6, 5.4, 3.1 Hz, 2H), 1.90 – 1.78 (m, 2H), 1.72 – 1.06 (m, 14H), 0.76 (t, J = 6.9 Hz, 3H). ^{13}C NMR (101 MHz, $CDCl_3$) □ 149.20, 148.47, 130.32, 122.27, 118.29, 111.22, 108.90, 55.88, 55.75, 47.31, 44.38, 41.31, 38.08, 31.68, 29.28, 29.13, 29.03, 28.30, 25.11, 22.50, 13.99.

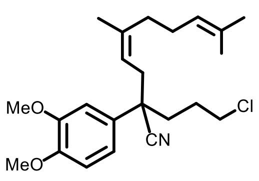

Int 2 for PhA3 and PhA16. Yellow oil, 53% yield. ^{1}H NMR (400 MHz, $CDCl_3$) δ 6.87 (dd, J = 8.4, 2.3 Hz, 1H), 6.83 – 6.73 (m, 2H), 5.06 – 4.96 (m, 1H), 4.96 – 4.86 (m, 1H), 3.80 (s, 3H), 3.77 (s, 3H), 3.45 – 3.35 (m, 2H), 2.66 – 2.43 (m, 2H), 2.09 – 1.98 (m, 2H), 1.89 (p, J = 7.7, 7.2 Hz, 5H), 1.56 (d, J = 1.5 Hz, 3H), 1.47 (d, J = 1.3 Hz, 2H), 1.46 (d, J = 1.4 Hz, 3H). ^{13}C NMR (101 MHz, $CDCl_3$) δ 149.07, 148.50, 140.21, 131.34, 130.25, 123.87, 122.27, 118.52, 117.55, 111.16, 109.29, 55.92, 55.81, 47.40, 44.50, 39.97, 39.72, 36.69, 28.32, 26.40, 25.61, 17.61, 16.38.

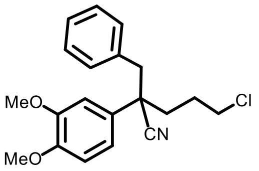

Int 2 for PhA35. Yellow oil, 29% yield. ^{1}H NMR (500 MHz, $CDCl_3$) δ 7.24 – 7.16 (m, 3H), 7.08 – 7.01 (m, 1H), 7.01 – 6.92 (m, 1H), 6.92 – 6.77 (m, 2H), 6.70 (dd, *J* = 20.1, 2.2 Hz, 1H), 3.88 (s, 3H), 3.80 (s, 3H), 3.58 – 3.46 (m, 2H), 3.29 (d, *J* = 13.6 Hz, 1H), 3.15 (d, *J* = 13.4 Hz, 1H), 2.30 (ddd, *J* = 13.6, 11.9, 4.3 Hz, 1H), 2.14 (ddd, *J* = 13.7, 11.7, 4.5 Hz, 1H), 2.06 – 1.92 (m, 1H), 1.75 – 1.63 (m, 1H). ^{13}C NMR (126 MHz, $CDCl_3$) δ 149.02, 148.65, 135.09, 134.78, 130.44, 130.39, 128.12, 127.36, 127.27, 119.01, 118.69, 111.15, 109.76, 55.98, 55.92, 48.32, 46.54, 44.59, 36.47, 28.32.

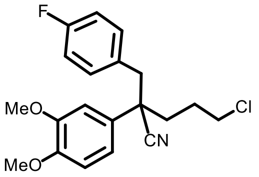

Int 2 for PhA36. Yellow oil, 30% yield. ^{1}H NMR (500 MHz, $CDCl_3$) δ 6.97 – 6.82 (m, 6H), 6.74 (d, *J* = 1.8 Hz, 1H), 3.91 (s, 3H), 3.84 (s, 3H), 3.55 (m, 2H), 3.21 – 3.10 (m, 2H), 2.32 (ddd, *J* = 13.6, 11.9, 4.3 Hz, 1H), 2.17 (ddd, *J* = 13.7, 11.7, 4.6 Hz, 1H), 2.01 (ddt, *J* = 13.4, 11.3, 5.4 Hz, 1H), 1.75 – 1.63 (m, 1H). ^{13}C NMR (126 MHz, $CDCl_3$) δ 149.11, 148.73, 131.88 (2C), 131.82, 130.55, 129.03, 121.76, 118.75, 115.07, 114.90, 111.18, 109.60, 56.01, 55.92, 48.83, 47.45, 44.54, 36.58, 28.28.

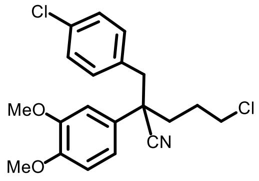

Int 2 for PhA37. Yellow oil, 20% yield. ^{1}H NMR (500 MHz, $CDCl_3$) δ 7.23 – 7.13 (m, 2H), 7.00 – 6.93 (m, 2H), 6.93 – 6.78 (m, 2H), 6.75 – 6.66 (m, 1H), 3.89 (s, 3H), 3.83 (s, 3H), 3.57 – 3.47 (m, 2H), 3.31 – 3.04 (m, 2H), 2.31 (ddd, *J* = 13.8, 11.8, 4.4 Hz, 1H), 2.16 (ddd, *J* = 13.8, 11.6, 4.7 Hz, 1H), 1.99 (dddd, *J* = 16.6, 14.5, 7.9, 3.2 Hz, 1H), 1.74 – 1.63 (m, 1H). ^{13}C NMR (126 MHz, $CDCl_3$) δ 149.14, 148.75, 133.36, 131.64, 128.34, 121.67, 121.01, 119.08, 118.78, 111.21, 111.04, 110.31, 109.56, 56.02, 55.89, 48.70, 45.84, 36.64, 29.70, 28.28.

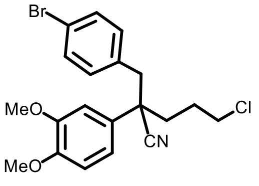

Int 2 for PhA5. Yellow oil, 41% yield. ^{1}H NMR (500 MHz, $CDCl_3$) δ 7.00 – 6.82 (m, 6H), 6.71 (d, *J* = 1.8 Hz, 1H), 3.87 (s, 3H), 3.81 (s, 3H), 3.53 – 3.46 (m, 2H), 3.17 – 3.07 (m, 2H), 2.29 (ddd, *J* = 13.7, 11.9, 4.3 Hz, 1H), 2.13 (ddd, *J* = 13.7, 11.7, 4.7 Hz, 1H), 2.03 – 1.91 (m, 1H), 1.66 (dddd, *J* = 19.8, 12.1, 8.7, 4.8 Hz, 1H). ^{13}C NMR (126 MHz, $CDCl_3$) δ 149.11, 148.73, 131.88, 131.82, 130.57, 130.54, 129.03, 121.76, 118.76, 115.06, 114.89, 111.19, 109.61, 56.01, 48.82, 47.43, 44.54, 36.58, 29.70, 28.29.

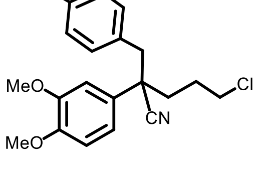

Int 2 for PhA38. Yellow oil, 38% yield. ^{1}H NMR (500 MHz, $CDCl_3$) δ 7.04 – 6.99 (m, 2H), 6.94 – 6.81 (m, 3H), 6.84 – 6.71 (m, 2H), 3.88 (s, 3H), 3.83 (s, 3H), 3.58 – 3.46 (m, 2H), 3.31 – 3.07 (m, 2H), 2.35 – 2.62 (m, 4H), 2.14 (ddd, *J* = 16.1, 8.9, 4.5 Hz, 1H), 2.04 – 1.93 (m, 1H), 1.75 – 1.63 (m, 1H). ^{13}C NMR (126 MHz, $CDCl_3$) δ 149.03, 148.63, 136.93, 136.77, 132.14, 131.77, 130.32, 128.81, 122.03, 119.13, 118.76, 111.16, 109.76, 55.91, 48.75, 47.85, 46.10, 44.60, 36.42, 28.36, 21.07.

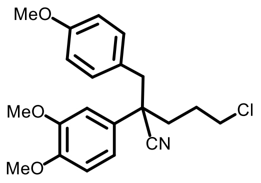

Int 2 for PhA39. Yellow oil, 21% yield. ^{1}H NMR (400 MHz, $CDCl_3$) δ 6.98 – 6.74 (m, 5H), 6.71 (dq, *J* = 4.1, 2.5, 2.0 Hz, 2H), 3.84 (s, 3H), 3.79 (s, 3H), 3.71 (s, 3H), 3.58 – 3.45 (m, 2H), 3.11 – 3.01 (m, 2H), 2.29 – 2.15 (m, 1H), 2.15 – 1.81 (m, 2H), 1.71 – 1.50 (m, 1H). ^{13}C NMR (101 MHz, $CDCl_3$) δ 149.47, 149.01, 148.78, 148.59, 131.40, 129.52, 126.87, 118.78, 113.47, 111.52, 111.15, 110.18, 109.69, 55.98, 55.16, 48.88, 47.43, 44.60, 38.46, 32.97, 28.34.

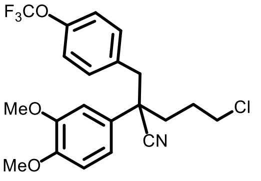

Int 2 for PhA40. Yellow oil, 34% yield. ^{1}H NMR (500 MHz, $CDCl_3$) δ 7.10 – 7.05 (m, 3H), 7.02 – 6.96 (m, 1H), 6.96 – 6.78 (m, 2H), 6.72 – 6.61 (m, 1H), 3.91 (s, 3H), 3.82 (s, 3H), 3.63 – 3.49 (m, 2H), 3.37 – 3.09 (m, 2H), 2.35 (ddd, *J* = 13.7, 11.9, 4.3 Hz, 1H), 2.23 – 2.11 (m, 1H), 2.08 – 1.96 (m, 1H), 1.71 (dddt, *J* = 14.0, 12.4, 9.5, 4.8 Hz, 1H). ^{13}C NMR (126 MHz, $CDCl_3$) δ 149.12, 148.80, 148.60, 133.48, 131.71, 131.68, 121.64, 120.55, 120.50, 118.81, 118.61, 111.24, 109.70, 55.95, 55.93, 48.71, 47.56, 44.50, 36.58, 29.70, 28.27.

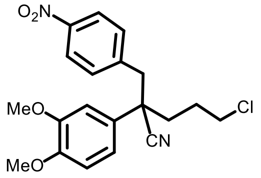

Int 2 for PhA41. Yellow oil, 36% yield. ^{1}H NMR (500 MHz, $CDCl_3$) δ 8.11 – 8.05 (m, 2H), 7.18 – 7.10 (m, 2H), 6.85 (d, *J* = 2.4 Hz, 2H), 6.77 (d, *J* = 1.8 Hz, 1H), 3.92 (s, 3H), 3.86 (s, 3H), 3.62 – 3.50 (m, 2H), 3.34 (d, *J* = 13.3 Hz, 1H), 3.23 (d, *J* = 13.4 Hz, 1H), 2.38 (ddd, *J* = 13.6, 11.8, 4.3 Hz, 1H), 2.22 (ddd, *J* = 14.0, 11.8, 4.8 Hz, 1H), 2.13 – 1.96 (m, 1H), 1.71 (dddd, *J* = 14.8, 9.2, 7.7, 4.8 Hz, 1H). ^{13}C NMR (126 MHz, $CDCl_3$) δ 149.37, 149.08, 147.52, 147.38, 142.21, 131.14, 128.20, 123.24, 118.85, 111.34, 111.13, 110.43, 109.34, 56.11, 55.95, 48.62, 44.36, 37.17, 29.69, 22.68.

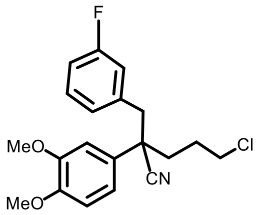

Int 2 for PhA42. Yellow oil, 29% yield. ^{1}H NMR (500 MHz, $CDCl_3$) δ 7.20 (td, *J* = 8.0, 6.0 Hz, 1H), 6.96 – 6.67 (m, 6H), 3.91 (s, 3H), 3.85 (s, 3H), 3.61 – 3.49 (m, 2H), 3.30 – 3.11 (m, 2H), 2.32 (ddd, *J* = 13.7, 11.8, 4.3 Hz, 1H), 2.22 – 2.11 (m, 1H), 2.08 – 1.94 (m, 1H), 1.70 (dddd, *J* = 19.4, 9.3, 8.1, 4.7 Hz, 1H). ^{13}C NMR (126 MHz, $CDCl_3$) δ 149.21, 148.86, 129.57, 128.99, 126.07, 121.64, 118.70, 117.29, 117.12, 114.43, 114.26, 111.29, 109.67, 56.05, 48.55, 47.86, 44.47, 36.71, 29.69, 28.27.

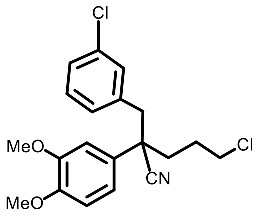

Int 2 for PhA6. Yellow oil, 31% yield. ^{1}H NMR (500 MHz, $CDCl_3$) δ 7.24 – 7.16 (m, 1H), 7.13 (t, *J* = 7.8 Hz, 1H), 7.02 (t, *J* = 1.9 Hz, 1H), 6.94 (dt, *J* = 5.9, 1.4 Hz, 1H), 6.90 – 6.78 (m, 2H), 6.73 (dd, *J* = 17.1, 2.1 Hz, 1H), 3.87 (s, 3H), 3.78 (s, 3H), 3.51 (tdd, *J* = 14.6, 9.7, 5.9 Hz, 2H), 3.29 – 3.20 (m, 1H), 3.16 – 3.06 (m, 1H), 2.30 (ddd, *J* = 13.5, 11.8, 4.3 Hz, 1H), 2.15 (ddd, *J* = 13.4, 11.5, 4.6 Hz, 1H), 1.98 (dtd, *J* = 18.2, 10.8, 10.3, 5.0 Hz, 1H), 1.74 – 1.64 (m, 1H). ^{13}C NMR (126 MHz, $CDCl_3$) δ 149.03, 148.96, 136.89, 133.86, 130.46, 129.42, 128.54, 127.60, 120.91, 119.10, 111.27, 110.53, 109.72, 55.99, 50.16, 48.52, 45.97, 44.46, 36.54, 28.31.

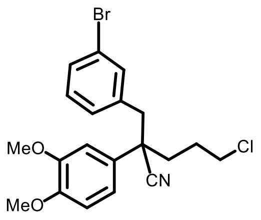

Int 2 for PhA43. Yellow oil, 34% yield. ^{1}H NMR (500 MHz, $CDCl_3$) δ 7.38 (dd, *J* = 8.2, 2.4 Hz, 1H), 7.24 – 7.15 (m, 1H), 7.14 – 7.04 (m, 1H), 7.00 (ddd, *J* = 11.0, 7.6, 5.2 Hz, 1H), 6.96 – 6.80 (m, 2H), 6.77 – 6.68 (m, 1H), 3.91 (s, 3H), 3.85 (s, 3H), 3.54 (tdd, *J* = 15.7, 7.7, 4.4 Hz, 2H), 3.37 – 3.08 (m, 2H), 2.32 (ddd, *J* = 13.7, 11.9, 4.4 Hz, 1H), 2.21 – 2.10 (m, 1H), 2.08 – 1.94 (m, 1H), 1.77 – 1.65 (m, 1H). ^{13}C NMR (126 MHz, $CDCl_3$) δ 149.24, 148.91, 137.04, 133.32, 130.43, 129.64, 128.96, 128.11, 127.26, 122.04, 118.79, 111.34, 109.70, 56.01, 48.54, 47.77, 44.48, 36.48, 29.70, 28.29.

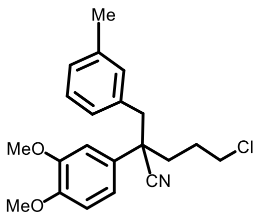

Int 2 for PhA44. Yellow oil, 50% yield. ^{1}H NMR (500 MHz, $CDCl_3$) δ 7.14 – 7.01 (m, 2H), 6.92 (ddt, *J* = 6.8, 5.1, 2.3 Hz, 2H), 6.88 – 6.72 (m, 3H), 3.89 (s, 3H), 3.84 (s, 3H), 3.52 (dtd, *J* = 13.0, 10.9, 5.2 Hz, 2H), 3.29 – 3.09 (m, 2H), 2.28 (s, 3H), 2.30 – 2.26 (m, 1H), 2.15 (ddd, *J* = 13.8, 11.6, 4.6 Hz, 1H), 1.99 (ddt, *J* = 13.6, 11.3, 5.4 Hz, 1H), 1.76 – 1.64 (m, 1H). ^{13}C NMR (126 MHz, $CDCl_3$) δ 149.03, 148.64, 137.58, 135.09, 131.34, 127.99, 127.97, 127.95, 127.48, 121.98, 119.12, 118.77, 111.14, 109.75, 55.99, 48.60, 48.21, 46.36, 44.61, 36.30, 28.35, 21.37.

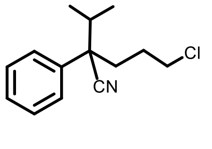

Int 2 for PhA45. Yellow oil, 68% yield. ^{1}H NMR (400 MHz, $CDCl_3$) δ 7.43 – 7.10 (m, 5H), 3.36 (dd, *J* = 6.9, 5.7 Hz, 1H), 2.26 – 1.89 (m, 3H), 1.75 (ddtd, *J* = 11.9, 9.0, 5.9, 4.4 Hz, 1H), 1.41 – 1.26 (m, 1H), 1.25 – 1.04 (m, 3H), 1.02 – 0.60 (m, 4H). ^{13}C NMR (101 MHz, $CDCl_3$) δ 137.79, 135.13, 128.98, 128.89, 128.02, 127.94, 127.88, 126.42, 120.95, 119.88, 60.33, 53.41, 45.01, 44.61, 37.87, 35.19, 33.81, 28.82, 21.05, 20.81, 18.96, 18.83, 18.61, 14.29.

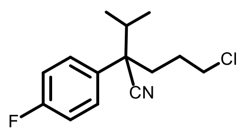

Int 2 for PhA46. Clear oil, 44% yield. ^{1}H NMR (400 MHz, $CDCl_3$) δ 7.37 (dd, J = 8.9, 5.0 Hz, 2H), 7.08 (d, J = 8.6 Hz, 2H), 3.55 – 3.42 (m, 2H), 2.33 – 2.10 (m, 2H), 2.10 – 1.98 (m, 2H), 1.92 – 1.77 (m, 1H), 1.04 (s, 3H), 0.79 (d, J = 6.7 Hz, 3H). ^{13}C NMR (101 MHz, $CDCl_3$) δ 163.28, 160.82, 133.44, 133.41, 129.56, 129.48, 128.11, 128.03, 120.80, 115.90, 115.87, 115.68, 115.65, 52.78, 44.47, 44.32, 38.01, 35.18, 33.81, 29.69, 28.61, 20.62, 18.77, 18.74, 18.52.

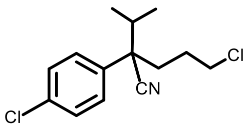

Int 2 for PhA47. Yellow oil, 58% yield. ^{1}H NMR (400 MHz, $CDCl_3$) δ 7.30 – 7.20 (m, 4H), 3.47 – 3.31 (m, 2H), 2.17 (ddd, *J* = 13.7, 11.9, 4.4 Hz, 1H), 2.12 – 1.88 (m, 2H), 1.81 – 1.67 (m, 1H), 1.30 (ddddd, *J* = 13.4, 11.6, 7.2, 5.7, 4.1 Hz, 1H), 1.23 – 1.04 (m, 4H), 0.99 – 0.61 (m, 4H). ^{13}C NMR (101 MHz, $CDCl_3$) δ 136.23, 133.52, 128.92, 128.13, 127.71, 120.37, 60.15, 57.37, 52.81, 44.28, 37.71, 34.89, 32.33, 28.50, 18.67, 18.61, 18.38, 17.40, 14.08.

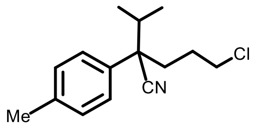

Int 2 for PhA48. Clear oil, 42% yield. ^{1}H NMR (400 MHz, $CDCl_3$) δ 7.16 (d, J = 8.3 Hz, 2H), 7.08 (d, J = 8.2 Hz, 2H), 3.47 – 3.29 (m, 2H), 2.24 (s, 3H), 2.17 – 1.98 (m, 2H), 1.97 – 1.65 (m, 2H), 1.40 – 1.26 (m, 1H), 1.11 (dd, J = 6.7, 1.4 Hz, 3H), 0.69 (d, J = 6.7 Hz, 3H). ^{13}C NMR (101 MHz, $CDCl_3$) δ 137.47, 134.59, 129.54, 126.23, 121.11, 52.99, 44.62, 37.85, 35.17, 28.72, 20.94, 18.90, 18.56.

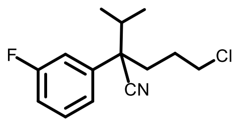

Int 2 for PhA49. Clear oil, 53% yield. ^{1}H NMR (400 MHz, $CDCl_3$) δ 7.31 – 7.25 (m, 1H), 7.11 (ddd, J = 7.9, 1.9, 1.0 Hz, 1H), 7.02 (dt, J = 10.1, 2.2 Hz, 1H), 6.94 (tdd, J = 8.3, 2.5, 1.0 Hz, 1H), 3.46 – 3.34 (m, 2H), 2.19 (ddd, J = 13.5, 11.9, 4.3 Hz, 1H), 2.08 – 1.99 (m, 1H), 1.95 (ddd, J = 13.5, 12.0, 4.3 Hz, 1H), 1.85 – 1.71 (m, 1H), 1.38 – 1.28 (m, 1H), 1.14 (d, J = 6.8 Hz, 3H), 0.72 (d, J = 6.7 Hz, 3H). ^{13}C NMR (101 MHz, $CDCl_3$) δ 164.25, 161.79, 130.56, 130.47, 122.15, 122.12, 120.46, 114.99, 114.79, 113.73, 113.50, 53.30, 53.29, 44.42, 37.94, 35.20, 28.59, 19.04, 18.87, 18.53.

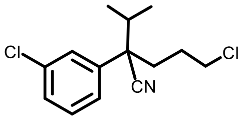

Int 2 for PhA50. Yellow oil, 55% yield. ^{1}H NMR (400 MHz, $CDCl_3$) δ 7.32 – 7.28 (m, 1H), 7.27 – 7.17 (m, 3H), 3.39 (td, J = 5.8, 5.4, 1.2 Hz, 2H), 2.24 – 2.13 (m, 1H), 2.09 – 1.99 (m, 1H), 1.95 (ddd, J = 13.6, 12.0, 4.4 Hz, 1H), 1.84 – 1.66 (m, 1H), 1.40 – 1.24 (m, 1H), 1.13 (d, J = 6.6 Hz, 3H), 0.71 (d, J = 6.8 Hz, 3H). ^{13}C NMR (101 MHz, $CDCl_3$) δ 139.98, 134.99, 130.21, 128.12, 126.56, 124.63, 120.33, 53.25, 44.40, 37.88, 35.10, 28.62, 18.90, 18.53.

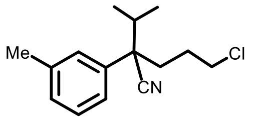

Int 2 for PhA51. Clear oil, 44% yield. ^{1}H NMR (400 MHz, $CDCl_3$) δ 7.18 – 7.14 (m, 1H), 7.11 (q, J = 1.3 Hz, 1H), 7.09 – 7.04 (m, 1H), 7.03 (dt, J = 1.8, 0.9 Hz, 1H), 3.37 (dd, J = 6.9, 5.6 Hz, 2H), 2.28 (s, 3H), 2.20 – 2.12 (m, 1H), 2.07 – 2.02 (m, 1H), 1.96 (ddd, J = 13.5, 11.9, 4.4 Hz, 1H), 1.82 – 1.70 (m, 1H), 1.40 – 1.27 (m, 1H), 1.13 (s, 3H), 0.70 (d, J = 6.7 Hz, 3H). ^{13}C NMR (101 MHz, $CDCl_3$) δ 138.58, 137.60, 128.74, 128.68, 128.66, 128.52, 128.49, 127.12, 123.24, 53.29, 44.64, 37.85, 35.22, 28.70, 21.59, 19.21, 18.98, 18.55.

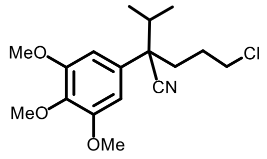

Int 2 for PhA52. Clear oil, 44% yield. ^{1}H NMR (500 MHz, $CDCl_3$) δ 6.62 (s, 2H), 4.18 – 3.69 (m, 9H), 3.62 – 3.44 (m, 2H), 2.26 (ddd, *J* = 13.6, 11.7, 4.5 Hz, 1H), 2.18 – 2.07 (m, 2H), 1.98 – 1.87 (m, 1H), 1.62 – 1.46 (m, 1H), 1.37 – 1.19 (m, 5H), 0.96 – 0.85 (m, 3H). ^{13}C NMR (126 MHz, $CDCl_3$) δ 203.24, 171.01, 153.30, 153.05, 137.29, 133.28, 131.38, 120.88, 105.76, 103.49, 77.48, 77.23, 76.97, 60.79, 60.77, 60.28, 56.21, 56.16, 53.49, 44.64, 38.01, 36.56, 34.96, 31.83, 31.35, 29.60, 29.56, 29.27, 28.50, 24.62, 23.35, 22.60, 20.93, 19.29, 18.95, 18.48, 14.12, 14.05.

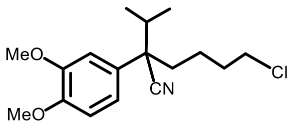

Int 2 for PhA58. Orange-yellow oil, 43% yield, ^{1}H NMR (400 MHz, $CDCl_3$) δ 6.82 (dd, J = 8Hz, 4Hz, 2H) 6.75 (d, J = 8Hz, 1H), 3.79 (s, 3H), 3.77 (s, 3H), 3.33 (t, J = 8Hz, 2H), 2.05-1.94 (m, 2H), 1.77-1.56 (m, 3H), 1.42-1.35 (m, 1H), 1.15 (t, J = 8Hz, 2H), 1.08 (d, J = 8Hz, 3H), 0.69 (d, J = 8Hz, 3H). ^{13}C NMR (101 MHz, $CDCl_3$) δ 149.03, 148.32, 130.29, 121.22, 118.74, 111.09, 109.45, 55.94, 55.80, 53.25, 44.25, 37.78, 36.98, 32.26, 22.98, 18.85, 18.49.

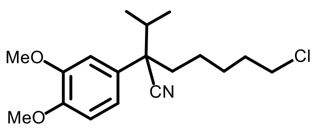

Int 2 for PhA59. Brown oil, 48% yield. ^{1}H NMR (400 MHz, $CDCl_3$) δ 6.84 (dd, J = 8.3, 2.2 Hz, 1H), 6.81 – 6.73 (m, 2H), 3.81 (s, 3H), 3.80 (s, 3H), 3.36 (t, J = 6.6 Hz, 2H), 2.02 (ddd, J = 13.4, 11.8, 8.4 Hz, 2H), 1.72 (dd, J = 4.5, 2.1 Hz, 1H), 1.59 (q, J = 7.0 Hz, 2H), 1.41 – 1.15 (m, 4H), 1.10 (d, J = 6.7 Hz, 3H), 0.71 (d, J = 6.7 Hz, 3H). ^{13}C NMR (101 MHz, $CDCl_3$) δ 148.96, 148.23, 130.53, 121.34, 118.66, 111.08, 109.49, 55.96, 55.82, 53.34, 44.76, 37.77, 37.63, 32.18, 26.71, 24.87, 18.88, 18.52.

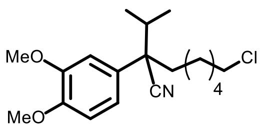

Int 2 for PhA60. Orange-yellow oil, 45% yield^{1}H NMR (400 MHz, $CDCl_3$) δ 6.81 (dd, J = 8Hz, 4Hz, 2H), 6.75 (d, J = 8Hz, 1H), 3.78 (s, 3H), 3.77 (s, 3H), 3.35 (t, J = 8Hz, 2H), 1.98-1.95 (m, 2H), 1.69- 1.67 (m, 1H) 1.57-1.55 (m, 2H), 1.32-1.1 (m, 6H), 1.07 (d, J = 8Hz, 3H), 0.68 (d, J = 8Hz, 3H). ^{13}C NMR (101 MHz, $CDCl_3$) δ 148.97, 148.23, 130.66, 121.40, 118.70, 111.07, 109.56, 55.95, 55.81, 53.36, 44.86, 37.74, 37.63, 32.31, 28.67, 26.44, 25.32, 18.88, 18.52.

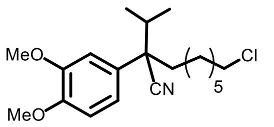

Int 2 for PhA61. Orange-yellow oil, 57% yield. ^{1}H NMR (400 MHz, $CDCl_3$) δ 6.81 (dd, J = 8.4, 2.2 Hz, 1H), 6.77 – 6.72 (m, 2H), 3.78 (s, 3H), 3.77 (s, 3H), 3.36 (t, J = 6.7 Hz, 2H), 2.03 – 1.90 (m, 2H), 1.71 – 1.52 (m, 3H), 1.30 – 1.11 (m, 8H), 1.07 (d, J = 6.7 Hz, 3H), 0.68 (d, J = 6.7 Hz, 3H). ^{13}C NMR (101 MHz, $CDCl_3$) δ 148.92, 148.18, 130.70, 121.45, 118.68, 111.03, 109.57, 55.96, 55.81, 53.37, 44.94, 37.76, 37.73, 32.44, 29.29, 28.48, 26.63, 25.40, 18.89, 18.53.

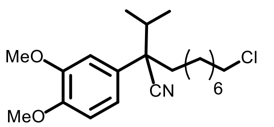

Int 2 for PhA62. Orange-yellow oil, 42% yield. ^{1}H NMR (400 MHz, $CDCl_3$) δ 6.91 (dd, J = 8Hz, 4Hz, 2H), 6.85 (d, J = 8Hz, 1H), 3.89 (s, 3H), 3.88 (s, 3H), 2.12-2.02 (m, 2H), 1.79-1.69 (m, 2H) 1.68- 1.58 (m, 2H), 1.47-1.23 (m, 9H), 1.18 (d, J = 8Hz, 3H), 1.03-0.92 (m, 2H) 0.78 (d, J = 8Hz, 3H). ^{13}C NMR (101 MHz, $CDCl_3$) δ 148.94, 148.21, 130.79, 130.76, 121.52, 118.70, 111.06, 109.64, 56.00, 55.85, 53.41, 45.06, 40.19, 37.82, 32.52, 29.68, 29.36, 26.72, 18.92, 18.57, 10.92.

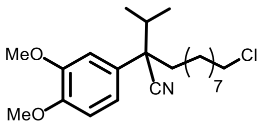

Int 2 for PhA63. Brown oil, 46% yield. ^{1}H NMR (400 MHz, $CDCl_3$) δ 6.80 – 6.60 (m, 3H), 3.97 – 3.48 (m, 8H), 3.31 (t, *J* = 6.7 Hz, 1H), 2.08 – 1.69 (m, 4H), 1.69 – 1.41 (m, 4H), 1.41 – 0.53 (m, 21H). ^{13}C NMR (101 MHz, $CDCl_3$) δ 170.41, 148.70, 147.95, 130.48, 130.45, 121.06, 118.47, 110.77, 109.28, 65.21, 59.86, 58.33, 58.31, 55.61, 55.45, 53.06, 44.61, 39.92, 39.90, 37.58, 37.41, 37.39, 32.24, 31.10, 31.08, 29.09, 29.03, 28.99, 28.90, 28.78, 28.75, 28.54, 28.43, 28.41, 28.39, 26.43, 26.17, 26.14, 25.97, 25.95, 25.14, 24.98, 20.55, 18.58, 18.21, 13.84, 10.54.

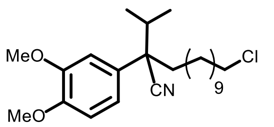

Int 2 for PhA64. Orange-yellow oil, 48% yield. ^{1}H NMR (400 MHz, $CDCl_3$) δ 6.88-6.74 (m, 3H), 4.00- 3.97 (m, 1H), 3.88 (d, J = 8Hz, 1H), 3.83 (s, 3H), 3.82 (s, 3H), 3.57 (t, J = 8Hz, 1H), 3.34 (t, J = 8Hz, 1H), 2.07-1.97 (m, 2H), 1.82-1.68 (m, 1H) 1.57-1.48 (m, 2H), 1.38- 1.12 (m, 15H), 0.99 (d, J = 8Hz, 3H), 0.92-0.72 (m, 2H). ^{13}C NMR (101 MHz, $CDCl_3$) δ 149.10, 148.68, 127.34, 120.21, 120.01, 111.20, 111.02, 110.81, 77.55, 77.23, 76.91, 62.75, 55.92, 55.87, 44.61, 37.76, 33.74, 29.40, 29.38, 29.36, 28.10, 25.73, 20.66, 18.86.

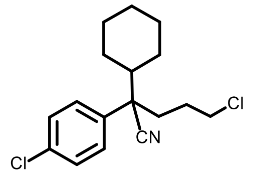

Int 2 for PhA14. Yellow oil, 52% yield. ^{1}H NMR (400 MHz, $CDCl_3$) δ 7.25 – 7.06 (m, 4H), 3.40 – 3.22 (m, 2H), 2.20 – 1.99 (m, 1H), 1.99 – 1.85 (m, 2H), 1.85 – 1.60 (m, 3H), 1.60 – 1.38 (m, 3H), 1.22 (tddd, *J* = 16.5, 14.4, 7.3, 5.4 Hz, 2H), 1.14 – 1.02 (m, 5H), 1.02 – 0.80 (m, 3H). ^{13}C NMR (101 MHz, $CDCl_3$) δ 136.21, 136.16, 133.63, 131.86, 129.73, 129.04, 128.88, 128.77, 128.24, 127.90, 126.27, 121.02, 119.81, 77.53, 77.21, 76.90, 60.36, 52.35, 47.22, 46.39, 45.58, 45.52, 44.52, 38.23, 34.53, 31.92, 30.70, 29.70, 29.66, 29.38, 29.36, 28.65, 28.61, 28.46, 27.29, 26.91, 26.23, 26.16, 25.93, 25.84, 25.79, 22.70, 21.03, 14.22, 14.15.

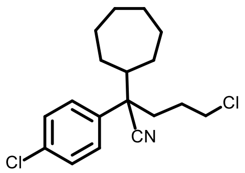

Int 2 for PhA65. Yellow oil, 50% yield. ^{1}H NMR (400 MHz, $CDCl_3$) δ 7.49 – 7.23 (m, 4H), 3.59 – 3.31 (m, 2H), 2.37 – 2.16 (m, 1H), 2.15 – 1.94 (m, 2H), 1.96 – 1.12 (m, 17H). ^{13}C NMR (101 MHz, $CDCl_3$) δ 202.88, 139.02, 136.76, 134.71, 133.62, 129.76, 129.07, 128.87, 128.80, 128.27, 127.95, 121.32, 119.81, 77.47, 77.15, 76.84, 52.85, 48.58, 46.59, 44.51, 35.12, 31.93, 30.75, 30.13, 30.01, 29.70, 29.36, 28.53, 28.34, 27.96, 27.42, 26.97, 26.74, 22.70, 14.15.

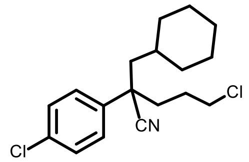

Int 2 for PhA15. Yellow oil, 68% yield. ^{1}H NMR (400 MHz, $CDCl_3$) δ 7.52 – 7.17 (m, 4H), 3.55 – 3.39 (m, 2H), 2.14 – 1.95 (m, 2H), 1.95 – 1.70 (m, 4H), 1.70 – 1.36 (m, 6H), 1.38 – 1.18 (m, 3H), 1.18 – 0.93 (m, 3H), 0.93 – 0.67 (m, 1H). ^{13}C NMR (101 MHz, $CDCl_3$) δ 136.85, 136.78, 133.70, 133.60, 129.77, 129.59, 129.13, 129.06, 128.86, 128.83, 127.96, 127.45, 122.16, 121.29, 77.54, 77.22, 76.90, 52.84, 48.54, 48.24, 46.12, 44.49, 44.43, 44.29, 39.64, 35.10, 34.84, 34.50, 34.23, 33.84, 33.40, 31.92, 30.75, 30.12, 30.01, 29.70, 29.36, 28.54, 28.34, 28.09, 27.96, 27.42, 27.11, 26.96, 26.73, 26.24, 26.15, 26.00, 25.96, 22.70, 21.02, 14.22.

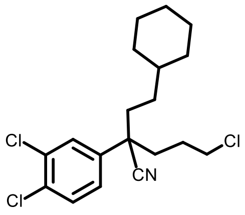

Int 2 for PhA11. Brown oil, 55% yield. ^{1}H NMR (400 MHz, $CDCl_3$) δ 7.32 – 7.14 (m, 3H), 4.47 – 4.11 (m, 7H), 4.04 – 3.74 (m, 3H), 2.48 – 2.19 (m, 6H), 2.16 – 1.80 (m, 8H), 1.80 – 1.05 (m, 12H). ^{13}C NMR (101 MHz, $CDCl_3$) δ 149.17, 148.44, 130.92, 130.32, 128.04, 125.25, 122.60, 122.33, 118.30, 111.21, 110.16, 109.95, 108.88, 60.26, 55.94, 55.87, 55.81, 47.35, 47.33, 44.45, 44.08, 42.54, 38.77, 38.47, 38.23, 38.01, 37.45, 37.39, 35.60, 34.81, 33.15, 33.10, 33.00, 32.93, 32.51, 32.14, 31.80, 29.61, 29.27, 28.28, 28.18, 26.51, 26.43, 26.22, 26.15, 26.12, 22.61, 20.93, 14.12.

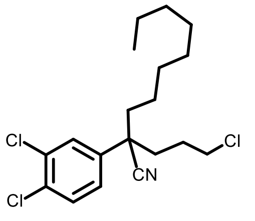

Int 2 for PhA12. Dark brown oil. 38% yield. ^{1}H NMR (400 MHz, $CDCl_3$) δ 7.51-7.41 (m, 2H) 7.27-7.24 (m, 1H), 3.54- 3.47 (m, 2H), 2.75-2.53 (m, 2H), 2.26-2.07 (m, 2H), 2.02-1.95 (m, 3H), 1.67-1.64 (m, 2H), 1.56-1.55 (m, 3H), 1.28-1.20 (m, 6H), 0.87-0.79 (m, 3H). ^{13}C NMR (101 MHz, $CDCl_3$) δ 140.71, 131.50, 130.95, 128.08, 125.34, 123.80, 117.20, 47.36, 39.74, 38.06, 31.74, 29.10, 26.39, 25.64, 25.17, 22.59, 17.65, 16.43, 14.07.

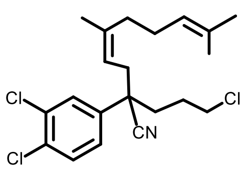

Int 2 for PhA13. Brown oil, 47% yield. ^{1}H NMR (400 MHz, $CDCl_3$) δ 7.58 – 7.37 (m, 2H), 7.34 – 7.19 (m, 1H), 5.19 – 4.84 (m, 2H), 3.65 – 3.41 (m, 1H), 2.84 – 2.50 (m, 2H), 2.34 – 1.77 (m, 7H), 1.77 – 1.37 (m, 12H), 1.37 – 1.16 (m, 4H), 1.10 – 0.73 (m, 2H). ^{13}C NMR (101 MHz, $CDCl_3$) δ 142.80, 140.79, 140.49, 139.06, 138.23, 133.33, 132.75, 132.20, 131.80, 131.61, 130.87, 130.39, 128.62, 128.51, 126.23, 126.00, 125.77, 123.90, 123.81, 121.69, 120.04, 117.74, 117.35, 117.19, 109.96, 58.84, 52.91, 47.90, 47.83, 45.46, 44.41, 44.27, 39.76, 39.30, 38.23, 38.10, 37.94, 35.04, 33.43, 31.94, 31.43, 31.14, 30.70, 30.20, 29.71, 29.67, 29.38, 28.56, 27.28, 26.89, 26.45, 26.41, 25.70, 25.66, 23.49, 22.71, 22.34, 18.85, 18.51, 17.67, 16.46, 16.34, 14.14.

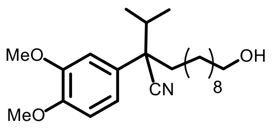

Int 2 for PhA9. Orange-yellow oil, 21% yield. ^{1}H NMR (400 MHz, $CDCl_3$) δ 6.86 (dd, J = 8 Hz, 4Hz, 1H), 6.80-6.78 (m, 2H) 3.83 (s, 3H), 3.81 (s, 3H), 3.55-3.51 (m, 2H), 2.39 (s, 1H) 2.02-1.96 (m, 2H), 1.71 (td, J = 8Hz, 4Hz, 1H), 1.51-1.43 (m, 2H), 1.23- 1.10 (m, 17H), 0.71 (d, J = 8Hz, 3H). ^{13}C NMR (101 MHz, $CDCl_3$) δ 148.87, 148.12, 130.74, 121.49, 118.71, 111.00, 109.55, 62.65, 55.90, 55.77, 53.36, 37.73, 32.67, 29.51, 29.46, 29.44, 29.42, 29.39, 29.37, 29.20, 25.71, 18.86, 18.50.

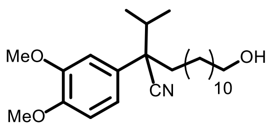

Int 2 for PhA10. Orange-yellow oil, 32% yield. ^{1}H NMR (400 MHz, $CDCl_3$) δ 6.84 (dd, J = 8 Hz, 4Hz, 1H), 6.79-6.76 (m, 2H) 3.82 (s, 3H), 3.81 (s, 3H), 3.53 (t, J = 8Hz, 2H), 2.04-1.94 (m, 3H), 1.69 (td, J = 8Hz, 4Hz, 1H), 1.50-1.43 (m, 2H), 1.26-1.09 (m, 21H), 0.71 (d, J = 8Hz, 3H). ^{13}C NMR (101 MHz, $CDCl_3$) δ 148.89, 148.14, 130.79, 121.55, 118.72, 111.00, 109.59, 62.84, 55.95, 55.82, 53.40, 37.80, 32.72, 29.66, 29.53, 29.51, 29.47, 29.42, 29.38, 29.32, 29.24, 25.72, 25.49, 18.90, 18.55.

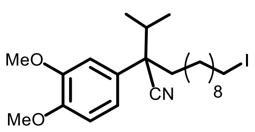

Int 3 for PhA9. Orange-yellow oil, 91% yield. ^{1}H NMR (400 MHz, $CDCl_3$) δ 6.91 (dd, J = 8 Hz, 4Hz, 1H), 6.84-6.82 (m, 2H) 3.88 (s, 3H), 3.87 (s, 3H), 3.16 (td, J = 8Hz, 4Hz, 2H), 2.11- 2.02 (m, 2H), 1.82-1.70 (m, 3H), 1.37-1.31 (m, 3H), 1.24-1.15 (m, 14H), 0.77 (d, J = 8Hz, 3H). ^{13}C NMR (101 MHz, $CDCl_3$) δ 148.91, 148.17, 130.81, 121.53, 118.73, 111.03, 109.64, 56.00, 55.87, 53.40, 37.81, 33.54, 30.46, 29.54, 29.46, 29.44, 29.35, 29.26, 28.49, 25.51, 18.94, 18.59, 7.31.

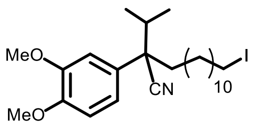

Int 3 for PhA10. Orange-yellow oil, 84% yield. ^{1}H NMR (400 MHz, $CDCl_3$) δ 6.84 (dd, J = 8 Hz, 4Hz, 1H), 6.79-6.77 (m, 2H) 3.82 (s, 3H), 3.81 (s, 3H), 3.10 (t, J = 8Hz, 2H), 2.05-1.97 (m, 2H), 1.77-1.62 (m, 3H), 1.33-1.26 (m, 3H), 1.21-1.10 (m, 18H), 0.72 (d, J = 8Hz, 3H). ^{13}C NMR (101 MHz, $CDCl_3$) δ 148.92, 148.17, 130.83, 121.58, 118.73, 111.02, 109.65, 56.01, 55.88, 53.42, 37.84, 33.55, 30.48, 29.69, 29.56, 29.48, 29.46, 29.45, 29.37, 29.28, 28.51, 25.53, 18.94, 18.59, 7.34.

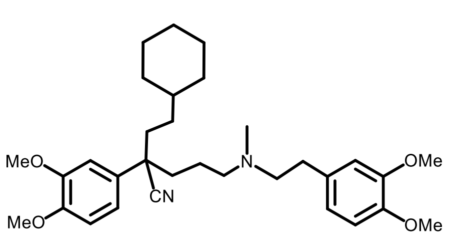

PhA1. Yellow oil, 69% yield. ^{1}H NMR (400 MHz, $CDCl_3$) δ 6.84 (dd, J = 8.3, 2.2 Hz, 1H), 6.79 (d, J = 2.2 Hz, 1H), 6.78 (s, 1H), 6.71 (d, J = 7.9 Hz, 1H), 6.62 (d, J = 7.7 Hz, 2H), 3.82 (s, 3H), 3.80 (s, 3H), 3.79 (s, 3H), 3.77 (s, 3H), 2.69 – 2.57 (m, 2H), 2.55 – 2.42 (m, 2H), 2.34 (td, J = 7.1, 2.0 Hz, 2H), 2.17 (s, 3H), 1.96 – 1.85 (m, 2H), 1.85 – 1.72 (m, 2H), 1.66 – 1.14 (m, 15H). ^{13}C NMR (101 MHz, $CDCl_3$) δ 149.10, 148.86, 148.34, 147.38, 132.61, 130.91, 122.73, 120.51, 118.23, 112.03, 111.27, 111.22, 109.12, 59.28, 56.67, 56.02, 55.91, 55.88, 55.85, 47.83, 41.76, 38.86, 38.70, 37.57, 33.20, 33.01, 32.95, 32.62, 29.69, 26.51, 26.23, 26.20, 22.79. ESI-MS: calcd for $C_{32}H_{46}N_2O_4$ =522.3, found [M+H]+ =523.3.

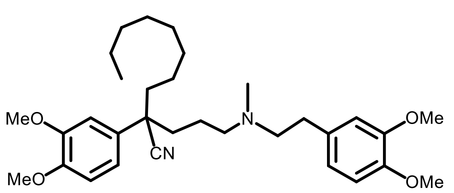

PhA2. Yellow oil, 55% yield. ^{1}H NMR (400 MHz, $CDCl_3$) δ 6.91 (dd, J = 8.4, 2.0 Hz, 1H), 6.86 (d, J = 2.1 Hz, 1H), 6.83 (dd, J = 8.4, 1.3 Hz, 1H), 6.80 – 6.75 (m, 1H), 6.69 (d, J = 9.2 Hz, 2H), 3.88 (s, 3H), 3.86 (s, 3H), 3.85 (s, 3H), 3.83 (s, 3H), 2.67 (dd, J = 9.8, 5.5 Hz, 2H), 2.53 (dd, J = 9.6, 6.2 Hz, 2H), 2.44 – 2.28 (m, 2H), 2.21 (s, 3H), 1.93 (ddd, J = 16.4, 6.5, 3.3 Hz, 2H), 1.90 – 1.76 (m, 2H), 1.68 – 1.18 (m, 12H), 0.90 – 0.79 (m, 3H). ^{13}C NMR (101 MHz, $CDCl_3$) δ 149.08, 148.81, 148.32, 147.31, 132.88, 130.98, 122.75, 120.50, 118.19, 112.03, 111.25, 111.22, 109.16, 59.40, 56.78, 55.99, 55.89, 55.87, 55.82, 47.81, 41.91, 41.28, 38.77, 33.14, 31.76, 29.41, 29.22, 29.13, 25.22, 22.97, 22.58, 14.07. ESI-MS: calcd for $C_{32}H_{48}N_2O_4$ =524.3, found [M+H]+ =525.5.

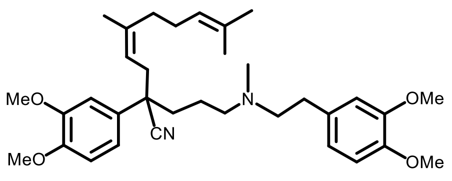

PhA3. Yellow oil, 61% yield. ^{1}H NMR (400 MHz, $CDCl_3$) δ 6.84 (dd, J = 8.3, 2.2 Hz, 1H), 6.80 (d, J = 2.2 Hz, 1H), 6.74 (d, J = 8.4 Hz, 1H), 6.69 (d, J = 7.9 Hz, 1H), 6.61 (d, J = 8.0 Hz, 2H), 4.98 (dt, J = 7.7, 3.8 Hz, 1H), 4.93 (qd, J = 4.9, 4.4, 2.3 Hz, 1H), 3.79 (s, 3H), 3.77 (s, 3H), 3.76 (s, 3H), 3.74 (s, 3H), 2.64 – 2.58 (m, 2H), 2.58 – 2.47 (m, 2H), 2.47 – 2.42 (m, 2H), 2.31 (dt, J = 6.7, 3.4 Hz, 2H), 2.14 (s, 3H), 1.89 (dq, J = 11.9, 6.8 Hz, 6H), 1.56 (d, J = 1.4 Hz, 3H), 1.47 (d, J = 1.3 Hz, 3H), 1.46 (d, J = 1.3 Hz, 3H), 1.38 – 1.18 (m, 2H). ^{13}C NMR (101 MHz, $CDCl_3$) δ 148.94, 148.80, 148.34, 147.29, 139.89, 132.86, 131.35, 130.83, 123.92, 122.63, 120.50, 118.43, 117.81, 112.03, 111.27, 111.12, 109.51, 59.35, 56.79, 55.95, 55.86, 55.83, 55.79, 47.83, 41.90, 39.79, 39.73, 37.46, 33.10, 26.44, 25.64, 22.95, 17.64, 16.40. ESI-MS: calcd for $C_{34}H_{48}N_2O_4$ =548.3, found [M+H]+ =549.7.

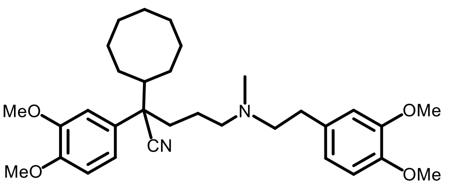

PhA4. Yellow oil, 60% yield. ^{1}H NMR (400 MHz, $CDCl_3$) δ 6.86 (dd, J = 8.4, 2.2 Hz, 1H), 6.81 (d, J = 2.2 Hz, 1H), 6.76 (d, J = 8.3 Hz, 1H), 6.73 – 6.68 (m, 1H), 6.61 (d, J = 7.0 Hz, 2H), 3.82 (s, 3H), 3.81 (s, 3H), 3.79 (s, 3H), 3.78 (s, 3H), 2.60 (dd, J = 8.5, 2.0 Hz, 2H), 2.50 – 2.41 (m, 2H), 2.35 – 2.24 (m, 2H), 2.13 (s, 3H), 1.97 – 1.81 (m, 2H), 1.74 (dt, J = 7.6, 2.5 Hz, 1H), 1.60 – 1.16 (m, 16H). ^{13}C NMR (101 MHz, $CDCl_3$) δ 148.85, 148.19, 131.07, 120.49, 118.88, 112.01, 111.25, 111.07, 109.82, 59.37, 56.79, 56.04, 55.92, 55.86, 54.25, 45.93, 41.86, 35.70, 32.99, 29.85, 29.59, 26.99, 26.67, 26.07, 25.75. ESI-MS: calcd for C32H46N2O4 = 522.3, found [M+H]+ = 523.3.

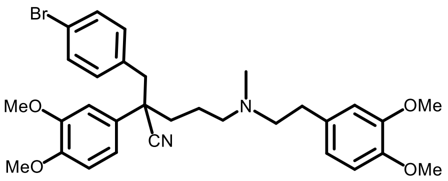

PhA5. Yellow oil, 10% yield. ^{1}H NMR (400 MHz, $CDCl_3$) δ 7.36 – 7.31 (m, 2H), 6.90 – 6.79 (m, 5H), 6.76 – 6.70 (m, 3H), 3.92 – 3.85 (m, 12H), 3.15 – 3.00 (m, 2H), 2.72 (dd, *J* = 9.1, 5.6 Hz, 2H), 2.58 (t, *J* = 7.9 Hz, 2H), 2.44 (qd, *J* = 12.1, 11.4, 5.4 Hz, 2H), 2.27 (s, 3H), 2.14 – 1.99 (m, 2H), 1.69 (td, *J* = 12.3, 6.1 Hz, 1H), 1.40 (dd, *J* = 12.5, 6.5 Hz, 1H). ^{13}C NMR (126 MHz, $CDCl_3$) δ 148.99, 148.87, 148.60, 147.39, 134.05, 131.95 (2C), 131.15, 130.35, 129.47, 128.02, 121.93, 121.42, 120.56, 118.59, 112.11, 111.29, 111.15, 109.85, 59.40, 56.74, 56.01, 55.94, 55.91, 55.89, 49.08, 47.44, 41.94, 37.32, 33.15, 23.03. ESI-MS: calcd for $C_{31}H_{37}BrN_2O_4$ = 580.2, found [M+H]+ = 581.2.

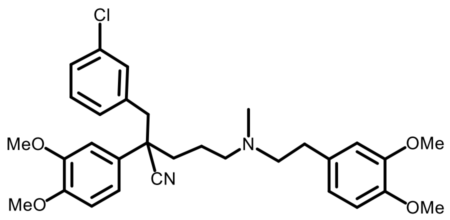

PhA6. Yellow oil, 50% yield. ^{1}H NMR (400 MHz, $CDCl_3$) δ 7.20 (ddd, *J* = 8.1, 2.1, 1.1 Hz, 1H), 7.13 (t, *J* = 7.8 Hz, 1H), 6.93 (t, *J* = 1.9 Hz, 1H), 6.88 – 6.66 (m, 7H), 3.93 – 3.82 (m, 12H), 3.15 (d, *J* = 13.4 Hz, 1H), 3.04 (d, *J* = 13.3 Hz, 1H), 2.97 – 2.65 (m, 3H), 2.57 (dd, *J* = 9.1, 6.7 Hz, 1H), 2.44 – 2.37 (m, 2H), 2.26 (s, 3H), 2.07 (dddd, *J* = 30.2, 13.7, 11.7, 4.7 Hz, 2H), 1.74 – 1.62 (m, 1H), 1.41 (tq, *J* = 12.0, 5.2 Hz, 1H). ^{13}C NMR (126 MHz, $CDCl_3$) δ 149.07, 148.88, 148.71, 147.39, 137.06, 133.76, 130.38, 129.54, 129.25, 128.48, 127.43, 121.87, 120.57 (2C), 118.60, 112.14, 111.32, 111.27, 109.97, 59.40, 56.74, 56.06, 55.97, 55.94, 55.88, 48.95, 47.59, 41.96, 37.21, 33.19, 23.05. ESI-MS: calcd for $C_{31}H_{37}ClN_2O_4$ = 536.2, found [M+H]+ = 537.2.

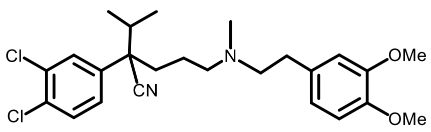

PhA7. Yellow oil, 23% yield. ^{1}H NMR (400 MHz, $CDCl_3$) δ 7.38 (d, J = 2.3 Hz, 1H), 7.35 (d, J = 8.4 Hz, 1H), 7.10 (dd, J = 8.5, 2.3 Hz, 1H), 6.71 (d, J = 8.7 Hz, 1H), 6.61 (d, J = 6.9 Hz, 2H), 3.78 (s, 3H), 3.75 (s, 3H), 2.58 (dd, J = 9.5, 6.6 Hz, 2H), 2.46 – 2.38 (m, 2H), 2.32 – 2.19 (m, 2H), 2.10 (s, 3H), 2.03 (ddd, J = 13.6, 12.1, 4.4 Hz, 1H), 1.98 – 1.90 (m, 1H), 1.71 (ddd, J = 13.8, 12.2, 4.4 Hz, 1H), 1.52 – 1.38 (m, 1H), 1.09 (d, J = 6.6 Hz, 3H), 0.69 (d, J = 6.7 Hz, 3H). ^{13}C NMR (101 MHz, $CDCl_3$) δ 148.80, 147.30, 138.83, 133.05, 131.79, 130.65, 128.45, 125.88, 120.55, 120.40, 112.10, 111.29, 59.35, 56.73, 55.90, 55.84, 53.29, 41.96, 37.82, 35.27, 33.22, 23.49, 18.88, 18.51. ESI-MS: calcd for $C_{25}H_{32}Cl_2N_2O_2$ = 462.2, found [M+H]+ = 463.1.

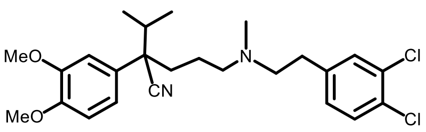

PhA8. Yellow oil, 56% yield. ^{1}H NMR (400 MHz, $CDCl_3$) δ 7.26 (d, J = 4Hz, 1H), 7.20 (s, 1H), 6.94-6.75 (m, 4H), 3.81 (s, 3H), 3.80 (s, 3H), 2.63 (t, J = 8Hz, 2H), 2.45 (t, J = 8Hz, 2H), 2.33- 2.26 (m, 2H), 2.11 (s, 3H), 2.03-1.96 (m, 2H) 1.79- 1.67 (m, 1H), 1.51-1.40 (m, 1H), 1.18-1.17 (m, 1H), 1.10 (d, J = 8Hz, 3H), 0.71 (2, J = 8Hz, 3H). ^{13}C NMR (101 MHz, $CDCl_3$) δ 149.01, 148.30, 137.86, 132.12, 130.67, 130.58, 130.23, 128.22, 121.43, 118.65, 113.84, 111.08, 109.57, 58.42, 56.76, 56.01, 55.89, 53.34, 41.60, 37.95, 35.44, 32.43, 23.13, 18.96, 18.60. ESI-MS: calcd for $C_{25}H_{32}Cl_2N_2O_2$ = 462.2, found [M+H]+ = 463.1.

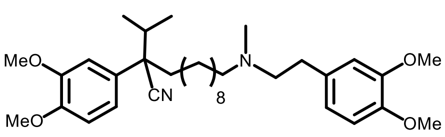

PhA9. Yellow oil, 54% yield. ^{1}H NMR (400 MHz, $CDCl_3$) δ 6.86-6.75 (m, 6H), 3.90 (s, 3H), 3.88 (s, 3H), 3.87 (s, 3H), 3.85 (s. 3H), 2.78 (t, J = 8Hz, 2H), 2.67 (t, J = 8Hz, 2H), 2.47 (t, J = 8Hz, 2H) 2.36 (s, 3H), 2.12-2.02 (m, 2H), 1.76 (td, J = 8Hz, 4Hz, 1H), 1.26-1.17 (m, 19H), 0.79 (d, J = 8Hz, 3H). ^{13}C NMR (101 MHz, $CDCl_3$) δ 148.91, 148.88, 148.17, 147.41, 132.56, 130.83, 121.58, 120.51, 118.73, 112.05, 111.32, 111.03, 109.65, 76.79, 59.50, 57.55, 55.99, 55.92, 55.85, 53.42, 41.89, 37.84, 32.99, 29.56, 29.52, 29.49, 29.47, 29.43, 29.29, 29.27, 27.47, 26.79, 25.53, 18.92, 18.58. ESI-MS: calcd for $C_{34}H_{52}N_2O_4$ = 552.4, found [M+H]+ = 553.1.

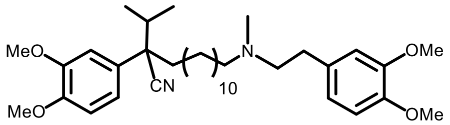

PhA10. Yellow oil, 60% yield. ^{1}H NMR (400 MHz, CDCl$_3$) δ 6.79-6.68 (m, 6H), 3.82 (s, 3H), 3.81 (s, 3H), 3.80 (s, 3H), 3.79 (s. 3H) 2.87-2.76 (m, 4H), 2.58 (t, J = 8Hz, 2H) 2.44 (s, 3H), 2.02-1.97 (m, 2H), 1.69 (td, J = 8Hz, 4Hz, 1H), 1.38-1.17 (m, 23H), 0.72 (d, J = 8Hz, 3H). ^{13}C NMR (101 MHz, CDCl$_3$) δ 149.05, 148.91, 148.18, 147.73, 130.83, 121.60, 120.54, 118.73, 112.03, 111.40, 111.02, 109.66, 109.63, 58.77, 57.45, 57.08, 56.01, 55.94, 55.92, 55.87, 53.43, 41.20, 37.86, 32.10, 32.03, 32.00, 29.69, 29.56, 29.47, 29.34, 29.29, 27.23, 25.66, 25.54, 18.92, 18.58. ESI-MS: calcd for $C_{36}H_{56}N_2O_4$ = 580.4, found [M+H]+ = 581.4.

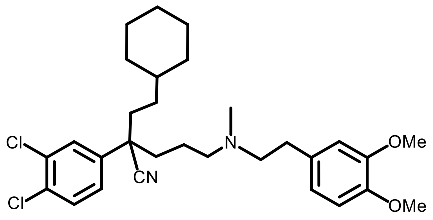

PhA11. Yellow oil, 61% yield. ^{1}H NMR (400 MHz, CDCl$_3$) δ 7.49 – 7.35 (m, 2H), 7.16 (dd, *J* = 8.4, 2.3 Hz, 1H), 6.81 – 6.62 (m, 3H), 3.81 (d, *J* = 8.0 Hz, 6H), 2.63 (dd, *J* = 9.6, 6.0 Hz, 2H), 2.48 (dd, *J* = 9.4, 6.2 Hz, 2H), 2.31 (td, *J* = 6.7, 2.9 Hz, 2H), 2.17 (s, 3H), 2.03 – 1.70 (m, 4H), 1.70 – 1.51 (m, 6H), 1.38 – 0.68 (m, 9H). ^{13}C NMR (101 MHz, CDCl$_3$) δ 149.09, 147.59, 139.46, 133.41, 133.30, 132.14, 131.09, 128.37, 125.68, 121.94, 120.83, 112.38, 111.56, 59.66, 56.93, 56.18, 56.10, 48.06, 42.25, 38.79, 38.75, 37.76, 33.53, 33.35, 33.25, 32.86, 26.73, 26.46, 26.43, 23.41. ESI-MS: calcd for $C_{30}H_{40}Cl_2N_2O_2$ = 530.3, found [M+H]+ = 531.3.

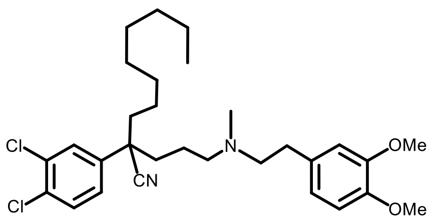

PhA12. Yellow oil, 63% yield. ^{1}H NMR (400 MHz, CDCl$_3$) δ 7.48 – 7.37 (m, 2H), 7.18 (dd, *J* = 8.4, 2.3 Hz, 1H), 6.82 – 6.72 (m, 1H), 6.72 – 6.62 (m, 2H), 3.83 (d, *J* = 7.8 Hz, 6H), 2.65 (dd, *J* = 9.9, 6.3 Hz, 2H), 2.55 – 2.44 (m, 2H), 2.33 (td, *J* = 6.7, 3.4 Hz, 2H), 2.01 – 1.67 (m, 4H), 1.64 – 1.54 (m, 1H), 1.49 – 1.35 (m, 1H), 1.29 – 1.14 (m, 12H), 1.09 – 0.95 (m, 1H), 0.83 (t, *J* = 6.9 Hz, 3H). ^{13}C NMR (101 MHz, CDCl$_3$) δ 149.13, 147.64, 139.47, 133.48, 133.30, 132.22, 131.13, 128.38, 125.75, 122.00, 120.87, 112.39, 111.57, 59.71, 56.97, 56.22, 56.15, 48.08, 42.29, 41.34, 38.77, 33.58, 32.06, 29.64, 29.49, 29.43, 25.53, 23.44, 22.91, 14.41. ESI-MS: calcd for $C_{30}H_{42}Cl_2N_2O_2$ = 532.3, found [M+H]+ = 534.

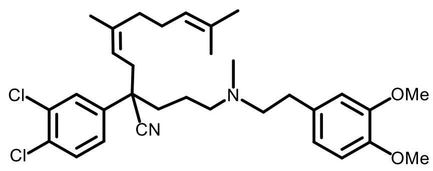

PhA13. Yellow oil, 59% yield. ^{1}H NMR (400 MHz, CDCl$_3$) δ 7.49 – 7.37 (m, 1H), 7.20 (dd, *J* = 8.4, 2.3 Hz, 1H), 6.78 (d, *J* = 8.7 Hz, 1H), 6.73 – 6.65 (m, 1H), 4.99 (ddtt, *J* = 5.7, 4.3, 2.8, 1.4 Hz, 1H), 3.84 (d, *J* = 7.8 Hz, 3H), 2.70 – 2.58 (m, 2H), 2.58 – 2.45 (m, 2H), 2.45 – 2.30 (m, 1H), 2.21 (s, 2H), 2.07 – 1.81 (m, 3H), 1.73 – 1.46 (m, 5H), 1.37 – 1.22 (m, 1H). ^{13}C NMR (101 MHz, CDCl$_3$) δ 148.88, 147.39, 141.00, 138.95, 133.04, 132.98, 131.95, 131.70, 130.67, 128.39, 125.77, 123.85, 121.69, 120.60, 116.99, 112.11, 111.31, 59.49, 56.76, 55.96, 55.89, 47.90, 42.01, 39.79, 39.63, 37.27, 33.30, 26.45, 25.75, 23.17, 17.75, 16.47. ESI-MS: calcd for $C_{32}H_{42}Cl_2N_2O_2$ = 556.3, found [M+H]+ = 557.9.

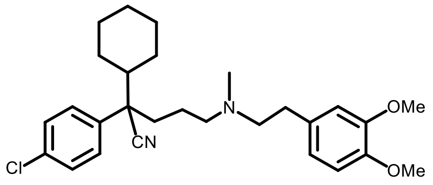

PhA14. Yellow oil, 60% yield. ^{1}H NMR (400 MHz, CDCl$_3$) δ 7.34 – 7.14 (m, 4H), 6.80 – 6.55 (m, 3H), 3.78 (d, *J* = 6.2 Hz, 6H), 2.59 (dd, *J* = 9.5, 6.2 Hz, 2H), 2.42 (ddd, *J* = 7.9, 6.3, 3.3 Hz, 2H), 2.37 – 2.16 (m, 2H), 2.16 – 1.94 (m, 5H), 1.86 – 1.65 (m, 2H), 1.65 – 1.35 (m, 4H), 1.35 – 0.71 (m, 11H). ^{13}C NMR (101 MHz, CDCl$_3$) δ 149.24, 147.75, 137.14, 133.79, 133.37, 129.28, 128.31, 121.81, 120.95, 112.47, 111.66, 59.64, 57.20, 56.33, 56.24, 53.14, 47.62, 42.38, 35.23, 33.50, 31.84, 30.60, 30.10, 29.08, 29.04, 26.66, 26.59, 26.27, 23.55. ESI-MS: calcd for $C_{28}H_{37}ClN_2O_2$ = 468.3, found [M+H]+ = 469.2.

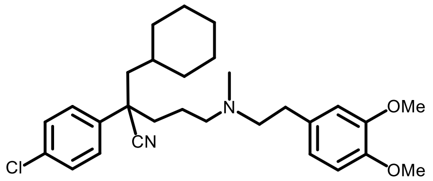

PhA15. Yellow oil, 52% yield. ^{1}H NMR (400 MHz, CDCl$_3$) δ 7.39 – 7.27 (m, 4H), 6.87 – 6.62 (m, 3H), 3.86 (d, *J* = 6.2 Hz, 6H), 2.67 (dd, *J* = 9.9, 6.3 Hz, 2H), 2.58 – 2.44 (m, 2H), 2.35 (tt, *J* = 8.0, 4.3 Hz, 2H), 2.21 (d, *J* = 5.3 Hz, 3H), 2.00 – 1.72 (m, 5H), 1.72 – 1.44 (m, 5H), 1.41 – 0.88 (m, 7H), 0.76 (dtd, *J* = 13.3, 11.7, 3.3 Hz, 1H). ^{13}C NMR (101 MHz, CDCl$_3$) δ 149.17, 147.69, 137.68, 133.79, 133.22, 129.32, 129.24, 128.31, 127.81, 122.88, 120.91, 112.42, 111.61, 59.56, 56.98, 56.26, 56.19, 53.59, 48.83, 48.52, 46.83, 42.30, 42.26, 40.48, 35.75, 35.20, 34.60, 34.18, 33.45, 30.49, 28.29, 27.76, 27.32, 27.10, 26.35, 26.34, 26.30, 23.55, 23.06. ESI-MS: calcd for $C_{29}H_{39}ClN_2O_2$ = 482.3, found [M+H]+ = 483.2

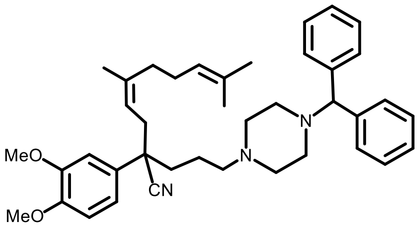

PhA16. Brown oil, 54% yield. ^{1}H NMR (400 MHz, CDCl$_3$) δ 7.37 – 7.23 (m, 3H), 7.23 – 7.14 (m, 3H), 7.14 – 6.95 (m, 2H), 6.87 – 6.68 (m, 3H), 5.06 – 4.87 (m, 1H), 4.12 (s, 1H), 3.88 – 3.72 (m, 6H), 2.66 – 2.13 (m, 8H), 2.04 – 1.71 (m, 5H), 1.71 – 1.36 (m, 7H), 1.36 – 0.97 (m, 7H), 0.97 – 0.58 (m, 2H). ^{13}C NMR (101 MHz, CDCl$_3$) δ 148.99, 148.39, 142.67, 140.03, 131.50, 130.85, 128.46, 127.91, 127.89, 126.92, 123.96, 122.63, 118.43, 117.81, 111.15, 109.54, 57.79, 56.07, 56.00, 55.91, 53.40, 53.29, 51.70, 47.84, 39.78, 37.52, 29.71, 26.51, 25.69, 22.49, 18.93, 18.59, 17.70, 16.46. ESI-MS: calcd for $C_{40}H_{51}N_3O_2$ = 605.4, found [M+H]+ = 606.4.

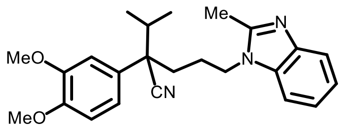

PhA17. Brown oil, 47% yield. ^{1}H NMR (400 MHz, $CDCl_3$) δ 7.73 – 7.54 (m, 1H), 7.25 – 7.07 (m, 3H), 6.66 (dd, *J* = 62.9, 1.3 Hz, 3H), 4.32 – 3.95 (m, 3H), 3.75 (d, *J* = 63.9 Hz, 6H), 2.49 (s, 3H), 2.17 (ddd, *J* = 13.3, 11.4, 4.8 Hz, 1H), 2.01 – 1.79 (m, 2H), 1.74 – 1.50 (m, 3H), 1.50 – 1.35 (m, 1H), 1.32 – 1.15 (m, 2H), 1.11 (d, *J* = 6.6 Hz, 3H), 0.95 – 0.66 (m, 4H). ^{13}C NMR (101 MHz, $CDCl_3$) δ 149.20, 148.50, 134.57, 130.92, 129.60, 128.81, 122.48, 122.30, 120.99, 118.62, 118.57, 111.13, 109.26, 108.88, 65.55, 55.89, 55.87, 53.00, 43.27, 38.00, 34.61, 30.55, 29.67, 25.63, 19.16, 18.86, 18.52, 13.70. ESI-MS: calcd for C24H29N3O3 = 391.2, found [M+H]+ = 392.5.

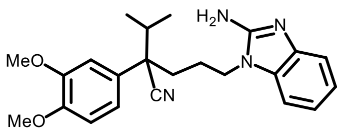

PhA18. Brown oil, 45% yield. ^{1}H NMR (400 MHz, $CDCl_3$) δ 7.35 – 6.47 (m, 6H), 4.88 (s, 2H), 3.94 – 3.45 (m, 7H), 2.22 – 1.97 (m, 1H), 1.88 (hept, *J* = 6.6 Hz, 1H), 1.83 – 1.58 (m, 2H), 1.58 – 1.37 (m, 1H), 1.18 (s, 1H), 1.03 (d, *J* = 6.6 Hz, 3H), 0.66 (d, *J* = 6.7 Hz, 3H). ^{13}C NMR (101 MHz, $CDCl_3$) δ 149.19, 148.49, 140.15, 133.38, 129.65, 121.84, 121.28, 120.24, 118.72, 115.71, 111.13, 109.08, 107.87, 55.93, 55.88, 53.01, 42.15, 38.02, 34.45, 24.95, 18.85, 18.54. ESI-MS: calcd for $C_{23}H_{28}N_4O_2$ = 392.2, found [M+H]+ = 393.4

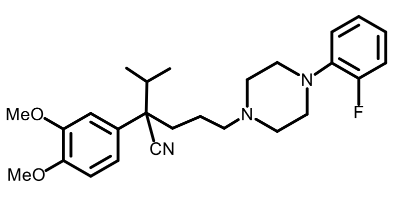

A1. Yellow oil, 45% yield. ^{1}H NMR (400 MHz, $CDCl_3$) δ 7.05 – 6.76 (m, 7H), 3.87 (d, *J* = 5.5 Hz, 6H), 3.04 (dt, *J* = 6.5, 3.1 Hz, 4H), 2.49 (t, *J* = 4.5 Hz, 4H), 2.33 (d, *J* = 7.2 Hz, 2H), 2.23 – 2.00 (m, 2H), 1.85 (ddd, *J* = 13.5, 12.2, 4.4 Hz, 1H), 1.71 – 1.50 (m, 1H), 1.18 (d, *J* = 6.6 Hz, 4H), 0.78 (d, *J* = 6.8 Hz, 3H). ^{13}C NMR (101 MHz, $CDCl_3$) □ 156.92, 154.48, 149.05, 148.32, 140.13, 140.05, 130.66, 124.45, 124.42, 122.43, 122.36, 121.38, 118.89, 118.86, 118.71, 116.18, 115.97, 111.14, 109.57, 77.44, 77.12, 76.80, 57.94, 56.01, 55.91, 53.39, 53.16, 50.46, 50.42, 37.88, 35.65, 22.93, 18.99, 18.63. ESI-MS: calcd for $C_{26}H_{34}FN_3O_2$ = 439.6, found [M+H]+ = 440.3.

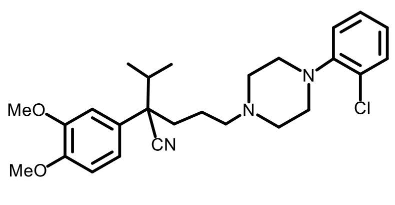

PhA19. Yellow oil, 47% yield. ^{1}H NMR (500 MHz, $CDCl_3$) δ 7.39 – 7.17 (m, 2H), 7.10 – 6.83 (m, 5H), 3.91 (d, *J* = 6.9 Hz, 6H), 3.05 (s, 4H), 2.54 (s, 3H), 2.38 (t, *J* = 7.2 Hz, 2H), 2.26 – 2.05 (m, 2H), 1.89 (ddd, *J* = 13.7, 12.2, 4.4 Hz, 1H), 1.70 – 1.54 (m, 1H), 1.22 (d, *J* = 6.7 Hz, 4H), 0.82 (d, *J* = 6.8 Hz, 3H). ^{13}C NMR (126 MHz, $CDCl_3$) δ 149.25, 149.04, 148.31, 130.66, 130.63, 128.73, 127.57, 123.63, 121.39, 120.31, 118.71, 111.13, 109.56, 77.34, 77.09, 76.84, 57.97, 56.01, 55.92, 53.45, 53.40, 53.27, 51.12, 37.89, 35.68, 22.99, 19.00, 18.64. ESI-MS: calcd for $C_{26}H_{34}ClN_3O_2$ = 456, found [M+H]+ = 457.4.

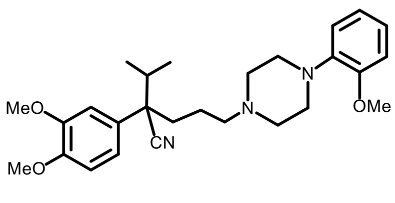

PhA20. Yellow oil, 61% yield. ^{1}H NMR (500 MHz, $CDCl_3$) δ 7.02 – 6.78 (m, 7H), 3.97 – 3.79 (m, 9H), 3.03 (s, 4H), 2.52 (s, 4H), 2.35 (t, *J* = 7.3 Hz, 2H), 2.19 – 2.05 (m, 2H), 1.90 – 1.79 (m, 1H), 1.60 (dt, *J* = 12.9, 7.1 Hz, 1H), 1.33 – 1.11 (m, 4H), 0.89 – 0.70 (m, 3H). ^{13}C NMR (126 MHz, $CDCl_3$) δ 152.24, 149.04, 148.29, 141.28, 130.70, 122.90, 121.38, 120.96, 118.74, 118.13, 111.15, 111.12, 109.47, 77.39, 77.14, 76.88, 58.00, 56.00, 55.92, 55.33, 53.42, 53.30, 50.55, 37.87, 35.69, 22.90, 19.00, 18.64. ESI-MS: calcd for $C_{27}H_{37}N_3O_3$ = 451.6, found [M+H]+ = 452.3.

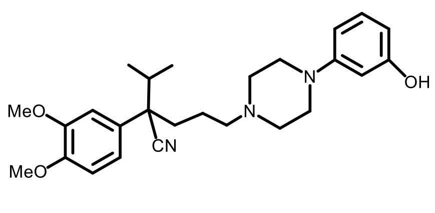

PhA21. Brown oil, 37% yield ^{1}H NMR (400 MHz, $CDCl_3$) δ 7.08 – 6.76 (m, 3H), 6.44 – 6.22 (m, 3H), 3.86 (d, *J* = 2.6 Hz, 5H), 3.08 (t, *J* = 5.2 Hz, 3H), 2.56 – 2.26 (m, 5H), 2.26 – 1.97 (m, 2H), 1.85 (td, *J* = 12.8, 4.4 Hz, 1H), 1.58 (dq, *J* = 14.3, 7.0 Hz, 1H), 1.32 – 1.00 (m, 4H), 0.77 (d, *J* = 6.7 Hz, 3H). ^{13}C NMR (101 MHz, $CDCl_3$) δ 157.15, 152.57, 149.03, 148.31, 130.46, 129.93, 121.46, 118.75, 111.17, 109.54, 108.25, 107.12, 103.41, 77.43, 77.11, 76.79, 57.86, 56.03, 55.91, 53.37, 52.87, 48.73, 37.89, 35.55, 22.68, 18.96, 18.60. ESI-MS: calcd for $C_{26}H_{35}N_3O_3$ = 437.6, found [M+H]+ = 438.3.

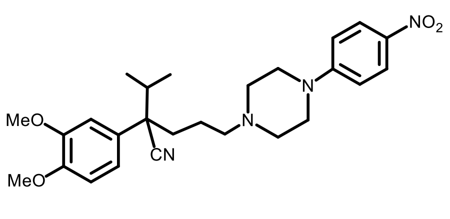

PhA22. Yellow oil, 42% yield. ^{1}H NMR (500 MHz, $CDCl_3$) δ 8.16 – 8.08 (m, 2H), 6.96 – 6.79 (m, 4H), 3.91 (d, *J* = 4.3 Hz, 6H), 3.53 – 3.35 (m, 4H), 2.58 – 2.30 (m, 6H), 2.30 – 2.04 (m, 2H), 1.88 (td, *J* = 13.1, 4.4 Hz, 1H), 1.61 (dq, *J* = 17.0, 5.6 Hz, 1H), 1.36 – 1.10 (m, 4H), 0.82 (d, *J* = 6.7 Hz, 3H). ^{13}C NMR (126 MHz, $CDCl_3$) δ 154.80, 149.07, 148.37, 138.43, 130.59, 125.93, 121.38, 118.69, 112.61, 111.15, 77.32, 77.06, 76.81, 57.69, 56.07, 55.94, 53.40, 52.51, 46.91, 37.92, 35.54, 22.89, 18.98, 18.63. ESI-MS: calcd for $C_{26}H_{34}N_4O_4$ = 466.6, found [M+H]+ = 468.2.

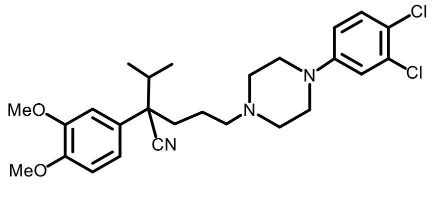

PhA23. Yellow oil, 45% yield. ^{1}H NMR (400 MHz, $CDCl_3$) δ 7.21 (d, *J* = 8.9 Hz, 1H), 6.95 – 6.80 (m, 4H), 6.67 (dd, *J* = 9.0, 2.9 Hz, 1H), 3.87 (d, *J* = 4.3 Hz, 6H), 3.13 – 2.98 (m, 4H), 2.51 – 2.24 (m, 6H), 2.24 – 2.01 (m, 2H), 1.84 (ddd, *J* = 13.6, 12.1, 4.4 Hz, 1H), 1.63 – 1.52 (m, 1H), 1.17 (d, *J* = 6.6 Hz, 4H), 0.77 (d, *J* = 6.7 Hz, 3H). ^{13}C NMR (101 MHz, $CDCl_3$) δ 150.64, 149.05, 148.34, 132.70, 130.63, 130.39, 121.95, 121.38, 118.70, 117.06, 115.21, 111.15, 109.58, 77.45, 77.13, 76.81, 57.75, 56.04, 55.92, 53.39, 52.73, 48.52, 37.87, 35.57, 22.92, 18.99, 18.63. ESI-MS: calcd for $C_{26}H_{37}Cl_2N_3O_2$ = 490.5, found [M]+ = 490.2.

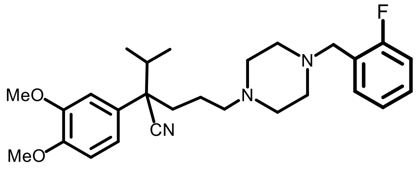

PhA24. Yellow oil, 57% yield. ^{1}H NMR (400 MHz, $CDCl_3$) δ 7.38 – 6.73 (m, 7H), 3.85 (d, *J* = 2.5 Hz, 6H), 3.55 (d, *J* = 1.5 Hz, 2H), 2.56 – 1.74 (m, 13H), 1.52 (tdd, *J* = 13.8, 7.2, 3.7 Hz, 1H), 1.16 (d, *J* = 6.7 Hz, 4H), 0.76 (d, *J* = 6.7 Hz, 3H). ^{13}C NMR (101 MHz, $CDCl_3$) δ 162.62, 160.18, 149.02, 148.28, 131.67, 131.62, 130.65, 128.81, 128.73, 124.48, 124.33, 123.82, 123.78, 121.36, 118.71, 115.32, 115.10, 111.10, 109.49, 77.43, 77.11, 76.79, 57.85, 55.97, 55.89, 55.16, 53.37, 52.97, 52.62, 37.84, 35.64, 22.89, 18.97, 18.61. ESI-MS: calcd for $C_{27}H_{36}FN_3O_2$ = 453.6, found [M+H]+ = 454.3.

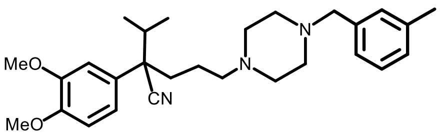

PhA25. Yellow oil, 40% yield. ^{1}H NMR (400 MHz, $CDCl_3$) δ 7.16 – 6.72 (m, 7H), 3.81 (d, *J* = 3.1 Hz, 6H), 3.39 (d, *J* = 6.4 Hz, 4H), 2.47 – 2.15 (m, 12H), 2.12 – 1.92 (m, 3H), 1.76 (td, *J* = 13.4, 12.9, 4.4 Hz, 2H), 1.47 (dt, *J* = 11.6, 6.0 Hz, 1H), 1.24 – 1.01 (m, 5H), 0.79 – 0.66 (m, 3H). ^{13}C NMR (101 MHz, $CDCl_3$) δ 148.30, 130.63, 129.99, 128.08, 127.85, 126.37, 118.71, 111.12, 63.02, 57.89, 56.00, 55.91, 53.37, 52.89, 37.87, 35.66, 22.85, 21.37, 18.97, 18.61. ESI-MS: calcd for $C_{28}H_{39}N_3O_2$ = 449.6, found [M+H]+ = 450.3.

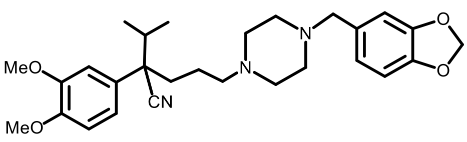

PhA26. Yellow oil, 33% yield. ^{1}H NMR (400 MHz, $CDCl_3$) δ 6.92 – 6.57 (m, 6H), 5.87 (s, 2H), 3.83 (d, *J* = 4.2 Hz, 6H), 3.34 (s, 2H), 2.24 (t, *J* = 7.3 Hz, 10H), 2.14 – 1.96 (m, 2H), 1.79 (ddd, *J* = 13.5, 12.1, 4.4 Hz, 1H), 1.51 (tdd, *J* = 14.5, 7.3, 4.0 Hz, 1H), 1.14 (d, *J* = 6.6 Hz, 4H), 0.74 (d, *J* = 6.7 Hz, 3H). ^{13}C NMR (101 MHz, $CDCl_3$) δ 149.01, 148.27, 147.57, 146.55, 131.88, 130.64, 122.23, 121.35, 118.70, 111.11, 109.51, 107.79, 100.83, 77.49, 77.17, 76.85, 62.71, 57.89, 55.97, 55.88, 53.35, 53.00, 52.80, 37.81, 35.64, 22.90, 18.97, 18.60. ESI-MS: calcd for $C_{28}H_{37}N_3O_4$ = 479.6, found [M+H]+ = 480.2.

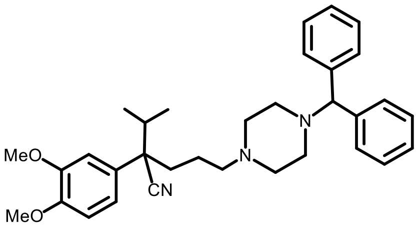

PhA27. Yellow oil, 28% yield. ^{1}H NMR (400 MHz, $CDCl_3$) δ 7.50 – 7.12 (m, 9H), 7.01 – 6.75 (m, 2H), 4.21 (s, 1H), 3.88 (d, *J* = 3.7 Hz, 5H), 3.02 – 2.80 (m, 1H), 2.52 – 2.19 (m, 8H), 2.19 – 1.99 (m, 2H), 1.85 (ddd, *J* = 13.6, 12.2, 4.4 Hz, 1H), 1.57 (tdd, *J* = 12.0, 6.7, 3.6 Hz, 1H), 1.36 – 1.11 (m, 4H), 1.03 – 0.72 (m, 3H). ^{13}C NMR (101 MHz, $CDCl_3$) δ 149.01, 148.28, 142.74, 130.67, 128.45, 127.93, 126.90, 121.39, 118.66, 111.11, 109.57, 77.40, 77.09, 76.77, 76.25, 57.92, 55.98, 55.89, 53.38, 53.32, 51.82, 37.85, 35.69, 22.87, 18.98, 18.62. ESI-MS: calcd for $C_{33}H_{41}N_3O_2$ = 511.7, found [M+H]+ = 512.2.

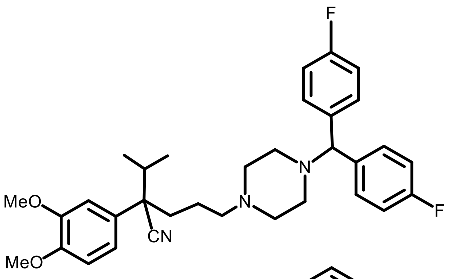

PhA28. Yellow oil, 23% yield. ^{1}H NMR (400 MHz, $CDCl_3$) δ 7.50 – 7.14 (m, 6H), 7.10 – 6.76 (m, 7H), 4.22 (d, *J* = 16.0 Hz, 1H), 3.87 (d, *J* = 3.0 Hz, 6H), 3.00 – 2.78 (m, 3H), 2.60 – 2.22 (m, 10H), 2.22 – 1.98 (m, 3H), 1.83 (td, *J* = 12.8, 4.6 Hz, 1H), 1.66 – 1.44 (m, 1H), 1.19 (d, *J* = 6.7 Hz, 4H), 0.79 (d, *J* = 6.7 Hz, 3H). ^{13}C NMR (101 MHz, $CDCl_3$) δ 149.01, 148.28, 138.22, 130.63, 129.28, 129.20, 121.37, 118.66, 115.45, 115.24, 111.10, 109.55, 77.39, 77.07, 76.76, 74.47, 57.86, 55.98, 55.89, 53.37, 53.24, 51.63, 37.84, 35.66, 22.86, 18.97, 18.61. ESI-MS: calcd for $C_{33}H_{39}F_2N_3O_2$ = 547.7, found [M+H]+ = 548.1.

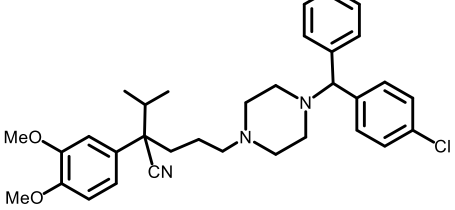

PhA29. Yellow oil, 32% yield. ^{1}H NMR (400 MHz, $CDCl_3$) δ 7.49 – 6.61 (m, 12H), 3.90 – 3.59 (m, 6H), 2.72 (s, 9H), 2.22 – 1.79 (m, 3H), 1.54 (d, *J* = 5.9 Hz, 1H), 1.37 – 0.88 (m, 6H), 0.71 (d, *J* = 6.7 Hz, 4H). ^{13}C NMR (101 MHz, $CDCl_3$) δ 129.20, 129.12, 128.73, 121.30, 118.74, 115.65, 115.43, 111.15, 109.50, 56.09, 55.89, 53.32, 37.93, 29.70, 18.92, 18.63. ESI-MS: calcd for $C_{33}H_{40}ClN_3O_2$ = 546.2, found [M]+ = 546.1.

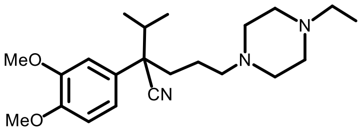

PhA30. Yellow oil, 43% yield. ^{1}H NMR (400 MHz, $CDCl_3$) δ 6.99 – 6.82 (m, 3H), 3.90 (d, *J* = 2.7 Hz, 6H), 3.49 (s, 2H), 2.47 (q, *J* = 7.2 Hz, 5H), 2.19 – 1.97 (m, 4H), 1.84 (ddd, *J* = 13.5, 12.1, 4.4 Hz, 1H), 1.60 – 1.50 (m, 1H), 1.28 – 1.14 (m, 5H), 1.17 – 0.77 (m, 7H). ^{13}C NMR (101 MHz, $CDCl_3$) δ 162.07, 150.33, 131.06, 128.50, 117.59, 116.76, 110.44, 109.56, 57.85, 55.91, 53.38, 52.52, 52.27, 37.87, 35.66, 25.31, 18.97, 18.61, 13.49, 11.64. ESI-MS: calcd for $C_{22}H_{35}N_3O_2$ = 373.5, found [M+H]+ = 374.3.

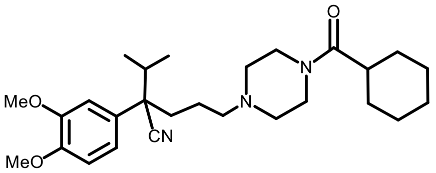

PhA31. Brown oil, 29% yield. ^{1}H NMR (400 MHz, $CDCl_3$) δ 6.89 – 6.74 (m, 3H), 3.82 (d, J = 3.6 Hz, 6H), 3.63 – 3.34 (m, 4H), 2.92 – 2.74 (m, 2H), 2.43 – 2.12 (m, 7H), 2.12 – 1.90 (m, 2H), 1.90 – 1.54 (m, 7H), 1.54 – 1.36 (m, 3H), 1.28 – 1.01 (m, 8H), 0.73 (d, *J* = 6.7 Hz, 3H). ^{13}C NMR (101 MHz, $CDCl_3$) δ 174.50, 149.08, 148.37, 118.66, 111.14, 109.58, 57.68, 56.05, 55.92, 53.36, 40.35, 37.91, 35.49, 29.37, 25.83, 18.96, 18.62. ESI-MS: calcd for $C_{27}H_{41}N_3O_3$ = 455.3, found [M+H]+ = 456.2.

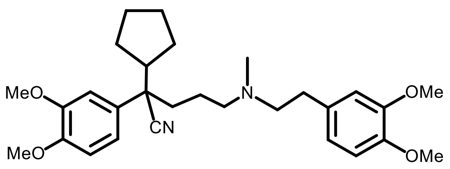

PhA32. Yellow oil, 52% yield. ^{1}H NMR (500 MHz, $CDCl_3$) δ 6.95 (dd, *J* = 8.3, 2.2 Hz, 1H), 6.89 (d, *J* = 2.3 Hz, 1H), 6.83 (d, *J* = 8.4 Hz, 1H), 6.78 (d, *J* = 7.9 Hz, 1H), 6.70 (s, 2H), 3.89 – 3.84 (dd, *J* = 12.3, 8.2 Hz, 12H), 2.74 – 2.60 (m, 2H), 2.56 – 2.47 (m, 2H), 2.43 – 2.22 (m, 3H), 2.19 (s, 3H), 2.02 – 1.93 (m, 2H), 1.89 (ddd, *J* = 13.7, 12.1, 4.5 Hz, 1H), 1.82 – 1.72 (m, 1H), 1.68 – 1.51 (m, 4H), 1.51 – 1.39 (m, 1H), 1.37 – 1.13 (m, 3H). ^{13}C NMR (126 MHz, $CDCl_3$) δ 148.99, 148.82, 148.23, 147.32, 132.90, 131.36, 121.82, 120.51, 118.41, 112.03, 111.24, 111.12, 109.38, 59.39, 56.82, 56.00, 55.90, 55.87, 55.84, 52.60, 50.08, 41.92, 37.23, 33.13, 29.55, 29.22, 25.49, 24.85, 23.04. ESI-MS: calcd for $C_{29}H_{40}N_2O_4$ = 480.3, found [M+H]+ = 481.2.

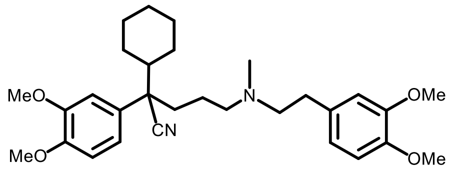

A2. Yellow oil, 58% yield. ^{1}H NMR (500 MHz, $CDCl_3$) δ 6.92 – 6.83 (m, 2H), 6.85 – 6.76 (m, 2H), 6.69 (d, *J* = 6.8 Hz, 2H), 3.87 (dd, *J* = 11.5, 7.5 Hz, 12H), 2.72 – 2.63 (m, 2H), 2.55 – 2.49 (m, 2H), 2.41 – 2.31 (m, 2H), 2.19 (s, 3H), 2.15 – 2.03 (m, 2H), 1.89 – 1.80 (m, 2H), 1.70 – 1.61 (m, 3H), 1.60 – 1.47 (m, 1H), 1.35 – 0.98 (m, 7H). ^{13}C NMR (126 MHz, $CDCl_3$) δ 148.97, 148.82, 148.21, 147.31, 133.00, 130.52, 121.95, 120.50, 118.72, 112.04, 111.24, 111.08, 109.73, 59.41, 56.90, 56.00, 55.90, 55.86, 55.83, 52.72, 47.32, 41.98, 35.00, 33.17, 28.74, 28.71, 26.32, 26.29, 25.95, 23.20. ESI-MS: calcd for $C_{30}H_{42}N_2O_4$ = 494.3, found [M+H]+ = 495.2.

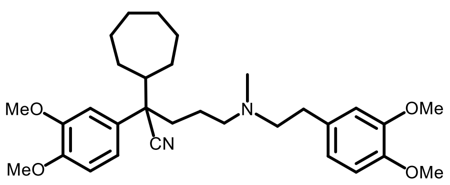

PhA33. Yellow oil, 57% yield. ^{1}H NMR (500 MHz, $CDCl_3$) δ 6.91 (dt, *J* = 8.3, 2.2 Hz, 1H), 6.87 (d, *J* = 2.2 Hz, 1H), 6.83 (d, *J* = 8.4 Hz, 1H), 6.78 (d, *J* = 7.8 Hz, 1H), 6.70 (s, 2H), 3.87 (ddd, *J* = 13.7, 7.7, 2.1 Hz, 12H), 2.73 – 2.66 (m, 2H), 2.54 (dd, *J* = 9.4, 6.6 Hz, 2H), 2.46 – 2.33 (m, 2H), 2.22 (s, 3H), 2.14 (tdd, *J* = 13.9, 4.5, 2.3 Hz, 1H), 2.09 – 2.01 (m, 1H), 1.92 – 1.81 (m, 2H), 1.80 – 1.69 (m, 1H), 1.65 – 1.41 (m, 8H), 1.32 – 1.10 (m, 4H). ^{13}C NMR (126 MHz, $CDCl_3$) δ 149.01, 148.86, 148.22, 147.38, 131.00, 122.20 (2C), 120.51, 118.85, 112.03, 111.28, 111.09, 109.76, 59.26, 56.76, 56.05, 55.91, 55.86, 53.26, 48.62, 45.85, 41.79, 35.57, 32.87, 30.24, 29.98, 28.08, 27.44, 27.16, 26.78, 23.03. ESI-MS: calcd for $C_{31}H_{44}N_2O_4$ = 508.3, found [M+H]+ = 509.2.

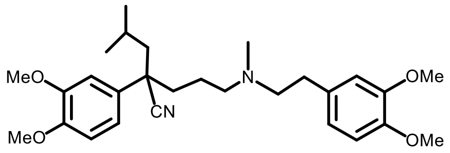

PhA34. Yellow oil, 55% yield. ^{1}H NMR (500 MHz, $CDCl_3$) δ 6.92 (dd, J = 8.4, 2.3 Hz, 1H), 6.85 (d, J = 2.3 Hz, 1H), 6.81 (d, J = 8.4 Hz, 1H), 6.75 (d, J = 8.0 Hz, 1H), 6.67 (s, 2H), 3.88 – 3.79 (m, 12H), 2.64 (dd, J = 10.2, 6.6 Hz, 2H), 2.52 – 2.45 (m, 2H), 2.32 (td, J = 6.7, 3.9 Hz, 2H), 2.17 (s, 3H), 1.87 – 1.74 (m, 4H), 1.68 – 1.53 (m, 2H), 1.28 – 1.17 (m, 1H), 0.96 (d, J = 6.6 Hz, 3H), 0.66 (d, J = 6.7 Hz, 3H). ^{13}C NMR (126 MHz, $CDCl_3$) δ 149.07, 148.81, 148.33, 147.30, 133.00, 130.91, 123.08, 120.52, 118.43, 112.06, 111.25, 111.19, 109.31, 59.42, 56.78, 56.02, 55.90, 55.87, 55.83, 49.60, 46.68, 41.95, 40.31, 33.22, 25.65, 23.94, 23.35, 22.80. ESI-MS: calcd for $C_{28}H_{40}N_2O_4$ = 468.3, found [M+H]+ = 469.2.

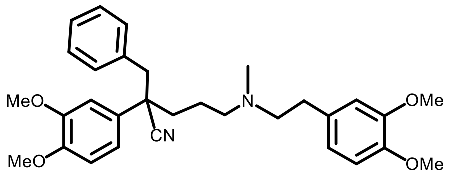

PhA35. Yellow oil, 33% yield. ^{1}H NMR (500 MHz, $CDCl_3$) δ 7.18 (dt, *J* = 5.1, 2.6 Hz, 3H), 6.98 – 6.91 (m, 2H), 6.86 – 6.73 (m, 3H), 6.69 (d, *J* = 4.5 Hz, 3H), 3.91 – 3.76 (m, 12H), 3.16 (d, *J* = 13.4 Hz, 1H), 3.05 (d, *J* = 13.5 Hz, 1H), 2.72 – 2.64 (m, 2H), 2.55 (dd, *J* = 9.0, 6.8 Hz, 2H), 2.48 – 2.40 (m, 2H), 2.24 (s, 3H), 2.13 – 1.98 (m, 2H), 1.72 – 1.60 (m, 1H), 1.39 (td, *J* = 11.8, 5.2 Hz, 1H). ^{13}C NMR (126 MHz, $CDCl_3$) δ 148.84, 148.45, 147.34, 135.05, 132.77, 130.33 (2C), 129.90, 127.98, 127.16 (2C), 122.17, 120.54, 118.54, 112.06, 111.88, 111.27, 111.08, 109.98, 59.33, 56.74, 55.90, 55.84, 49.11, 48.05, 41.89, 37.10, 36.45, 33.06, 31.39, 22.95. ESI-MS: calcd for $C_{31}H_{38}N_2O_4$ = 502.3, found [M+H]+ = 503.2.

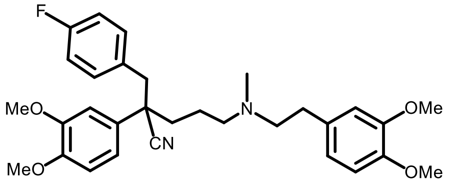

PhA36. Yellow oil, 21% yield. ^{1}H NMR (500 MHz, $CDCl_3$) δ 6.93 – 6.62 (m, 10H), 3.90 – 3.80 (m, 12H), 3.18 – 2.63 (m, 7H), 2.55 (t, *J* = 7.9 Hz, 1H), 2.48 – 2.35 (m, 1H), 2.16 (s, 3H), 2.14 – 1.97 (m, 1H), 1.71 – 1.59 (m, 1H), 1.44 – 1.32 (m, 1H). ^{13}C NMR (126 MHz, $CDCl_3$) δ 13, 148.94, 148.82, 148.52, 131.83, 131.76, 122.03, 120.81, 120.67, 120.54, 118.58, 114.94, 114.77, 112.08, 111.94, 111.88, 111.49, 111.26, 111.12, 59.40, 56.76, 55.89, 55.85, 51.41, 47.22, 41.96, 37.26, 34.44, 30.91, 29.79, 23.08. ESI-MS: calcd for $C_{31}H_{37}FN_2O_4$ = 520.3, found [M+H]+ = 521.2

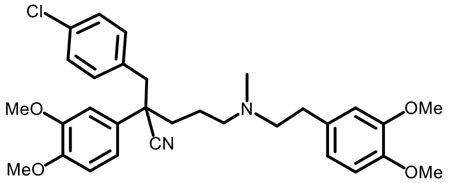

PhA37. Yellow oil, 23% yield. ^{1}H NMR (400 MHz, $CDCl_3$) δ 7.19 – 7.11 (m, 2H), 6.90 – 6.83 (m, 2H), 6.87 – 6.73 (m, 3H), 6.73 – 6.67 (m, 3H), 3.89 – 3.78 (m, 12H), 3.14 (d, *J* = 13.5 Hz, 1H), 3.02 (d, *J* = 13.5 Hz, 1H), 2.71 (dd, *J* = 9.2, 5.6 Hz, 2H), 2.63 – 2.54 (m, 2H), 2.45 (q, *J* = 7.1 Hz, 2H), 2.26 (s, 3H), 2.18 – 1.96 (m, 2H), 1.68 (tt, *J* = 12.6, 6.1 Hz, 1H), 1.40 (dt, *J* = 13.2, 5.8 Hz, 1H). ^{13}C NMR (126 MHz, $CDCl_3$) δ 148.98, 148.85, 148.58, 147.36, 133.56, 133.23, 131.59, 130.25, 129.51, 128.17, 121.95, 120.81, 120.67, 120.55, 118.59, 112.10, 111.27, 111.14, 109.84, 59.42, 56.76, 55.99, 55.90, 49.16, 47.36, 41.98, 37.33, 34.47, 33.21, 29.82, 23.08. ESI-MS: calcd for $C_{31}H_{37}ClN_2O_4$ = 536.2, found [M+H]+ = 537.2.

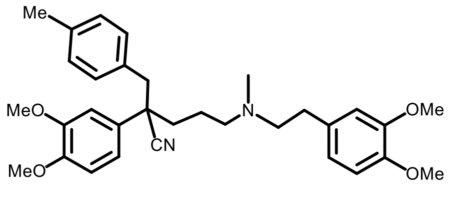

PhA38. Yellow oil, 16% yield. ^{1}H NMR (400 MHz, $CDCl_3$) δ 6.94 (d, *J* = 7.8 Hz, 2H), 6.80 (dq, *J* = 8.3, 2.2 Hz, 3H), 6.77 – 6.68 (m, 3H), 6.66 – 6.59 (m, 2H), 3.83 – 3.76 (m, 12H), 3.10 – 2.94 (m, 2H), 2.71 (d, *J* = 9.0 Hz, 2H), 2.63 (d, *J* = 8.1 Hz, 2H), 2.56 – 2.44 (m, 2H), 2.28 (s, 3H), 2.21 (s, 3H), 2.17 – 2.06 (m, 1H), 1.96 (ddd, *J* = 13.8, 11.6, 4.6 Hz, 1H), 1.68 – 1.60 (m, 1H), 1.47 – 1.38 (m, 1H). ^{13}C NMR (126 MHz, $CDCl_3$) δ 148.87, 148.81, 148.44, 147.36, 136.81, 132.90, 132.00, 130.23 (2C), 130.15, 128.73 (2C), 120.81, 120.54, 118.57, 112.08, 111.28, 111.07, 110.01, 59.45, 56.84, 55.94 (2C), 55.89, 55.87, 49.20, 47.68, 41.98, 37.14, 33.21, 23.05, 21.06. ESI-MS: calcd for $C_{32}H_{40}N_2O_4$ = 516.3, found [M+H]+ = 517.2.

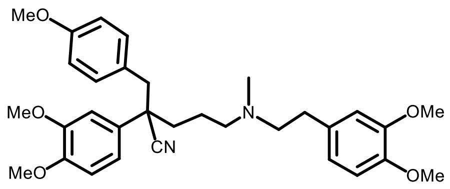

PhA39. Yellow oil, 22% yield. ^{1}H NMR (400 MHz, $CDCl_3$) □ 6.86 – 6.59 (m, 10H), 3.86 – 3.70 (m, 12H), 3.68 (s, 3H), 3.09 – 2.93 (m, 2H), 2.80 – 2.63 (m, 4H), 2.63 – 2.50 (m, 1H), 2.33 (s, 3H), 2.22 – 2.11 (m, 1H), 2.03 – 1.80 (m, 2H), 1.75 – 1.64 (m, 1H), 1.50 – 1.40 (m, 1H). ^{13}C NMR (126 MHz, $CDCl_3$) □ 149.34, 148.82, 148.41, 147.31, 133.01, 131.35, 130.11, 128.28, 127.16, 122.30, 120.54, 119.58, 118.61, 113.38, 112.08, 112.04, 111.27, 110.23, 109.97, 59.43, 56.84, 55.95, 55.90, 55.13, 49.35, 47.26, 42.01, 37.07, 33.22, 31.39, 24.63, 23.11. ESI-MS: calcd for $C_{32}H_{40}N_2O_5$ = 532.3, found [M+H]+ = 533.3.

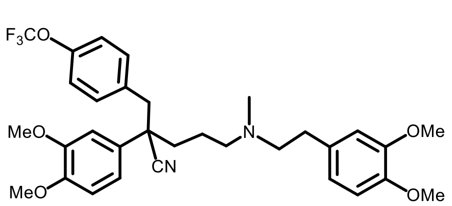

PhA40. Yellow oil, 30% yield. ^{1}H NMR (500 MHz, $CDCl_3$) δ 7.04 (d, *J* = 8.1 Hz, 1H), 6.99 – 6.93 (m, 1H), 6.88 – 6.63 (m, 8H), 3.96 – 3.85 (m, 12H), 3.19 – 3.04 (m, 1H), 2.97 – 2.71 (m, 3H), 2.68 – 2.60 (m, 2H), 2.56 – 2.45 (m, 2H), 2.31 (s, 3H), 2.17 (ddd, *J* = 16.9, 11.9, 4.4 Hz, 1H), 2.06 (td, *J* = 13.9, 13.0, 4.5 Hz, 1H), 1.72 (dt, *J* = 12.9, 7.3 Hz, 1H), 1.44 (dt, *J* = 12.8, 6.8 Hz, 1H). ^{13}C NMR (126 MHz, $CDCl_3$) δ 148.98, 148.88, 148.62, 147.40, 133.80, 131.64, 130.25, 129.40, 121.92, 120.81, 120.56, 120.41, 118.44, 112.12, 111.90, 111.53, 111.29, 111.20, 110.00, 59.36, 56.73, 55.93, 55.91, 51.43, 49.16, 47.36, 41.92, 37.23, 34.47, 29.82, 29.70, 23.04. ESI-MS: calcd for C32H37F3N2O5 = 586.3, found [M+H]+ = 587.2.

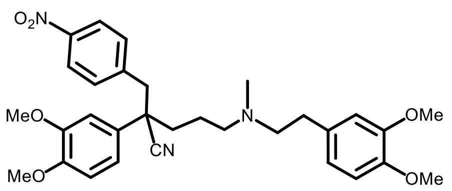

PhA41. Yellow oil, 10% yield. ^{1}H NMR (500 MHz, $CDCl_3$) □ 8.11 – 8.03 (m, 2H), 7.85 (s, 2H), 6.89 – 6.64 (m, 6H), 4.02 – 3.86 (m, 12H), 3.62 – 3.42 (m, 2H), 2.97 – 2.73 (m, 6H), 2.70 (s, 3H), 2.34 (s, 1H), 2.09 (s, 1H), 1.89 (s, 1H), 1.28 (s, 1H). ^{13}C NMR (126 MHz, $CDCl_3$) □ 149.02, 148.85, 148.63, 147.36, 137.55, 137.50, 132.97, 129.58, 129.44, 129.38, 126.04, 121.92, 120.55, 118.55, 117.05, 114.21, 112.14, 111.32, 109.90, 63.85, 59.38, 56.74, 55.91, 48.97, 47.58, 41.95, 37.36, 36.43, 33.18, 31.39, 23.05. ESI-MS: calcd for $C_{31}H_{37}N_3O_6$ = 547.3, found [M+H]+ = 548.2.

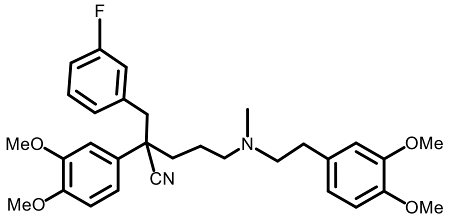

PhA42. Yellow oil, 30% yield. ^{1}H NMR (500 MHz, $CDCl_3$) δ 7.15 (td, *J* = 8.0, 6.0 Hz, 1H), 6.91 – 6.62 (m, 9H), 3.91 – 3.79 (m, 12H), 3.16 (d, *J* = 13.5 Hz, 1H), 3.05 (d, *J* = 13.5 Hz, 1H), 2.93 – 2.72 (m, 3H), 2.70 – 2.67 (m, 2H), 2.54 (dd, *J* = 9.1, 6.7 Hz, 1H), 2.46 – 2.33 (m, 1H), 2.23 (s, 3H), 2.13 – 1.97 (m, 1H), 1.66 (ddq, *J* = 18.6, 12.3, 5.9 Hz, 1H), 1.44 – 1.32 (m, 1H). ^{13}C NMR (126 MHz, $CDCl_3$) δ 149.01, 148.89, 148.85, 148.63, 147.35, 137.56, 137.51, 133.04, 129.60, 126.05, 126.02, 121.94, 120.56, 118.55, 117.06, 114.22, 112.14, 111.31, 109.90, 63.87, 59.41, 56.76, 55.92, 55.90, 48.98, 48.78, 47.60, 41.99, 37.38, 33.23, 23.09. $C_{31}H_{37}FN_2O4$ = 520.3, found [M+H]+ = 521.2.

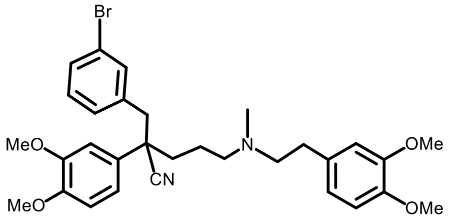

PhA43. Yellow oil, 25% yield. ^{1}H NMR (500 MHz, $CDCl_3$) δ 7.36 (dt, *J* = 8.2, 1.3 Hz, 1H), 7.23 – 7.18 (m, 1H), 7.11 – 7.06 (m, 1H), 6.91 (dt, *J* = 7.8, 1.3 Hz, 1H), 6.89 – 6.64 (m, 6H), 3.92 – 3.79 (m, 12H), 3.22 – 3.01 (m, 2H), 2.91 – 2.76 (m, 2H), 2.75 – 2.54 (m, 2H), 2.45 (tt, *J* = 12.8, 6.1 Hz, 2H), 2.26 (s, 3H), 2.16 – 1.99 (m, 2H), 1.69 (q, *J* = 6.3, 5.8 Hz, 1H), 1.46 – 1.37 (m, 1H). ^{13}C NMR (126 MHz, $CDCl_3$) δ 149.10, 148.89, 148.73, 147.40, 137.34, 133.30, 130.35, 129.55, 129.51, 128.92, 128.02, 121.95, 120.81, 120.57, 118.63, 112.15, 111.33, 111.30, 110.00, 59.41, 56.75, 55.95, 55.89, 48.96, 47.59, 41.96, 38.66, 37.14, 33.69, 33.19, 23.06. ESI-MS: calcd for $C_{31}H_{37}BrN_2O_4$ = 580.2, found [M+H]+ = 581.2.

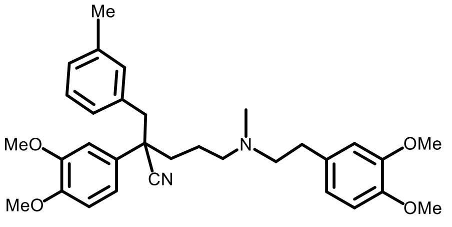

PhA44. Yellow oil, 42% yield. ^{1}H NMR (500 MHz, $CDCl_3$) δ 7.02 (t, *J* = 7.4 Hz, 1H), 6.96 (d, *J* = 7.6 Hz, 1H), 6.83 – 6.63 (m, 8H), 3.83 – 3.71 (m, 12H), 3.07 – 2.96 (m, 2H), 2.61 – 2.56 (m, 2H), 2.48 (t, *J* = 7.9 Hz, 2H), 2.41 – 2.29 (m, 2H), 2.20 (s, 3H), 2.17 (s, 3H), 1.99 (ddd, *J* = 25.4, 13.1, 7.5 Hz, 2H), 1.61 (dp, *J* = 13.2, 6.2 Hz, 1H), 1.34 (tt, *J* = 13.2, 6.3 Hz, 1H). ^{13}C NMR (126 MHz, $CDCl_3$) δ 148.83, 148.80, 148.43, 147.27, 137.39, 134.99, 133.10, 131.16, 130.20, 127.84, 127.79, 127.34, 122.20, 120.53, 118.59, 112.09, 111.28, 111.09, 110.06, 59.41, 56.81, 55.90, 55.86, 55.79, 49.00, 47.93, 46.10, 42.03, 37.02, 33.26, 23.16, 21.27. ESI-MS: calcd for $C_{32}H_{40}N_2O_4$ = 516.3, found [M+H]+ = 517.2.

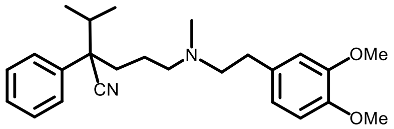

PhA45. Yellow oil, 48% yield. ^{1}H NMR (500 MHz, $CDCl_3$) δ 7.41 – 7.25 (m, 5H), 6.84 – 6.64 (m, 3H), 3.82 (d, *J* = 10.4 Hz, 6H), 2.67 – 2.51 (m, 2H), 2.51 – 2.40 (m, 2H), 2.31 (hept, *J* = 6.0 Hz, 2H), 2.19 – 1.96 (m, 5H), 1.85 (td, *J* = 13.1, 4.4 Hz, 1H), 1.57 – 1.50 (m, 1H), 1.30 – 1.01 (m, 4H), 0.74 (d, *J* = 6.7 Hz, ^{13}C NMR (126 MHz, $CDCl_3$) δ 148.78, 147.26, 138.19, 133.10, 128.70, 127.52, 126.34, 121.28, 120.56, 112.06, 111.24, 77.54, 77.29, 77.03, 59.33, 56.90, 55.88, 55.81, 53.70, 53.50, 42.00, 37.76, 35.43, 33.19, 23.40, 18.93, 18.57, 14.20.3H). ESI-MS: calcd for $C_{24}H_{32}N_2O_2$ = 394.6, found [M+H]+ = 395.2.

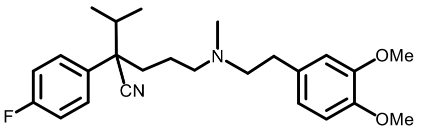

PhA46. Clear oil, 46% yield. ^{1}H NMR (400 MHz, $CDCl_3$) δ 7.34 – 7.20 (m, 2H), 7.19 (s, 2H), 6.72 (d, J = 8.6 Hz, 1H), 6.63 (d, J = 6.2 Hz, 2H), 3.79 (s, 3H), 3.78 (s, 3H), 2.59 (d, J = 8.4 Hz, 2H), 2.45 (dd, J = 8.1, 1.8 Hz, 2H), 2.29 (d, J = 4.1 Hz, 2H), 2.12 (s, 3H), 2.06 – 1.95 (m, 2H), 1.83 – 1.71 (m, 1H), 1.54 – 1.40 (m, 1H), 1.24 (d, J = 19.9 Hz, 2H), 1.11 (d, J = 6.7 Hz, 3H), 0.75 – 0.65 (m, 3H). ^{13}C NMR (101 MHz, $CDCl_3$) δ 148.83, 147.33, 134.88, 129.99, 127.89, 126.51, 124.76, 120.54, 112.08, 111.28, 59.41, 56.81, 55.93, 55.86, 53.65, 41.91, 37.86, 35.41, 33.19, 29.70, 23.39, 18.94, 18.55. ESI-MS: calcd for $C_{25}H_{33}FN_2O_2$ = 412.2, found [M+H]+ = 413.2.

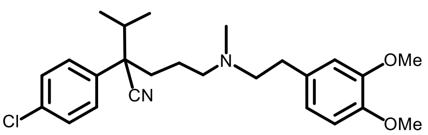

PhA47. Yellow oil, 49% yield. ^{1}H NMR (500 MHz, $CDCl_3$) δ 7.37 – 7.23 (m, 4H), 6.70 (qt, *J* = 9.8, 3.9 Hz, 2H), 3.84 (dd, *J* = 10.9, 2.4 Hz, 6H), 2.82 – 2.63 (m, 2H), 2.63 – 2.41 (m, 3H), 2.41 – 2.25 (m, 2H), 2.25 – 2.01 (m, 4H), 1.87 – 1.66 (m, 1H), 1.59 – 1.24 (m, 1H), 1.24 – 1.02 (m, 3H), 0.86 – 0.71 (m, 3H). ^{13}C NMR (126 MHz, $CDCl_3$) δ 148.79, 147.28, 136.89, 135.02, 133.67, 133.52, 133.39, 133.10, 128.88, 128.38, 128.34, 127.82, 121.21, 120.91, 120.55, 120.50, 119.92, 112.06, 111.25, 77.49, 77.24, 76.98, 59.84, 59.30, 57.25, 56.88, 56.82, 55.90, 55.82, 53.34, 48.66, 42.18, 42.04, 37.79, 35.38, 33.46, 33.22, 32.45, 28.85, 25.19, 23.45, 18.87, 18.68, 18.56, 18.05. ESI-MS: calcd for $C_{24}H_{31}ClN_2O_2$ = 429, found [M+H]+ = 429.1.

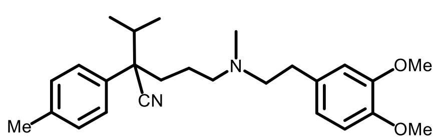

PhA48. Yellow oil, 61% yield. ^{1}H NMR (400 MHz, $CDCl_3$) δ 7.22 (d, J = 8.0 Hz, 2H), 7.14 (d, J = 7.9 Hz, 2H), 6.77 (d, J = 8.0 Hz, 1H), 6.69 (d, J = 7.5 Hz, 2H), 3.85 (s, 3H), 3.83 (s, 3H), 2.65 (dd, J = 9.5, 6.2 Hz, 2H), 2.55 – 2.43 (m, 2H), 2.35 (t, J = 6.6 Hz, 2H), 2.32 (s, 3H), 2.18 (s, 3H), 2.13 – 2.01 (m, 2H), 1.84 (td, J = 12.9, 4.5 Hz, 1H), 1.61 – 1.19 (m, 2H), 1.16 (d, J = 6.7 Hz, 3H), 0.76 (d, J = 6.7 Hz, 3H). ^{13}C NMR (101 MHz, $CDCl_3$) δ 148.80, 147.28, 137.12, 135.10, 133.08, 129.35, 126.23, 121.47, 120.56, 112.09, 111.26, 59.29, 56.92, 55.89, 55.82, 53.33, 42.00, 37.73, 35.40, 33.18, 23.38, 20.93, 18.92, 18.58. ESI-MS: calcd for $C_{26}H_{36}N_2O_2$ = 408.3, found [M+H]+ = 409.2.

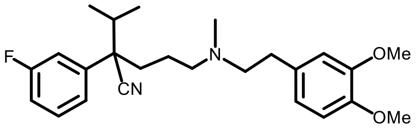

PhA49. Clear oil, 77% yield. ^{1}H NMR (400 MHz, $CDCl_3$) δ 7.20 (d, J = 9.9 Hz, 1H), 7.16 (t, J = 7.7 Hz, 1H), 7.12 – 7.05 (m, 1H), 7.05 – 6.99 (m, 1H), 6.75 – 6.69 (m, 1H), 6.66 – 6.58 (m, 2H), 3.79 (d, J = 2.5 Hz, 3H), 3.78 (s, 3H), 2.59 (d, J = 8.5 Hz, 2H), 2.45 (d, J = 7.8 Hz, 2H), 2.31 (d, J = 7.4 Hz, 2H), 2.12 (s, 3H), 2.08 – 1.96 (m, 2H), 1.89 – 1.79 (m, 1H), 1.79 – 1.68 (m, 1H), 1.53 – 1.38 (m, 1H), 1.11 (d, J = 6.7 Hz, 3H), 0.70 (d, J = 6.7 Hz, 3H). ^{13}C NMR (101 MHz, $CDCl_3$) δ 128.57, 128.27, 120.52, 112.06, 111.27, 55.93, 55.86, 53.67, 41.92, 37.77, 35.50, 21.59, 19.00, 18.57. ESI-MS: calcd for $C_{25}H_{33}FN_2O_2$ = 412.2, found [M+H]+ = 413.2.

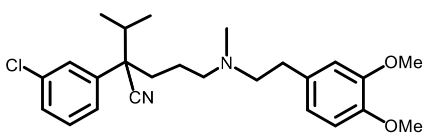

PhA50. Yellow oil, 49% yield. ^{1}H NMR (400 MHz, $CDCl_3$) δ 7.19 – 7.08 (m, 2H), 7.08 – 6.98 (m, 2H), 6.71 (d, J = 8.6 Hz, 1H), 6.62 (dt, J = 4.5, 2.3 Hz, 2H), 3.79 (s, 3H), 3.78 (s, 3H), 2.66 – 2.53 (m, 2H), 2.44 (dd, J = 9.5, 5.3 Hz, 2H), 2.31 (d, J = 7.4 Hz, 2H), 2.13 (s, 3H), 2.02 (ddd, J = 13.4, 5.5, 3.5 Hz, 2H), 1.86 – 1.78 (m, 1H), 1.76 (d, J = 4.5 Hz, 1H), 1.55 – 1.41 (m, 1H), 1.11 (d, J = 6.7 Hz, 3H), 0.70 (d, J = 6.7 Hz, 3H). ^{13}C NMR (101 MHz, $CDCl_3$) δ 128.57, 127.18, 120.52, 112.06, 55.94, 53.67, 37.78, 21.59, 18.57. ESI-MS: calcd for $C_{25}H_{33}ClN_2O_2$ = 428.2, found [M+H]+ = 429.1.

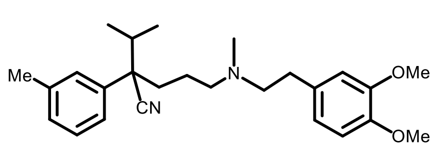

PhA51. Yellow oil, 50% yield. ^{1}H NMR (400 MHz, $CDCl_3$) δ 7.16 (t, J = 7.4 Hz, 1H), 7.11 (s, 1H), 7.07 – 7.03 (m, 1H), 7.03 – 6.99 (m, 1H), 6.72 (t, J = 4.2 Hz, 1H), 6.62 (dq, J = 4.1, 2.2 Hz, 2H), 3.79 (s, 3H), 3.78 (s, 3H), 2.66 – 2.53 (m, 2H), 2.43 (d, J = 8.2 Hz, 2H), 2.28 (s, 3H), 2.24 (d, J = 4.9 Hz, 2H), 2.11 (s, 3H), 2.05 – 1.95 (m, 2H), 1.78 (td, J = 13.3, 12.8, 4.6 Hz, 1H), 1.54 – 1.39 (m, 2H), 1.11 (d, J = 6.7 Hz, 3H), 0.70 (d, J = 6.7 Hz, 3H). ^{13}C NMR (101 MHz, $CDCl_3$) δ 148.81, 147.30, 138.35, 138.10, 132.95, 128.56, 128.26, 127.14, 123.24, 121.39, 120.53, 112.06, 111.27, 59.31, 56.86, 55.91, 55.83, 53.65, 41.92, 37.74, 35.46, 33.07, 23.33, 21.58, 19.00, 18.57. ESI-MS: calcd for $C_{26}H_{36}N_2O_2$ = 408.3, found [M+H]+ = 409.2.

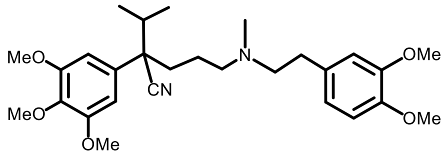

PhA52. Yellow oil, 42% yield. ^{1}H NMR (400 MHz, $CDCl_3$) δ 6.78 – 6.48 (m, 5H), 3.83 (ddd, *J* = 5.9, 2.7, 1.2 Hz, 15H), 2.71 – 2.40 (m, 4H), 2.34 (qq, *J* = 12.6, 7.1 Hz, 2H), 2.23 – 1.98 (m, 5H), 1.89 – 1.50 (m, 2H), 1.17 (dd, *J* = 6.7, 1.9 Hz, 4H), 0.78 (dd, *J* = 6.8, 1.8 Hz, 3H). ^{13}C NMR (101 MHz, $CDCl_3$) δ 153.28, 148.84, 147.32, 137.27, 133.99, 132.94, 121.22, 120.49, 112.03, 111.28, 103.59, 77.44, 77.12, 76.80, 60.87, 59.62, 56.91, 56.23, 55.91, 55.84, 54.08, 41.93, 37.93, 35.69, 33.15, 23.45, 19.07, 18.61. ESI-MS: calcd for $C_{28}H_{40}N_2O_5$ = 484.6, found [M+H]+ = 485.3.

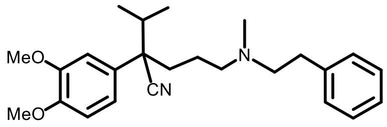

PhA53. Yellow oil, 31% yield. ^{1}H NMR (400 MHz, $CDCl_3$) δ 7.34 – 7.27 (m, 2H), 7.23 (d, J = 7.1 Hz, 1H), 7.17 (d, J = 7.5 Hz, 1H), 6.95 (dd, J = 8.6, 2.0 Hz, 1H), 6.91 – 6.84 (m, 2H), 3.91 (s, 3H), 3.89 (s, 3H), 2.91 – 2.78 (m, 2H), 2.72 (d, J = 9.0 Hz, 2H), 2.57 (s, 2H), 2.35 (s, 3H), 2.11 (tt, J = 11.5, 5.0 Hz, 4H), 1.69 – 1.58 (m, 1H), 1.21 (d, J = 6.7 Hz, 3H), 0.81 (d, J = 6.6 Hz, 3H). ^{13}C NMR (101 MHz, $CDCl_3$) δ 149.14, 148.42, 130.35, 128.66, 128.55, 126.42, 121.36, 118.73, 111.15, 109.57, 58.52, 56.29, 56.09, 55.89, 53.34, 41.31, 37.98, 35.34, 31.44, 29.70, 18.94, 18.64, 18.61. ESI-MS: calcd for $C_{25}H_{34}N_2O_2$ = 394.3, found [M+H]+ = 395.5.

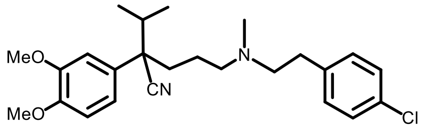

PhA54. Yellow oil, 52% yield. ^{1}H NMR (400 MHz, $CDCl_3$) δ 7.21 – 7.14 (m, 2H), 7.01 (d, J = 8.3 Hz, 2H), 6.83 (dd, J = 8.4, 2.3 Hz, 1H), 6.80 – 6.73 (m, 2H), 3.81 (s, 3H), 3.81 (s, 3H), 2.70 – 2.59 (m, 2H), 2.47 (t, J = 7.6 Hz, 2H), 2.34 (d, J = 8.7 Hz, 2H), 2.15 (s, 3H), 2.06 – 1.93 (m, 2H), 1.92 – 1.54 (m, 2H), 1.52 – 1.41 (m, 1H), 1.11 (d, J = 6.7 Hz, 3H), 0.72 (d, J = 6.7 Hz, 3H). ^{13}C NMR (101 MHz, $CDCl_3$) δ 149.03, 148.31, 130.56, 130.04, 128.48, 118.67, 111.08, 109.56, 58.66, 56.64, 56.02, 55.89, 53.34, 41.66, 37.95, 35.46, 32.49, 29.70, 23.00, 18.96, 18.60. ESI-MS: calcd for $C_{25}H_{33}ClN_2O_2$ =428.2, found [M+H]+ =429.5.

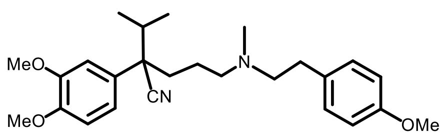

PhA55. Yellow oil, 30% yield. ^{1}H NMR (400 MHz, $CDCl_3$) δ 7.08 (d, J = 8.2 Hz, 2H), 6.99 – 6.90 (m, 1H), 6.87 (d, J = 6.3 Hz, 2H), 6.83 (d, J = 7.9 Hz, 2H), 3.90 (s, 3H), 3.89 (s, 3H), 3.79 (s, 3H), 2.70 (t, J = 8.6 Hz, 2H), 2.54 (t, J = 7.2 Hz, 2H), 2.49 – 2.32 (m, 2H), 2.23 (s, 3H), 2.09 (dt, J = 13.4, 5.8 Hz, 2H), 1.87 (dt, J = 7.1, 4.0 Hz, 2H), 1.63 – 1.51 (m, 1H), 1.19 (d, J = 6.7 Hz, 3H), 0.80 (d, J = 6.7 Hz, 3H). ^{13}C NMR (101 MHz, $CDCl_3$) δ 157.96, 149.02, 148.29, 130.61, 129.57, 121.44, 118.69, 113.82, 111.09, 109.58, 59.24, 56.71, 56.01, 55.88, 55.24, 53.43, 53.35, 41.78, 37.93, 35.52, 32.33, 29.70, 23.07, 18.96, 18.61, 1.02. ESI-MS: calcd for $C_{26}H_{36}N_2O_3$ =424.2, found [M+H]+ =425.5.

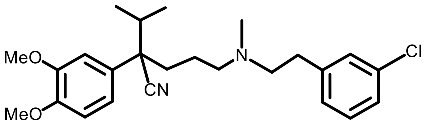

PhA56. Yellow oil, 44% yield. ^{1}H NMR (400 MHz, $CDCl_3$) δ 7.24 – 7.05 (m, 4H), 6.87 (dd, J = 8.4, 2.2 Hz, 1H), 6.85 – 6.74 (m, 2H), 3.84 (s, 3H), 3.81 (s, 3H), 2.83 (d, J = 7.7 Hz, 2H), 2.72 (d, J = 8.2 Hz, 2H), 2.64 (s, 2H), 2.34 (s, 3H), 2.04 (dp, J = 20.0, 6.7, 5.7 Hz, 4H), 1.59 (s, 1H), 1.12 (d, J = 6.6 Hz, 3H), 0.72 (d, J = 6.7 Hz, 3H). ^{13}C NMR (101 MHz, $CDCl_3$) δ 130.02 (d, J = 12.5 Hz), 128.80, 126.93, 118.73, 111.14, 109.48, 56.03 (d, J = 25.5 Hz), 38.03, 35.14, 18.94, 18.66. ESI-MS: calcd for $C_{25}H_{33}ClN_2O_2$ =428.2, found [M+H]+ =429.5.

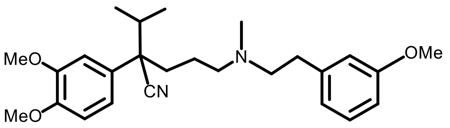

PhA57. Yellow oil, 23% yield. ^{1}H NMR (400 MHz, $CDCl_3$) δ 7.25 – 6.96 (m, 2H), 6.85 (dd, J = 8.4, 2.3 Hz, 1H), 6.82 – 6.73 (m, 2H), 6.67 (dt, J = 10.0, 2.5 Hz, 2H), 3.82 (s, 3H), 3.80 (s, 3H), 3.72 (s, 3H), 2.80 – 2.66 (m, 2H), 2.61 (d, J = 7.5 Hz, 2H), 2.47 (dq, J = 12.3, 6.9, 6.4 Hz, 2H), 2.23 (s, 3H), 1.63 – 1.44 (m, 1H), 1.12 (d, J = 6.7 Hz, 3H), 0.72 (d, J = 6.8 Hz, 3H). ^{13}C NMR (101 MHz, $CDCl_3$) δ 159.73, 149.10, 148.37, 129.50, 121.01, 118.70, 114.54, 111.13, 109.55, 56.41, 56.07, 55.88, 55.17, 53.33, 37.99, 35.36, 29.71, 18.95, 18.63. ESI-MS: calcd for $C_{26}H_{36}N_2O_3$ = 424.2, found [M+H]+ = 425.6.

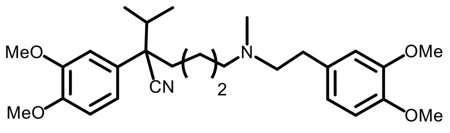

PhA58. Yellow oil, 62% yield. ^{1}H NMR (400 MHz, $CDCl_3$) δ 6.85-6.64 (m, 6H), 3.82 (s, 3H), 3.81 (s, 3H), 3.79 (s, 3H), 3.77 (s. 3H), 2.70-2.66 (m, 2H), 2.57-2.53 (m, 2H), 2.34 (t, J = 8Hz, 2H) 2.25 (s, 3H), 2.05-1.97 (m, 2H), 1.69 (td, J = 8Hz, 4Hz, 1H), 1.21-1.16 (m, 4H), 1.11 (d, J = 8Hz, 3H), 0.72 (d, J = 8Hz, 3H). ^{13}C NMR (101 MHz, $CDCl_3$) δ 148.95, 148.89, 148.22, 147.41, 132.59, 130.75, 121.54, 120.50, 118.69, 112.05, 111.34, 111.08, 109.69, 77.40, 77.08, 76.76, 59.57, 57.41, 55.93, 55.87, 53.42, 41.88, 37.84, 37.81, 33.03, 29.46, 27.18, 26.75, 25.55, 18.92, 18.58. ESI-MS: calcd for $C_{28}H_{40}N_2O_4$ = 468.3, found [M+H]+ = 469.1.

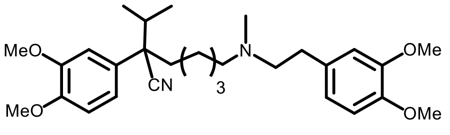

PhA59. Yellow oil, 61% yield. ^{1}H NMR (400 MHz, $CDCl_3$) δ 6.92 (dd, J = 8.5, 2.1 Hz, 1H), 6.88 – 6.83 (m, 2H), 6.80 (d, J = 8.6 Hz, 1H), 6.76 – 6.70 (m, 2H), 3.91 (s, 3H), 3.89 (s, 3H), 3.88 (s, 3H), 3.86 (s, 3H), 2.83 – 2.73 (m, 2H), 2.73 – 2.60 (m, 2H), 2.45 (t, J = 7.7 Hz, 2H), 2.36 (s, 3H), 2.17 – 2.01 (m, 2H), 1.80 (ddd, J = 13.5, 11.9, 4.5 Hz, 1H), 1.50 (dd, J = 5.9, 3.7 Hz, 2H), 1.28 (d, J = 11.7 Hz, 4H), 1.19 (d, J = 6.6 Hz, 3H), 0.80 (d, J = 6.7 Hz, 3H). ^{13}C NMR (101 MHz, $CDCl_3$) δ 148.97, 148.92, 148.25, 147.49, 130.66, 121.52, 120.50, 118.65, 112.02, 111.33, 111.09, 109.66, 59.37, 57.15, 56.04, 55.93, 55.88, 53.42, 41.71, 37.89, 37.74, 32.75, 31.44, 30.19, 29.69, 27.29, 26.25, 25.42, 18.93, 18.59. ESI-MS: calcd for $C_{29}H_{42}N_2O_4$ = 482.3, found [M+H]+ = 483.6.

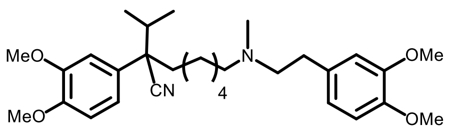

PhA60. Yellow oil, 58% yield. ^{1}H NMR (400 MHz, $CDCl_3$) δ 6.91-6.72 (m, 6H), 3.89 (s, 3H), 3.87 (s, 3H), 3.86 (s, 3H), 3.84 (s. 3H), 2.77-2.73 (m, 2H), 2.64-2.60 (m, 2H), 2.41 (t, J = 8Hz, 2H) 2.32 (s, 3H), 2.12-2.04 (m, 2H), 1.80-1.69 (m, 2H), 1.49-1.41 (m, 3H), 1.28-1.22 (m, 4H) 1.18 (d, J = 8Hz, 3H), 0.79 (d, J = 8Hz, 3H). ^{13}C NMR (101 MHz, $CDCl_3$) δ 148.93, 148.87, 148.20, 147.39, 132.55, 130.90, 130.74, 128.81, 121.52, 120.50, 118.68, 112.03, 111.32, 111.06, 109.65, 65.53, 59.52, 57.36, 56.00, 55.91, 55.85, 53.41, 41.84, 37.83, 37.79, 30.55, 29.44, 27.17, 25.54, 19.17, 18.92, 18.57, 13.72. ESI-MS: calcd for $C_{30}H_{44}N_2O_4$ = 496.3, found [M+H]+ = 497.4.

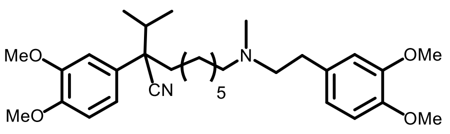

PhA61. Yellow oil, 68% yield. ^{1}H NMR (400 MHz, $CDCl_3$) δ 6.91 (dd, J = 8.4, 2.2 Hz, 1H), 6.88 – 6.83 (m, 2H), 6.80 (d, J = 8.7 Hz, 1H), 6.77 – 6.72 (m, 2H), 3.90 (s, 3H), 3.89 (s, 3H), 3.87 (s, 3H), 3.85 (s, 3H), 2.86 – 2.79 (m, 2H), 2.75 – 2.68 (m, 2H), 2.51 (dd, J = 6.3, 4.0 Hz, 2H), 2.40 (s, 3H), 2.15 – 2.02 (m, 2H), 1.82 – 1.69 (m, 1H), 1.53 (s, 2H), 1.41 – 1.22 (m, 8H), 1.18 (d, J = 6.6 Hz, 3H), 0.79 (d, J = 6.7 Hz, 3H). ^{13}C NMR (101 MHz, $CDCl_3$) δ 148.93, 148.19, 147.52, 130.77, 121.56, 120.51, 118.69, 112.02, 111.34, 111.05, 109.64, 59.25, 57.31, 56.01, 55.93, 55.88, 53.43, 41.61, 37.86, 37.81, 32.59, 29.68, 29.43, 29.12, 27.21, 25.49, 18.92, 18.58. ESI-MS: calcd for $C_{31}H_{46}N_2O_4$ = 510.3, found [M+H]+ = 511.4.

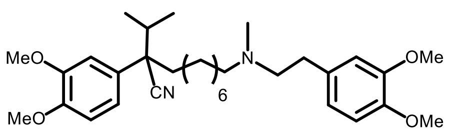

PhA62. Yellow oil, 59% yield. ^{1}H NMR (400 MHz, $CDCl_3$) δ 6.93-6.74 (m, 6H), 3.91 (s, 3H), 3.90 (s, 3H), 3.89 (s, 3H), 3.87 (s. 3H), 2.93-2.89 (m, 2H), 2.85-2.80 (m, 2H), 2.62 (t, J = 8Hz, 2H) 2.50 (s, 3H), 2.13-2.04 (m, 3H), 1.81-1.70 (m, 2H), 1.63-1.60 (m, 3H), 1.30-1.19 (m, 10H), 0.80 (d, J = 8Hz, 3H). ^{13}C NMR (101 MHz, $CDCl_3$) δ 149.03, 148.93, 147.70, 130.78, 121.56, 120.54, 120.54, 118.72, 116.30, 112.03, 111.40, 111.07, 109.68, 57.12, 56.03, 55.93, 53.44, 45.69, 41.27, 37.87, 37.82, 32.14, 29.70, 29.44, 29.21, 29.15, 27.16, 25.77, 25.50, 22.51, 18.93, 18.59. ESI-MS: calcd for $C_{32}H_{48}N_2O_4$ = 524.4, found [M+H]+ = 525.3.

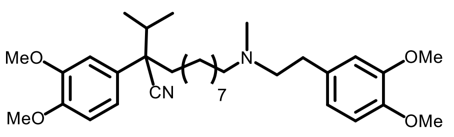

PhA63. Yellow oil, 45% yield. ^{1}H NMR (400 MHz, $CDCl_3$) δ 6.93 – 6.55 (m, 6H), 3.95 – 3.74 (m, 12H), 2.93 – 2.47 (m, 5H), 2.47 – 2.16 (m, 4H), 2.16 – 1.91 (m, 2H), 1.73 (ddd, *J* = 13.5, 12.1, 4.5 Hz, 1H), 1.53 – 0.69 (m, 17H). ^{13}C NMR (101 MHz, $CDCl_3$) δ 148.97, 148.89, 148.23, 147.39, 132.92, 130.87, 121.63, 120.65, 120.56, 118.77, 112.09, 111.43, 111.34, 111.07, 109.68, 59.59, 57.55, 56.04, 55.96, 55.91, 55.88, 53.47, 41.97, 37.89, 33.16, 29.74, 29.60, 29.51, 29.49, 29.30, 27.51, 26.96, 25.59, 18.97, 18.63. ESI-MS: calcd for $C_{33}H_{50}N_2O_4$ = 538.4, found [M+H]+ = 539.3.

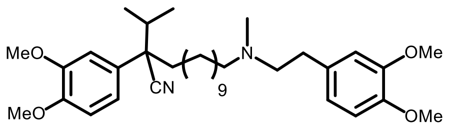

PhA64. Yellow oil, 58% yield. ^{1}H NMR (400 MHz, $CDCl_3$) δ 6.85-6.66 (m, 6H), 3.83 (s, 3H), 3.82 (s, 3H), 3.81 (s, 3H), 3.79 (s. 3H), 2.96-2.93 (m, 4H), 2.77-2.75 (m, 2H), 2.58 (s, 3H), 2.05-1.96 (m, 2H), 1.73-1.60 (m, 4H), 1.21-1.19 (m, 14H), 1.11 (d, J = 8Hz, 3H), 0.79 (d, J = 8Hz, 3H). ^{13}C NMR (101 MHz, $CDCl_3$) δ 148.95, 147.99, 146.56, 146.19, 130.76, 121.60, 120.57, 118.72, 112.01, 111.50, 111.10, 109.70, 108.70, 66.21, 65.57, 61.91, 55.95, 53.45, 50.89, 49.31, 48.66, 37.88, 30.58, 29.70, 29.35, 29.04, 27.13, 26.86, 25.47, 25.30, 22.69, 21.77, 21.72, 18.93, 18.59. ESI-MS: calcd for $C_{35}H_{54}N_2O_4$ = 566.4, found [M+H]+ = 567.2.

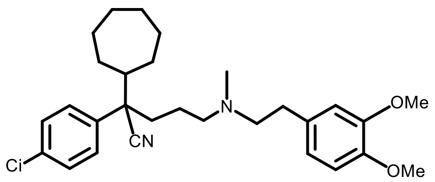

PhA65. Yellow oil, 57% yield. ^{1}H NMR (400 MHz, $CDCl_3$) δ 7.32 – 7.16 (m, 4H), 6.77 – 6.48 (m, 3H), 3.78 (d, *J* = 5.9 Hz, 6H), 2.64 (dd, *J* = 9.2, 5.7 Hz, 2H), 2.58 – 2.47 (m, 2H), 2.45 – 2.27 (m, 2H), 2.18 (s, 3H), 2.11 – 1.87 (m, 2H), 1.81 (ddt, *J* = 13.2, 8.6, 4.3 Hz, 2H), 1.54 (s, 1H), 1.54 – 0.98 (m, 12H). ^{13}C NMR (101 MHz, $CDCl_3$) δ 149.29, 147.87, 137.51, 133.83, 132.62, 129.35, 128.36, 122.02, 120.93, 112.43, 111.70, 59.33, 56.92, 56.31, 56.26, 53.62, 48.90, 42.08, 35.69, 33.01, 30.54, 30.41, 28.33, 27.80, 27.35, 27.12, 23.19. ESI-MS: calcd for $C_{29}H_{39}ClN_2O_2$ = 482.3, found [M+H]+ = 483.2.

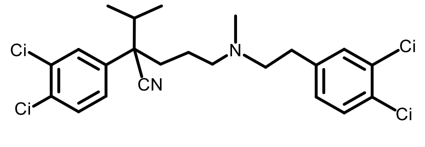

PhA66. Orange oil, 22% yield. ^{1}H NMR (400 MHz, $CDCl_3$) δ 7.26 (s, 2H), 7.3(d, J = 8Hz, 1H), 7.20 (s, 1H), 7.12(dd, J = 8Hz, 4Hz, 1H), 6.93(dd, J = 8Hz, 4Hz, 1H), 2.62 (t, J = 8Hz, 2H), 2.47 (t, J = 8Hz, 2H), 2.35-2.25 (m, 2H), 2.12 (s, 3H), 1.73-1.64 (td, J = 8Hz, 4Hz, 1H), 1.12-1.18 (m, 4H), 1.09 (d, J = 8Hz, 3H), 0.70 (d, J = 8Hz, 3H). ^{13}C NMR (101 MHz, $CDCl_3$) δ 140.38, 138.64, 130.69, 130.26, 128.44, 128.23, 125.85, 58.22, 56.44, 53.23, 41.32, 37.92, 35.13, 32.29, 31.93, 29.70, 29.66, 29.36, 23.00, 22.70, 18.86, 18.49, 14.13. ESI-MS: calcd for $C_{23}H_{26}Cl_4N_2$ = 470.1, found [M+H]+ = 471.4

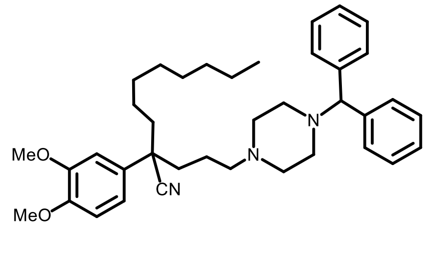

PhA67. Brown oil, 49% yield. ^{1}H NMR (400 MHz, $CDCl_3$) δ 7.40 – 7.06 (m, 10H), 6.90 – 6.72 (m, 3H), 4.16 (s, 1H), 3.80 (d, *J* = 10.4 Hz, 6H), 2.63 – 2.19 (m, 9H), 1.99 – 1.49 (m, 7H), 1.42 – 0.94 (m, 16H), 0.78 (t, *J* = 6.9 Hz, 3H). ^{13}C NMR (101 MHz, $CDCl_3$) δ 128.55, 127.84, 127.06, 124.25, 118.94, 115.00, 112.34, 111.01, 107.54, 100.65, 95.91, 82.66, 60.43, 56.06, 55.91, 47.79, 31.78, 30.52, 29.40, 29.23, 28.67, 24.96, 22.61, 22.07, 14.08. ESI-MS: calcd for $C_{38}H_{51}N_3O_2$ = 581.4, found [M+H]+ = 582.7.

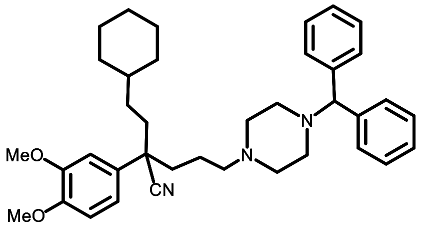

PhA68. Yellow oil, 55% yield. ^{1}H NMR (400 MHz, $CDCl_3$) δ 7.41 – 7.05 (m, 12H), 6.86 – 6.70 (m, 3H), 4.16 (d, *J* = 26.4 Hz, 1H), 3.78 (d, *J* = 7.7 Hz, 6H), 2.55 – 2.10 (m, 10H), 1.99 – 1.68 (m, 4H), 1.55 (ddd, *J* = 19.0, 9.3, 3.7 Hz, 6H), 1.32 – 0.61 (m, 11H). ^{13}C NMR (101 MHz, $CDCl_3$) δ 149.82, 149.05, 143.34, 131.62, 129.39, 129.19, 128.62, 128.56, 127.66, 123.42, 118.95, 111.94, 109.84, 76.88, 58.39, 56.73, 56.61, 53.93, 52.29, 48.52, 39.66, 39.37, 38.30, 33.91, 33.73, 33.33, 27.23, 26.94, 23.09. ESI-MS: calcd for $C_{38}H_{49}N_3O2$ = 579.4, found [M+H]+ = 581.1

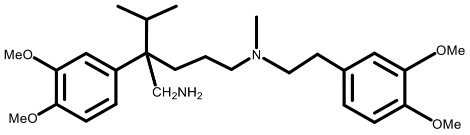

PhA69. Colourless oil, 78% yield. ^{1}H NMR (500 MHz, $CDCl_3$) δ 6.83 (d, *J* = 8.7 Hz, 1H), 6.81 – 6.69 (m, 5H), 4.36 (br, 2H), 3.92 – 3.84 (m, 9H), 3.82 (s, 3H), 3.26 (s, 2H), 3.03 (q, *J* = 7.3 Hz, 1H), 2.88 – 2.76 (m, 3H), 2.74 – 2.60 (m, 2H), 2.46 (s, 3H), 1.98 – 1.84 (m, 2H), 1.52 (q, *J* = 8.5, 7.9 Hz, 2H), 1.33 (t, *J* = 7.3 Hz, 1H), 0.76 (dd, *J* = 16.8, 6.8 Hz, 6H). ^{13}C NMR (126 MHz, $CDCl_3$) δ 149.02, 148.47, 147.61, 147.55, 133.10, 131.48, 120.61, 120.26, 112.10, 111.57, 111.33, 110.74, 59.25, 57.79, 56.16, 55.90, 55.76, 46.74, 45.94, 44.10, 41.65, 33.55, 32.16, 30.28, 20.90, 18.00, 17.83. ESI-MS: calcd for $C_{27}H_{42}N_2O_4$ = 458.3, found $[M+H]^+$ = 459.1

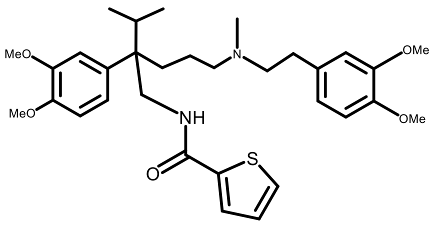

PhA70. Brown oil, 64% yield. ^{1}H NMR (400 MHz, $CDCl_3$) δ 7.78 – 7.14 (m, 6H), 7.14 – 6.54 (m, 9H), 4.16 (s, 1H), 3.97 – 3.69 (m, 12H), 3.58 (dd, *J* = 14.2, 4.6 Hz, 1H), 3.10 – 2.74 (m, 6H), 2.57 (d, *J* = 2.6 Hz, 3H), 1.82 (h, *J* = 8.4, 7.8 Hz, 4H), 1.20 (q, *J* = 14.7, 9.4 Hz, 6H), 0.75 (t, *J* = 6.5 Hz, 8H). ^{13}C NMR (101 MHz, $CDCl_3$) δ 161.70, 149.50, 148.23, 130.22, 128.23, 127.87, 120.70, 119.25, 113.00, 111.55, 64.75, 62.64, 56.27, 56.03, 55.94, 55.81, 47.40, 42.92, 42.10, 34.12, 27.34, 18.19, 17.88. ESI-MS: calcd for $C_{32}H_{44}N_2O_5S$ = 568.3, found $[M+H]^+$ = 569.5

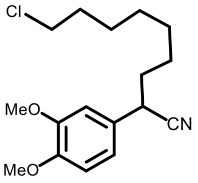

Int 4 for AZ-2. Pale yellow oil, 43% yield. ^{1}H NMR (400 MHz, $CDCl_3$) δ 6.73 (m, 3H), 3.76–3.73 (d, 6H), 3.62 (t, 1H, *J* = 6.8 Hz), 3.38 (t, 2H, *J* = 6.8 Hz), 1.81–1.68 (m, 2H), 1.58 (m, 2H), 1.29–1.14 (m, 8H). ^{13}C NMR (101 MHz, $CDCl_3$) δ 149.27, 148.68, 128.43, 121.08, 119.46, 111.40, 110.28, 55.87, 55.83, 44.95, 36.75, 35.71, 32.42, 28.70, 28.47, 26.84, 26.61.

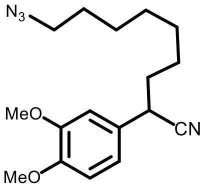

Int 5 for AZ-2. Yellow oil, 68% yield. ^{1}H NMR (400 MHz, $CDCl_3$) δ 6.77–6.74 (m, 3H), 3.79 (d, 6H, *J* = 9.6 Hz), 3.63 (t, 1H, *J* = 6.4 Hz), 3.16 (t, 2H, *J* = 6.8 Hz), 1.80 (m, 2H), 1.48 (m, 2H), 1.38 (m, 2H), 1.25 (s, 6H). ^{13}C NMR (101 MHz, $CDCl_3$) δ 149.35, 148.77, 128.40, 119.53, 111.42, 110.25, 55.97, 55.93, 51.34, 36.92, 35.83, 29.67, 28.82, 28.79, 28.73, 26.90, 26.54.

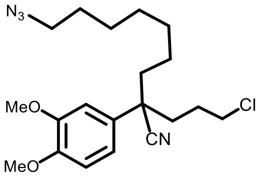

Int 6 for AZ-2. Yellow oil, 87% yield. ^{1}H NMR (400 MHz, $CDCl_3$) δ 6.89–6.79 (m, 3H), 3.83–3.81 (d, 6H, *J* = 7.2 Hz), 3.43–3.40 (t, 2H, *J* = 5.6 Hz), 3.17–3.13 (t, 2H, *J* = 6.8 Hz), 2.01 (m, 2H), 1.89 (m, 2H), 1.76 (m, 1H), 1.47 (m, 3H), 1.39 (m, 1H), 1.18 (m, 7H). ^{13}C NMR (101 MHz, $CDCl_3$) δ 149.25, 148.55, 130.26, 122.38, 118.28, 111.30, 108.97, 56.04, 55.91, 51.34, 47.40, 44.53, 41.41, 38.19, 29.20, 28.78, 28.71, 28.30, 26.53, 25.10.

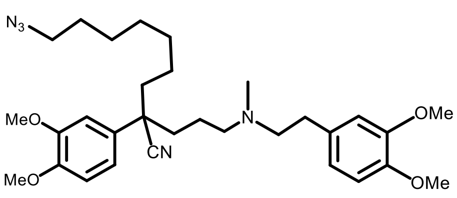

AZ-2. Yellow oil, 24% yield. ^{1}H NMR (400 MHz, $CDCl_3$) δ 6.90–6.61 (m, 6H), 3.79 (m, 12H), 3.17–3.14 (t, 2H, *J* = 6.8 Hz), 2.81 (m, 4H), 2.67 (s, 2H), 2.41 (s, 3H), 2.09–1.74 (m, 6H), 1.48 (m, 3H), 1.37 (m, 2H), 1.05 (m, 2H), 0.81 (m, 1H). ^{13}C NMR (101 MHz, $CDCl_3$) δ 149.28, 149.06, 148.54, 147.75, 130.92, 130.40, 128.83, 120.56, 118.23, 112.01, 111.40, 111.30, 109.05, 65.56, 58.57, 56.14, 55.93, 55.91, 51.36, 47.79, 41.26, 41.08, 38.34, 30.56, 29.19, 28.79, 28.72, 26.53, 25.15, 19.18, 13.72. ESI-MS: calcd for $C_{31}H_{45}N_5O_4$ = 551.4, found [M+H]+ = 552.7
